# Core outcome measures for interventions to prevent or slow the progress of dementia for people living with mild to moderate dementia: Systematic review and consensus recommendations

**DOI:** 10.1371/journal.pone.0179521

**Published:** 2017-06-29

**Authors:** Lucy Webster, Derek Groskreutz, Anna Grinbergs-Saull, Rob Howard, John T. O’Brien, Gail Mountain, Sube Banerjee, Bob Woods, Robert Perneczky, Louise Lafortune, Charlotte Roberts, Jenny McCleery, James Pickett, Frances Bunn, David Challis, Georgina Charlesworth, Katie Featherstone, Chris Fox, Claire Goodman, Roy Jones, Sarah Lamb, Esme Moniz-Cook, Justine Schneider, Sasha Shepperd, Claire Surr, Jo Thompson-Coon, Clive Ballard, Carol Brayne, Alistair Burns, Linda Clare, Peter Garrard, Patrick Kehoe, Peter Passmore, Clive Holmes, Ian Maidment, Louise Robinson, Gill Livingston

**Affiliations:** 1 Division of Psychiatry, University College London, London, United Kingdom; 2 Division of Psychology and Language Sciences, University College London, London, United Kingdom; 3 Alzheimer’s Society, London, United Kingdom; 4 Department of Psychiatry, University of Cambridge, Cambridge, United Kingdom; 5 ScHARR, University of Sheffield, Sheffield, United Kingdom; 6 Centre for Dementia Studies, Brighton and Sussex Medical School, Brighton, United Kingdom; 7 Dementia Services Development Centre Wales, Bangor University, Bangor, United Kingdom; 8 Faculty of Medicine, School of Public Health, Imperial College London, London, United Kingdom; 9 Department of Psychiatry and Psychotherapy, Ludwig-Maximilians-Universität München, Munich, Germany; 10 Department of Psychiatry and Psychotherapy, Technische Universität München, Munich, Germany; 11 Cambridge Institute of Public Health, University of Cambridge, Cambridge, United Kingdom; 12 International Consortium for Health Outcomes Measurement, London, United Kingdom; 13 Oxford Health NHS Foundation Trust, Oxford, United Kingdom; 14 Centre for Research in Primary and Community Care, University of Hertfordshire, Hatfield, United Kingdom; 15 Personal Social Services Research Unit, University of Manchester, Manchester, United Kingdom; 16 Research Department of Clinical, Educational, and Health Psychology, University College London, London, United Kingdom; 17 School of Healthcare Sciences, Cardiff University, Cardiff, United Kingdom; 18 Norwich Medical School, University of East Anglia, Norwich, United Kingdom; 19 Research Institute for the Care of Older People (RICE), University of Bath, Bath, United Kingdom; 20 Warwick Clinical Trials Research Unit, University of Warwick, Warwick, United Kingdom; 21 Faculty of Health and Social Care, University of Hull, Hull, United Kingdom; 22 Institute of Mental Health, University of Nottingham, Nottingham, United Kingdom; 23 Nuffield Department of Population Health, University of Oxford, Oxford, United Kingdom; 24 School of Health & Community Studies, Leeds Beckett University, Leeds, United Kingdom; 25 PenCLAHRC, University of Exeter Medical School, Exeter, United Kingdom; 26 Wolfson Centre for Age-Related Diseases, King’s College London, London, United Kingdom; 27 Institute of Brain, Behaviour and Mental Health, University of Manchester, Manchester, United Kingdom; 28 School of Psychology, University of Exeter, Exeter, United Kingdom; 29 Neuroscience Research Centre, St. George's, University of London, London, United Kingdom; 30 School of Clinical Sciences, University of Bristol, Bristol, United Kingdom; 31 Centre for Public Health, Queen's University Belfast, Belfast, United Kingdom; 32 School of Medicine, University of Southampton, Southampton, United Kingdom; 33 Aston Research Centre for Healthy Ageing, Aston University, Birmingham, United Kingdom; 34 Institute of Health and Society, Newcastle University, Newcastle upon Tyne, United Kingdom; 35 North Thames CLAHRC, London, United Kingdom; 36 Camden and Islington NHS Foundation Trust, London, United Kingdom; University of Glasgow, UNITED KINGDOM

## Abstract

**Background:**

There are no disease-modifying treatments for dementia. There is also no consensus on disease modifying outcomes. We aimed to produce the first evidence-based consensus on core outcome measures for trials of disease modification in mild-to-moderate dementia.

**Methods and findings:**

We defined disease-modification interventions as those aiming to change the underlying pathology. We systematically searched electronic databases and previous systematic reviews for published and ongoing trials of disease-modifying treatments in mild-to-moderate dementia. We included 149/22,918 of the references found; with 81 outcome measures from 125 trials. Trials involved participants with Alzheimer’s disease (AD) alone (n = 111), or AD and mild cognitive impairment (n = 8) and three vascular dementia. We divided outcomes by the domain measured (cognition, activities of daily living, biological markers, neuropsychiatric symptoms, quality of life, global). We calculated the number of trials and of participants using each outcome. We detailed psychometric properties of each outcome. We sought the views of people living with dementia and family carers in three cities through Alzheimer’s society focus groups. Attendees at a consensus conference (experts in dementia research, disease-modification and harmonisation measures) decided on the core set of outcomes using these results. Recommended core outcomes were cognition as the fundamental deficit in dementia and to indicate disease modification, serial structural MRIs. Cognition should be measured by Mini Mental State Examination or Alzheimer's Disease Assessment Scale-Cognitive Subscale. MRIs would be optional for patients. We also made recommendations for measuring important, but non-core domains which may not change despite disease modification.

**Limitations:**

Most trials were about AD. Specific instruments may be superseded. We searched one database for psychometric properties.

**Interpretation:**

This is the first review to identify the 81 outcome measures the research community uses for disease-modifying trials in mild-to-moderate dementia. Our recommendations will facilitate designing, comparing and meta-analysing disease modification trials in mild-to-moderate dementia, increasing their value.

**Trial registration:**

PROSPERO no. CRD42015027346.

## Introduction

The number of people with dementia is increasing globally. In the UK it is estimated that a third of people born in 2015 will develop dementia during their lifetime [1]. People living with dementia are currently offered management to improve their symptoms but there are no disease modifying treatments to halt or delay the progression of underlying disease pathologies. Between 1998 and 2012, 101 new potential pharmacological treatments for Alzheimer’s disease (AD), the most common type of dementia, were trialled internationally; four of these received regulatory approval, and these were symptomatic rather than disease modifying treatments [2]. At the G8 dementia summit in December 2013, the eight participating countries including the United Kingdom (UK), committed to identifying a disease modifying treatment by 2025 [3]. If a treatment that could delay the progression of all mild-to-moderate dementia by 50% became available in 2020, it is estimated that this could reduce the percentage of people living with dementia who are at the severe stage from 14% to 2% by 2050 [4].

It is important that the outcomes measured in disease modifying trials are appropriate, sensitive to change, and clinically meaningful to improve the possibility of identifying an effective treatment [5–7]. It is also essential for outcome measures used across trials to be harmonised as the large variation of measures currently used makes trials more difficult to design, and impedes the direct comparison of trials and their meta-analysis [8]. A recently published review found a large number of outcomes used across over 600 dementia trials, including many non-standardised measures, further emphasising the need for harmonisation of outcomes [9]. The difficulties in design can be highlighted when trials come to funding boards or for review and there is considerable dissent among referees about the choice of instrument and what outcomes are needed for a good quality trial.

Consequently, there is a need for consensus amongst dementia researchers about an agreed set of core outcome measures to use in future disease modification trials. This would enhance interpretation of data across trials and enable combination of small data sets to better inform practice. This requires not only the identification of valid and reliable measures but attention to their acceptability for both people living with dementia and the research community, so they facilitate rather than impede recruitment and retention of people with dementia and their families. This includes consideration of burden in terms of time, inconvenience and possible distress they trigger in patients and their families.

In response, this project, funded by the UK National Institute of Health Research (NIHR), set out to identify and establish a core set of outcomes to be used in future disease modification NIHR funded trials in mild-to-moderate dementia based on measures already used, and to inform the design of disease modification trials more widely [10].

We began by appraising other core outcome sets for dementia which members of the project team had been involved in. These were developed from other perspectives than disease modification. We then conducted a systematic review to identify the outcome measures used across published and ongoing disease modification trials, and collated psychometric data for each measure. We followed this by interviews and emails with people living with dementia and family carers. Finally, we brought this information together in a consensus conference attended by leading experts from the dementia research community to decide on a core set of outcomes.

## Methods

### Other outcome sets

The other core outcome sets we appraised included one funded by the Alzheimer’s Society (AS) led by C Brayne and LL, an EU Joint Programme–Neurodegenerative Disease Research (JPND) funded study about outcomes used in psychosocial interventions in dementia, led by GM [11, 12], and a review of measures which are important to patients with dementia funded by International Consortium for Health Outcomes Measurement Working Group (ICHOM), led by CR [13].

### Systematic review

#### Search strategy and selection criteria

We registered the systematic review protocol as PROSPERO no. CRD42015027346, and the project with the Core Outcome Measures in Effectiveness Trials initiative (COMET; http://www.comet-initiative.org/studies/details/819?result=true). After registration we changed the protocol and additionally searched for ongoing as well as published trials on clinical trials registries. We sought to identify published and ongoing disease modification trials, which we defined as interventions aiming to change the underlying pathology of the disease, as opposed to solely aiming to treat symptoms of dementia.

We conducted database searches using the key words for MEDLINE (via OvidSP; Appendix A in S1 File) and searched ALOIS, CENTRAL, CINAHL, EMBASE, LILACS, and PsycINFO for published trials from database inception to 11^th^ December 2015. We used the same strategy across databases, and adapted it for ALOIS. Additionally, we searched for ongoing trials on clinical trials registries (ISRCTN and clinicaltrials.gov) on 22^nd^ January 2016 and 29^th^ January 2016 respectively. We limited database searches to English language, where possible, as we were searching for instruments available in English. We also hand-searched the reference lists of relevant systematic reviews, and of a systematic review of non-pharmacological interventions for dementia led by members of the group (LL).

We included trials that met all of the inclusion criteria:

1. Full text is written in English
2. Published in a peer reviewed journal article or a registered ongoing trial
3. At least some of the participants have clinically diagnosed mild or moderate dementia
4. Any length of intervention that aimed to modify the dementia disease
5. It is a randomised controlled trial (RCT) or clinical controlled trial (CCT) with:
  - the intervention directed at the person with dementia
  - the control or comparator arm comprising treatment as usual, no intervention, sham therapy, other therapy or placebo
6. At least one quantitative outcome measure related to disease modification in mild or moderate dementia

We excluded studies where all participants had severe dementia or mild cognitive impairment (MCI), and studies set in care homes, as few people with mild to moderate dementia are resident in care homes [14]. We also excluded trials if the outcomes were only qualitative, economic or related only to carers or drug levels.

Two reviewers (DG and LW) piloted the screening of titles and abstracts by independently screening the first 20 records and comparing decisions, and had no disagreements. Three raters (AGS, DG and LW) also piloted the screening of full texts for inclusion by independently screening the first 20 papers. They agreed on 80% of decisions, with no decision made by any rater to exclude a paper that was eventually included. There was disagreement about four papers concerning whether the intervention was aiming to modify the underlying pathology of the disease of dementia. We agreed to solve this by examining in detail how the intervention was described in the background section, as well as the aim of the intervention within the trial, and to discuss any trials where there was uncertainty between the raters and principal investigator (PI), GL.

#### Data extraction

We extracted characteristics from each trial, including location, dementia type and severity, how dementia diagnosis was made, participants’ sex and age, description of the intervention, number of participants (n), description of the comparator group, outcomes related to disease modification (primary or secondary if reported), and when outcomes were measured. We contacted authors for extra information where necessary. As our aim was to synthesise the trial outcome measures used, we did not assess the risk of bias of individual studies.

We divided outcomes into the domain measured (cognition, activities of daily living, biological markers (further divided into imaging, and fluid markers), neuropsychiatric symptoms, quality of life, and global). Initially we had not planned to consider global outcomes, but we added it as a separate outcome category as we found relevant measures. To assess accuracy of extracted data, two reviewers (DG and LW) independently extracted data from the same subsample of five trials; there were no differences between the raters’ extracted data. We used this exercise, and five additional papers, to pilot the data extraction tool and ensure all relevant data from trials were captured. As the raters were using the same criteria, all other trial data was then extracted by a single researcher, either DG or LW. We calculated how many trials used each outcome measure and the total number of participants across all trials. We searched Google Scholar to find a copy of each outcome measure in English, either the manual or a key paper relating to its development.

#### Outcome validation

We conducted separate iterative searches on Google Scholar using the name of the measure and psychometric terms to find information on the measures’ psychometric properties relevant to people living with dementia. We also consulted within our expert group. We sought information about:

- if the measure is validated in people with mild to moderate dementia for the outcome in which it is used as a measure
- if there are any relevant populations in which the measure is validated e.g. mild to moderate dementia, ethnic groups, languages
- unit of measurement
- sensitivity to change
- minimal clinically important difference
- reliability (inter-rater and test-retest)
- acceptability
- ceiling and floor effects
- average time taken to complete
- who fills in the questionnaire i.e. researcher through patient, family carer, paid carer or observation or self-complete
- any risks identified of use of the measure

### Patient and public involvement

We sought the views of people living with dementia and family carers (via the Alzheimer’s Society (AS) research network), on which of the domains they considered core, the acceptability of individual and packages of measures, and their thoughts on completing measures.

We held three focus groups, in Cambridge, London and Sheffield in February and March 2016. All were led by LW and AGS from AS. Champions within the group agreed to co-facilitate the focus groups. The AS had found that in previous focus groups including a clinician who uses these measures, and is therefore able to explain them, aids discussion allowing participants to ask specific questions. The clinicians in each group were GM in Sheffield, GL in London and JTO’B in Cambridge. Participants were emailed an information sheet and asked at the groups if they all consented to the session being recorded. LW or AGS listened to the recording before it was destroyed. Participants from the research network who lived within travelling distance of each focus group were invited. For the Cambridge focus group, we invited additional people from a local dementia and ageing research PPI group, as recommended by one of the member of our team. Focus groups lasted 1–2 hours.

We focussed on a different set of domains in each group, although there was an overlap between domains discussed. In addition to the audio-recording, we summarised the conversations on flipchart paper to allow the participants to generate conclusions as easily as possible throughout the discussion. This allowed volunteers to see the notes that we were recording, and refer to them when they felt it was necessary.

AGS and LW synthesised all the information from across the three focus groups in order to present the findings at the consensus conference. Firstly, one of them wrote up the notes from each of the focus groups into a report detailing the main recommendations and the considerations which had led to them. The other researcher would then listening to the audio recording to ensure the main points had been listed and read and edit the report to add anything that had been missed. We then combined the reports from across the three focus groups, and used this to conduct an email consultation with focus group participants, to allow them to comment on the domains they had not discussed and to make sure the conference presentations represented what had been said across the groups. We also asked participants if they had anything else to add.

We then ran a second email consultation on a report including recommendations made at the consensus conference. This was sent to Research Network volunteers who had not participated in a focus group. This consultation included volunteers who had expressed an interest in focus groups but were unable to attend, and those living in different areas, including Wales, Northern Ireland, and the Midlands.

### Consensus conference

We held a consensus conference in London to decide on the core set of outcomes using the systematic review results and PPI consultation. We invited all participants who agreed to collaborate on the project at the protocol stage. Most of these were dementia academics who had been invited to the project funding call from the NIHR as people who were leading a grant related to dementia. GL wrote to all of those invited and most of them agreed to participate in the project as co-applicants or collaborators (and are co-authors of this review). She had also invited other academics with specific expertise in outcome measures or who were leading projects on outcome measures in dementia but not in this specific area. In addition, we asked people to the conference who had become involved during the project; LW who had been employed as research assistant for the project, an MSc student who had volunteered to work on the project (DG), the Alzheimer’s Society Research Engagement Officer who had led the day to day work on the PPI (AGS), the PPI representatives and a representative from Alzheimer’s Research UK who had expressed interest. A member of the original collaboration left academia (MB), and so put forward a colleague in her place (JTC).

The conference began with a summary of the project, including the background, aims and work streams of the project. GL, PI of the project, gave an overview of the purpose and workstreams of the project. LW presented an overview of the systematic review, including the methods, numbers screened and searched, and the main findings from this in terms of outcomes. The main feedback from the focus groups was presented by AGS. GL and RH chaired the meeting, and GL and LW took notes of discussions.

Champions with expertise within each of the domains synthesised the systematic review’s results and validation data to present recommendations for that domain at the conference (GM, SB, BW, JTO’B, RP, RH, and GL). They synthesised information about how often a measure was used, its psychometric data including coverage of the domain in question, its acceptability for participants and the time taken in administration, to make recommendations about which measures to use from those found in the review.

The conference attendees discussed the domain after each presentation and then agreed on recommendations for that domain. Once all domains had been discussed, there was further discussion as to how these recommendations would form the overall core outcome set. We made recommendations as to which domains were core and what measures should be used in these domains. We also made recommendations about measuring important, but non-core domains which may not change despite disease modification.

## Results

### Systematic review

We screened 22,918 original references and included 149, referring to 124 RCTs and one CCT (S1 Fig PRISMA diagram). They included 95 published trials, three published trial protocols, and 27 ongoing trials (their characteristics are listed in Tables A-C in S1 File). All tested pharmacological interventions. Most trials involved participants with AD, either alone (n = 111), or with AD and mild cognitive impairment (MCI; n = 8) and three vascular dementia (n = 3). Two trials included only participants with vascular dementia and one trial those with mild to moderate primary degenerative dementia or vascular dementia.

#### Outcomes

In total, 81 outcome measures were used across trials; 72 questionnaire/interview based measures and nine biological techniques to measure biomarkers. Two authors provided extra information regarding outcomes in response to our enquiries [15, 16]. Table 1 lists the outcome measures and numbers of trials in which they were used. They included:

- Cognition (measured across 117 trials by 31 outcome measures)
- Quality of Life (QoL; measured across 16 trials by 3 outcome measures)
- Activities of daily living (ADL; measured across 68 trials by 12 outcome measures)
- Neuropsychiatric symptoms (measured across 58 trials by 16 outcome measures)
- Global assessment (measured across 80 trials by 10 outcome measures)
- Biological markers (measured across 71 trials by 9 biological techniques)

**Table 1:** Overview of findings of the systematic review.

Findings (number of trials)
***Cognitive outcomes (117 trials measured at least one cognitive outcome; 31 different outcomes)*:**
*Global*: Alzheimer's Disease Assessment Scale—Cognitive Scale (92); Mattis Dementia Rating Scale (1); Mini Mental State Examination (83); Modified Telephone Interview for Cognitive Status (1); Vascular Dementia Assessment Scale cognitive subscale (1)
*Batteries*: CERAD Neuropsychological test battery (2); CogState Alzheimer’s Battery (6); Computerised Neuropsychological Test Battery (1); Frontal assessment battery (1); Mental Deterioration Battery (1); Neuropsychological Test Battery (7); Severe impairment battery (1); Syndrome-Short-Test (2); Wechsler Adult Intelligence Scale-Revised (1); Wechsler Memory Scale (5); Western aphasia battery (1)
*Individual tests (either used in combination or to supplement the ADAS-Cog or MMSE)*: Buschke Selective Reminding Test (3); Benton Visual Retention Test (1); Clock drawing task (2); Controlled Oral Word Association Test (2); Digit span test (2); Digit symbol (3); Dot Counting N-back task (1); Fluency tests (7); Mohs number cancellation test (1); Recall tasks (3); Rey memory test (1); Stroop Color Word Interference test (4); Token test (1); Trail making test (10); Word recognition (1)
***Techniques for biological markers (71 trials measured at least one biological marker; 9 different techniques)*:**
*Imaging*: EEG (3); Doppler ultrasound (1); MRI (30); MRS (1); PET (20); SPECT (1)
*Fluid*: Blood (35); CSF (48); Urine (1)
***Neuropsychiatric outcomes (58 trials measured at least one neuropsychiatric outcome; 16 different outcomes)*:**
*Global*: Alzheimer's Disease Assessment Scale—Non Cognitive Scale (7); Behavioural Pathology in Alzheimer's Disease Rating Scale (1); Brief Psychiatric Rating Scale (3); CERAD Behavioural Scale (1); Dysfunctional Behavior Rating Instrument (1); Neuropsychiatric inventory (38); Nurses observation scale for geriatric patients (2); Plutchik Geriatric Rating Scale (1); Revised Memory and Behavior Problems Checklist (1)
*Specific symptoms*: Cohen Mansfield Agitation Inventory (1); Columbia-suicide severity rating scale (3); Cornell Scale for depression in dementia (3); Geriatric Depression Scale (10); Hamilton Depression Rating Scale (5); Montgomery depression rating scale (2); Zerssen adjective mood scale (2)
***Quality of life outcomes (16 trials measured at least one QOL outcome; 3 different measures)*:**
DEMQOL (4); European Quality of Life–5 Dimensions Scale (5); Quality of Life in Alzheimer’s Disease Scale (8)
***Activities of daily living outcomes (68 trials measured at least one ADL outcome; 12 different measures)*:**
Alzheimer's Disease Co-operative Study—Activities of Daily Living (35); Alzheimer’s Disease Functional Assessment and Change Scale (2); Bristol Activities of Daily Living Scale (5); Dependence scale (2); Disability Assessment For Dementia (13); Functional Activities Questionnaire (3); Interview for deterioration in Daily Living Activities in Dementia (2); Katz Index of Activities of Daily Living Scale (4); Lawton Instrumental Activities of Daily Living Scale (8); Nuremberg Gerontopsychological Rating Scale for Activities of Daily Living (3); Physical self-maintenance scale (3); Video recorder home-behavioural assessment (1)
***Global outcomes (80 trials measured at least one global outcome; 10 different measures)*:**
*Impression of change*: Alzheimer’s Disease Cooperative Study—Clinical Global Impression of Change (8); Clinical Global Impression Scale (15); Clinician's Interview-Based Impression of Change Plus Caregiver Input (12)
*Multiple domains*: Blessed Dementia rating scale (3); Dementia Severity Rating Scale (3); Gottfries-Brane-Steen rating scale for dementia (4); Sandoz Clinical Assessment-Geriatric Scale (2); Short CAMDEX (1)
*Staging of dementia*: Clinical Dementia Rating (48); Global Deterioration Scale (6)

#### Outcome validation

The validation data for the outcomes in each domain is available in Tables D-I in S1 File. Due to the large number of cognitive outcomes we shortlisted five cognitive outcomes for which we would search for validation data (ADAS-Cog, Mini Mental State Examination [MMSE], CERAD Neuropsychological battery, the Neuropsychological battery and the CogState Alzheimer’s Battery), and excluded the other cognitive outcomes for a number of reasons. These were that they had only been used in one trial, they measure cognition in severe dementia or, they are not available in English, and they only refer to one domain. We also did not search for validation of cognitive tests measuring only one specific domain of cognition. As dementia is an impairment of more than one cognitive domain a global scale is more appropriate. For the neuropsychiatric outcomes we decided we would not recommend measures of only individual neuropsychiatric symptoms, such as agitation or depression, as they would add to the burden of a core outcome set therefore we did not search for validation data for these measures.

### Patient and public involvement

Eighteen people participated in the PPI exercise; four people with dementia, 13 family carers and one PPI group member. The main recommendations from the PPI were around improving the experience of participating in a trial in terms of timing, travel, engagement, language used and the role of an informant, as well as which outcome domains they thought were core.

#### Focus groups and first email consultation

Twelve people participated in face-to-face focus groups in London, Sheffield and Cambridge; including three people living with dementia, two current family carers, six former family carers, and one PPI group member. Some had participated in research before as a participant themselves, or supported a family member through participation; some had no research experience. Five of them (4 carers and 1 person with dementia) replied to the first email consultation after the groups about an overview of the findings from across all three focus groups.

The participants made general recommendations about what would help them to participate in research and complete outcomes, as well as recommendation specific to the outcome domains. At the start of each focus group session people were asked ‘what should be measured?’ Responses were varied but gave broad support to each of the six domains covered.

The recommendations around completing outcomes included the following.

#### Questionnaires content and delivery

Participants thought questions should be really clear as the wording of the questions affects people’s different answers. In addition, they thought that too many questions or fast delivery can make people with dementia anxious, and can seem like someone is *“getting at you” (carer)*

There were differing reactions to standardised questionnaires which we showed to the groups including people living with dementia. Some thought questions should emphasise the positives, that is what people can do rather than the skills people had lost but others disagreed The difference in opinions were due to some thinking that focusing on what someone cannot do was a stigmatising way of considering people with dementia, but others thought that it was probably relevant information.

“It’s more relevant what they cannot do than they can do, because there’s an awful lot more we can’t do than we can do” (Person living with dementia)

#### Time and travel

Participants at all three focus groups thought that both the time taken and travel required to complete outcome measures could be barriers to participation, and these aspect of the study should be well-thought-out, in the same way the scientific protocol would be. They stressed the importance of clearly setting out the time required, including waiting times and breaks, as carers felt that waiting time can be particularly difficult to explain to the person they support, and that breaks are even more necessary for people with dementia than for the general public.

“Where it’s got to the stage that you’re not aware that you’re not giving the right answer, or what you would have given at the beginning” (Person living with dementia)

Measures were deemed less acceptable if they required participants to travel to a specific centre each time, for example, biological techniques. Carers noted that being able to participate in research more locally would encourage and help with participation, regardless of whether measures included invasive biological techniques, due to the difficulties that travelling can bring to people who are physically unwell, as well as living with dementia.

“Going to have comorbidities… They’re going to have all the other things going on… so it makes it much more difficult” (carer).

#### Carers’ participation

Participants agreed on the *“importance of carer in the decision making”* for participating in research, but also discussed the likely disparity in answers according to who answers the questionnaires, whether it was people living with dementia or their carers, though they thought carers answers can provide additional data. They also highlighted that not all people with dementia will have a defined carer, or someone who is available to act as an informant.

“You can’t expect them to take days of work to come with you to an assessment… it’s difficult because then you’re relying on what I’m saying” (Person living with dementia)

#### Engagement

Many participants thought that clear communication during the study, including reminding them of what had already been discussed about the reasons for completing particular measures, would aid continued engagement in the trial. One participant also suggested that engagement may be enhanced if the particular measures used reflect the subtype of dementia, as they had experience of questions not reflecting the subtype of dementia and that can make the research seem *“a waste of time”*.

The recommendations specific to the outcome domains are the following.

#### Activities of daily living

Participants across the groups differed on opinions about the use of ADL measures as a core domain, but generally judged that instrumental ADLs were more relevant to mild to moderate dementia than basic ADLs.

“A lot of what’s down there now doesn’t apply to me” (Person living with dementia on items of an ADL scale that refer to basic ADLs)

Participants suggested that questions should ask why or how someone was impaired in these activities, as this was not included in ADL measures. For example, participants with dementia pointed out that they may still be able to do activities but in a different way to before they had dementia, such as by wearing clothes that are easier to put on or cooking simpler meals.

“Some task you can still do, just differently, just adapted” (Person living with dementia)

Additionally, people living with dementia suggested that they may avoid ADLs, such as using the telephone, due to lack of confidence rather than an inability to complete them, and that measures do not seem to take this into account.

“I can use the telephone, but I don’t have the confidence, I don’t like using the telephone” (Person living with dementia)

Carers also raised the point that some ADL questionnaires they have had to complete were very similar to disability benefits assessments, and that this may affect the answers that participants give.

“The link across into disability benefits… it’s suspicious” (carer)

#### Biological markers

Participants generally thought that biological markers should be a core domain, as they believed them to be the most reliable, objective measures of the disease process.

“Biological measures you’ve got something that everyone can agree on… you can compare like with like” (carer)

Carers had a number of general questions about the type and quality of data biological measures could provide including if one technique was more informative than another, what biological changes these measures detect, and how many people need to take part for any significant results.

“Do you get more information from a lumbar puncture than a scan?” (carer)

“Would you be looking for a reduction in amyloid or tau, or would you be looking for an arrest in the size of brain shrinkage?” (carer)

“How many people would you need for it to be significant?” (carer)

Overall, participants agreed that blood tests were acceptable and that it would not be difficult to commit to frequent tests, with the main barrier being travel.

“Weekly blood tests would be very happily tolerated” (carer).

Some volunteers particularly liked cerebral spinal fluid (CSF) measures even though they were aware of possible side-effects, but they thought misconceptions about what the procedure involves might discourage participation. Those who had experienced CSF tests, either directly or indirectly, did not mind them but found the need to have it done in a specific location, for example, a clinic, presented difficulties.

“A couple of hours and it’s done… it doesn’t put him off going the following year… he knows exactly what he’s let himself in for and he does it”

“I think the practicalities of it would be the bit that concerns me… practicalities should be made easier for the patient” (carer)

Most thought that imaging could be core measure as it could provide objective data, and that many people would consent to having scans, as giving biological data can make a person with dementia feel that they are contributing useful information.

“In going through that he is doing absolutely everything he can to further the research” (carer)

“It’s a positive action” (carer)

Practical issues around travel were raised, and volunteers agreed that scans may be difficult for some people with dementia, and thought lying still for 45–60 minutes would be impossible for some.

“Vascular and Alzheimer’s when it gets to the middle… you can’t keep people still… they don’t understand what’s happening” (carer)

“It bothers me about the scan… incredible noise being lashed down. I knew what was going on… but for people with dementia it could ruin them” (carer)

“different people react in different ways” (carer).

Having music or a screen to watch would improve the experience, giving the individual something to focus on; and that could be done for most participants “If there is some flexibility” within the MRI environment. Carers agreed that PET scanners were less restrictive than MRI, though the issue of having to keep still remained.

After the conference, participants were consulted about and agreed with the suggestion made there regarding the use of serial structural MRI in a select number of participants who had consented to the procedure, rather than including MRI as an essential component for every individual in the study design. They agreed that this could improve recruitment and retention, as the prospect of MRI scans can discourage participation.

#### Cognition

Overall, participants agreed that cognition should be a core measurement. However, volunteers felt that it should not be used in isolation as the only measure and suggested that cognitive scores needed contextual, qualitative information. Carers in particular suggested that more weight should be placed on cognition in the context of previous ability rather than cognition alone and were unaware that this was standard clinical practice.

“*It is more important to understand how a person’s cognitive impairments affect their activities of daily living and quality of life…than it is to rate their underlying cognitive skills*” *(carer)*

Participants, including both people living with dementia and carers, felt that memory tests can be demoralising. Carers particularly felt that it is distressing *“watching someone fail a test”*. People with dementia described the distress of seeing their score and performance worsen, and a tendency to try to prepare for tests to prevent this from happening.

Although they acknowledged its use as a standard cognitive measure, performed in a range of research and clinical contexts, several people did not like the MMSE. Some people with dementia thought it seemed irrelevant, as it gives a restricted account of dementia and carers described how they thought it would be difficult to have a cognitive measure that seemed relevant to all people with dementia. Some people preferred the ADAS-Cog to the MMSE as it is more detailed.

“Cognitive testing, because we are so different and the way it progresses in the different forms of dementia makes it so complicated to try and produce some kind of standardised measure” (carer)

#### Neuropsychiatric symptoms

Some participants said that behaviour is a core domain as changes in behaviour are often present in dementia, and may be more relevant than ADL, whilst others thought it was less applicable in the milder stages of dementia. In addition, they commented that questionnaires do not measure any reasons behind changes in behaviours or consider an individual’s personality. In particular, some felt that behaviour should be measured alongside cognition and understanding, which could affect whether someone becomes agitated or aggressive. Volunteers suggested that if behaviour were measured, it should, as it usually does, include sleep, agitation, wandering, repetitive behaviours, and changes in appetite.

“It’s teasing out the brain damage from actual personality traits” (carer)

“It’s about behavioural change over time” (carer)

#### Quality of life

Volunteers had different opinions over the inclusion of QoL measures as a core domain. One volunteer thought it was core, as it could give a summary of an individual’s experience of dementia. Others were unsure about the sensitivity of QoL measures. In particular, some felt that QoL measures lack detail regarding the individual’s personality. For example one carer mentioned that someone may have always have disliked social events, and that a questionnaire may not pick this up. It was suggested that comparing carer and patient responses would give the most accurate account of QoL, as carers and patients may have different responses, and they thought a carer’s interpretation alone was not necessarily accurate.

“I don’t know how much you’d get out of it”

#### Global

Volunteers had differing opinions about global rating scales, with some liking the breadth of the measures, while others suggested that global measures were superficial and depend too much on the day in which it is measured, and therefore were not meaningful. Those who did not like global scores suggested that a larger package of specific measures would give a holistic view of an individual and include more detail.

“I like the global one… it’s all encompassing” (carer)

“You might be feeling particularly bad that day” (carer)

“Is that really valuable… Am I giving my time for something that’s meaningful? I’m not really convinced” (carer)

### Consensus conference

The conference presentations are available in S2 File. Twenty-seven people attended from a wide range of specialities within dementia research including:

- Alzheimer’s RUK–Alison Evans
- Alzheimer’s Society—Anna Grinbergs-Saull, James Pickett Applied psychosocial dementia research/ occupational therapy—Gail Mountain
- Dementia care—Frances Bunn, Claire Goodman
- Health service research—Sasha Shepperd
- Health outcome measurement—Sallie Lamb, Charlotte Roberts
- Old age medicine—Roy Jones
- Old age psychiatry—Sube Banerjee, Chris Fox, Rob Howard, Gill Livingston, John T O’Brien, Robert Perneczky
- Psychology and dementia—Georgina Charlesworth, Esme Moniz-Cook, Bob Woods
- Public health and ageing—Louise Lafortune
- Social care and social policy—David Challis, Katie Featherstone, Justine Schneider, Claire Surr
- Systematic reviews—Jo Thompson-Coon

And the two researchers who conducted the systematic review; Derek Groskreutz, and Lucy Webster.

Not all of those who were invited to the conference were able to attend.

#### Cognition

It was agreed that cognition is a core domain for disease modification trials, and that a measure should be reliable and sensitive in showing decline, which both the ADAS-Cog and MMSE have been shown to do in the earlier cholinesterase inhibitor trials [17], as well as being the most commonly used and validated outcomes. Due to this, both of the measures were decided to be the only real contenders from the cognitive measures used across the studies found in the review. The PPI consultation emphasised that patients dislike failing in tests of cognition and at the conference it was discussed that to improve the experience of cognitive tests in the future timed measures which estimate cognitive processing speed should be considered, as they do not mean the patient feels they have passed or failed individual items, though these would need validating.

There was debate around the ADAS-Cog or MMSE, as the ADAS-Cog is longer and therefore more frequently has missing data, but may be more sensitive to change so it might need fewer participants than a trial powered on the MMSE. Due to the ADAS-Cog having a larger number of items, this may give the impression of potential greater sensitivity to change than the MMSE, but superiority was not seen in the trials of symptomatic treatments [17]. The MMSE is affected by education, and also lacks sensitivity in differentiating between very early AD or MCI and normal ageing [18, 19]. However, in mild to moderate AD it has reasonable psychometric properties and was sensitive to change in earlier cholinesterase and memantine trials [20]. Further, because the ADAS-Cog is not used in clinical practice, clinicians do not have a “feel” for what difference in score would constitute a meaningful change. The ADAS-Cog has also been criticised for not sufficiently assessing attention, planning, working memory and executive functioning, all of which are impaired at the earliest stages of AD, which led to the addition of extra tests to the original version [21]. These shortcomings also apply to the MMSE [22]. Overall for cognition we recommend the MMSE or ADAS-Cog, as they are the best available measures of those found in our review and considered by our consensus panel. Both of the cognitive outcomes chosen have minimum clinical important difference (MCID) available. The MCID in the MMSE is 1.4 points [23]. MCID, for the ADAS-Cog has been calculated as 3 or 4 for early Alzheimer’s disease [24, 25]

#### Fluid makers

The use of CSF biomarkers was discussed. Our systematic review confirms that the three established markers Aβ42, tTau and pTau181 are also the most widely used markers in AD dementia clinical trials. Even though AD fluid markers are useful in order to enrich studies with true AD cases, and to measure target engagement, their usefulness as outcome measures is limited based on the available evidence. A meta-analysis showed changes in putative biomarkers of Aβ42 are not related to change in MMSE so they do not appear to be helpful biomarkers for disease modification [26]. In addition, this technique can be expensive due to potential medical care needed after [27]. It was therefore agreed not to recommend a fluid biomarker and that currently the measurement of CSF biomarkers is not a core outcome in disease modification trials.

#### Imaging

Imaging is important for disease modification, though changes in imaging and cognition are not always highly correlated; between group differences may show mechanism of change as well as disease modification [28, 29]. There were only three potential outcome measures for imaging with sufficient evidence of sensitivity to consider using them as outcomes. These were serial structural MRI, serial amyloid Positron Emission Tomography (PET) and serial Fludeoxyglucose PET. Volumetric MRI does not show expected changes in terms of atrophy immediately if amyloid is removed, as the volume may go down in treated patients. It however shows brain changes over time. Thus overall there is usually a decrease of 2% over a year in AD and 0.5% in DLB [30]. Structural MRI was the best imaging technique to find sensitivity to change in untreated patients, is validated against underlying pathology, and is available in UK sites for use as measure [29, 31]. In addition, it is cheaper with the NHS tariff being £110 compared to an approximate cost of FDG PET is around £1000 and amyloid PET being £1600.

Earlier trials have found that they may need fewer participants than for cognitive changes to be fully powered, [32–34] meaning MRIs can therefore be conducted in a subgroup of participants in disease modification trials. This means that participants who were unable, or did not wish, to have an MRI would still be able to take part in trials.

Of the biological techniques found in our review, imaging was judged to be a core domain by the consensus panel. The evidence was that serial structural MRI was the best technique currently and we are recommending using an optional serial structural MRI in a voluntary subgroup of participants. As there is rapid innovation of biomarkers, this recommendation may change in the future.

#### Neuropsychiatric symptoms

There was debate around whether neuropsychiatric symptoms should be a core domain, as not everyone with dementia, especially in the milder stages will have these symptoms, and if they do they may not be clinically significant, meaning that even if a treatment is disease modifying there may be no difference in these symptoms [35, 36]. Some members of the group thought neuropsychiatric symptoms were core, as a disease modifying treatment should improve behaviour, or at least not make it worse but overall the conference judged this to be important but not a core outcome for every future study of disease modification as many people with mild dementia do not have any neuropsychiatric symptoms. If neuropsychiatric symptoms are measured we recommend that studies use the Neuropsychiatric Inventory (NPI), as it is the best of the available neuropsychiatric measures that our panel considered as the only measure which includes both frequency and severity of symptoms and has appropriate psychometric properties (Tables D-I in S1 File).

#### Activities of daily living

It was discussed that activities of daily living should be measured ideally through a proxy as impairment may be underestimated by people living with dementia, though it is not always [37]. Some scales try to differentiate between the performance and initiation of ADLs, and thus ask whether people with dementia need prompting to complete ADLs. This domain is being used less in new disease modification trials as it is affected by physical health and thus any changes may be unrelated to dementia. In addition, the measures may not have sensitivity to change in mild dementia as there may be very little impairment. Finally, the measures do not take into account that people come from a range of backgrounds and some may never have engaged in tasks such as cooking [38]. Overall it was therefore decided that ADL it is not a core measure of activity limitation. If one is used in disease modification trials, we recommend the DAD as it is the best available dementia specific measure of ADL from the 12 measures the consensus panel had to consider (Tables D-I in S1 File).

#### Quality of life

Quality of life was discussed and it was considered to be different to other domains as people with severe dementia can have good life quality [39–41], and therefore it is not a sensitive outcome to measure disease modification. However, it is useful to understand negative or positive impacts of pharmacological interventions. As the self-reporting of QoL becomes more difficult over time, it is ideal to get a proxy report too so that if the participant deteriorates and is less able to give information reports can be compared from earlier in the trial. It was also discussed that quality of life is important in phase three trials where economics need to be measured for cost by a dementia QALY (Quality-adjusted life years). The conference did not include economists so did not have the required expertise to discuss or make recommendations about QALYs. If quality of life is measured, the conference recommended the DEMQOL, which is able to measure quality of life from both the person with dementia and proxy perspectives, and therefore considered the best measure of the three found in our review. We also note that QALY values can be calculated from it.

#### Global functioning

The group considered that global functioning was an important, but not core, measure as it encompasses several domains. If it was used they recommended either a staging or impression of change outcome measure, rather than a multi domain scale. A staging instrument was generally thought to be best by the consensus panel as it is less subjective than an impression of change. It was decided not to recommend a global instrument as core, but if it was used that the Clinical Dementia Rating global score CDR would be the instrument of choice from the ten measures considered by the panel. It has good psychometric properties and that the global score may be more appropriate than the sum of boxes so that particular domains were not prioritised or contributed disproportionately to the overall score.

#### Main findings

The attendees decided that both cognition (as the primary symptom in dementia) and biological markers (measuring disease modification) were the core outcomes that should be measured in all disease modification trials in mild-to-moderate dementia. They recommended that cognition should be measured using the Mini Mental State Examination (MMSE) [42] or Alzheimer’s Disease Assessment Scale–Cognitive Subscale (ADAS-Cog) [43], and as a biological marker serial structural MRI should be used as a measure for the subset of participants who consent to have it.

The other domains covered potentially important additional effects in disease modification treatments but were not assessed as core, in that dementia could be modified without necessarily improving them. These were: ADL, neuropsychiatric symptoms, QoL, and global ratings. All were deemed important although not essential and it was concluded that they should be used dependent on study goals and to consider effects of interventions other than disease modification. The conference recommended the Disability Assessment for Dementia (DAD) [44] for ADL, the Neuropsychiatric Inventory (NPI) [45] for neuropsychiatric symptoms, DEMQOL [46] for quality of life, and the CDR [47] for global ratings.

We also discussed the issue of informant rated scales, and how it was necessary that the informant had seen the person frequently in the weeks prior to completing the scale, so the availability of an informant or supporter or each participant is part of the study design.

#### Second email consultation

After the core recommendations from the conference, we conducted a second electronic consultation with the AS research network. We received 6 responses from people with experience of dementia; one person with dementia, and five carers (three current carers and two former carers). Those who answered this additional consultation agreed with the main recommendations from the conference.

## Discussion

We found that trials to date for disease modification in mild to moderate dementia had used 81 different outcome measures. This demonstrates the necessity of gaining a consensus about a core set of the best available measures in order to harmonise future disease modification trials. The UK dementia research community has engaged in the task of agreeing core outcomes to help identify treatments. Our consensus process identified two domains as core. These were cognition, measured via the MMSE [42] or ADAS-Cog [43]; and biological markers to be measured through serial structural MRI. We recommend that those who are unable or unwilling to have an MRI should still be able to participate in the trial. We also made outcome recommendations for the important, but non-core, domains of activities of daily living, global, neuropsychiatric and quality of life; respectively recommending the DAD [44], CDR [47], NPI [45], and DEMQOL [46].

One problem for disease modification trials is that outcomes need to be very sensitive in the early stages of dementia, when subtle and slow changes occur; these cognitive measures have shown this sensitivity [48]. Though outcomes, such as cognition, give an indication of clinical benefit, they do not by themselves, demonstrate whether an intervention has disease modifying properties, whereas a biomarker could [49]. Additional biological outcome markers may be appropriate and would be selected depending on the proposed mechanism of action of the therapy. They might be applied only to a subset of participants.

We reached our conclusions and recommendations through consensus conference discussion with expert researchers and clinicians. We used instruments already in use by the research community and therefore found to be acceptable to at least some groups. We had a very large number of collaborators and gained consensus. We think it is also a strength and that our findings may gain traction and influence future research because our project was a response to the UK NIHR’s call who will recommend the findings inform the design of future studies [10].

e balanced the psychometric properties and the areas covered by the outcomes found within the systematic review and were informed by input from the voluntary sector and patients and families. This included consideration of the practicalities of the completion of the core outcome set, including timing, breaks, travel, and whether the person with dementia or an informant completes the measures. We discussed the issue of informant rated scales, and how the informant needs to have seen the person regularly in the weeks prior to completing the scale, underlining the necessity of having an informant for each participant.

Though there have been previous discussions about how and which outcomes should be measured, in disease modification trials in mild to moderate dementia [50–53], there was no core outcome set identified for this type of trial. Inherent in our method was the assumption that the ‘optimal’ outcome assessment has already been used in a disease modification RCT and this may not be the case. As knowledge progresses the instruments used to measure, but not which domains to include, may change. The recommended measures are currently the best available of those found in our systematic review and considered by our consensus panel, but we expect them to be superseded, as there are limitations with the measures we have recommended. For example, as the ADAS-Cog can only be administered by a trained tester and takes 45 minutes to complete, it may be seem more practical to use the MMSE, which can be administered by clinical staff with minimal extra training and takes 10 minutes to complete. However, due to copyright the MMSE can be costly to use [54]. The MMSE can also be affected by level of education, though the memory domain of the measure seems to be less affected than the other domains [19].

Furthermore, change in current biomarkers of AD in particular does not always translate to changes in disease, and the improvement of current and development of new biomarkers are key challenges in working towards a disease modifying treatment, particularly with the development of adaptive trial designs [8]. Sensitive biomarkers could enable trials of potential disease modifying interventions to be shorter as they would be able to detect changes in disease progression; however the development and validation of biomarkers takes time [48]. This is a hugely innovative field, meaning it may be important to consider other biomarkers as core outcomes in the future if they are objective and reliable enough to show a potential disease modifying effect. Ultimately the choice of a biological marker depends on the resources available and in a pharmacological study, on the compound being tested.

It is important to consider participants’ willingness to participate in collecting biomarkers in disease modification trials. Participants in the PPI consultation were generally enthusiastic about it, despite biological tests being invasive but also highlighted how these may dissuade potential participants due to side effects, travel required, misconceptions and anxiety. They supported our recommendation of an MRI as an optional outcome rather than essential for individuals, and noted that this would make them more willing to take part in a trial. As biomarkers develop, the side effects of the biological techniques used (such as lumbar puncture) will also need to be considered, particularly with CSF, where 30% of people in memory clinics report side effects, although these are rarely serious or long lasting [55].

We are unable to make recommendations for different types of dementia, such as vascular or frontotemporal, as most of the trials reviewed included only participants with AD. This is potentially a limitation for outcome measurement in disease modification trials not focusing on participants with AD. More work is needed to address disease modification of the less common types of dementia [56]. It is outside the remit of this study to make recommendations for disease modification trials focusing on mild cognitive impairment or consider the economic outcomes. Finally our PPI was very small scale, all participants came from the Alzheimer’s Society and so we may not have covered the spectrum of opinion about participation in trials, and we did not conduct a full in-depth qualitative study or analysis. We conducted brief searches for the psychometric properties of outcomes using one search engine, which may limit the results of the outcome validation. While our systematic review was international, the authors of this paper all currently work in the UK which may limit the generalisability of the results. They have, however, worked in many different countries and also come from a variety of backgrounds and nations. We recognise that disease can be conceptualised in a variety of fashions but pragmatically we chose to discriminate between measures as core or not by whether dementia could be modified without improving them. We of course acknowledge the complexity of the web of causation [57]. We also made recommendations for other important measures which are not essential to measure disease modification.

Despite these limitations, this study represents a robust approach to define a core and non-core set of outcomes and measures for use across disease modifying trials in Alzheimer’s disease. It is the first study to conduct a large-scale systematic review on which outcomes have been used in published and ongoing disease modification trials, and gather data on these outcomes to inform expert consensus group recommendations. We included ongoing trials to understand what outcomes are currently being used, though there was, for some of these trials, less information available about the outcomes used on trial registries than in comparison to trials published in journal articles.

We have added to our search by drawing on research from members of our group around the development of core outcome sets for dementia from perspectives other than disease modification, including a standard set of outcomes of what matters the most to people living with dementia [13], and a core outcome set to be used in psychosocial intervention trials of dementia [11, 12].

With regard to future research, as we recommend either the ADAS-Cog [43] or MMSE [42], which are the most commonly used cognitive outcomes for disease modification trials; it would be useful to develop an algorithm to allow direct comparison of both scores. As informants often rate non-core measures, it would also be useful to standardise the definition of an informant.

work. This does not alter our adherence to PLOS ONE policies on sharing data and materials

## Supporting information

S1 Fig PRISMA flow diagram of the results from the systematic review. (DOCX)

S1 File Results from the systematic review and validation of outcomes. Tables A-C of study characteristics of included published studies, published protocols, and ongoing trials. Tables D-I of validation of outcomes for the seven outcome domains.
(DOCX)

S2 File Presentations from the consensus conference. (PDF)
