# Supplementary material for: Core outcome measures for interventions to prevent or slow the progress of dementia for people living with mild to moderate dementia: Systematic review and consensus recommendations
Source: PLoS One. 2017 Jun 29;12(6):e0179521. doi: 10.1371/journal.pone.0179521 (PMC5491018; doi:10.1371/journal.pone.0179521)
Supplement: S1 Fig — (DOCX) [file pone.0179521.s001.docx]

**Studies included:**
(n = 149 reports, including 125 trials)

**S1 Fig. PRISMA flow diagram of the results from the systematic review**

**Records identified through sources in workstream 1**

ALOIS (n = 167)
Systematic review of non-pharmacological interventions (n = 167)

**Records identified through hand searching:**

(n = 39)

**Records after duplicates removed:**
(n = 22,918)

**Records excluded:**
(n = 22,021)

**Records screened**:
(n = 22,918)

**Full-text articles assessed for eligibility:**
(n = 897)

**Full-text articles excluded, with reasons:**
(n = 748)

- Unable to find a copy of the full text (n = 4)
- Full text unavailable in English (n = 30)
- Not published in a peer reviewed journal or an ongoing trial (n = 150)
- No participants with mild or moderate dementia (n = 5)
- Not disease modification trial (n = 550)
- Not an RCT or CCT (n = 5)
- No quantitative outcome relating to disease modification (n = 4)

**Records identified through sources in workstream 2**

CENTRAL (n = 5689)

MEDLINE (n = 8509)

PsycINFO (n = 2973)

EMBASE (n = 16,426)

LILACS (n = 203)

CINAHL (n = 3085)

ClinicalTrials.gov (n = 493)

ISRCTN (n = 36)
