# Supplementary material for: Core outcome measures for interventions to prevent or slow the progress of dementia for people living with mild to moderate dementia: Systematic review and consensus recommendations
Source: PLoS One. 2017 Jun 29;12(6):e0179521. doi: 10.1371/journal.pone.0179521 (PMC5491018; doi:10.1371/journal.pone.0179521)
Supplement: S1 File — Tables A-C of study characteristics of included published studies, published protocols, and ongoing trials. Tables D-I of validation of outcomes for the seven outcome domains. (DOCX) [file pone.0179521.s002.docx]

# Appendix A MEDLINE (OvidSP) Search strategy

1. exp Alzheimer Disease/
2. exp Dementia, Vascular/
3. exp "Pick Disease of the Brain"/
4. exp Dementia, Multi-Infarct/
5. exp Cognition Disorders/ or exp Dementia/
6. dement*.mp.
7. alzheimer*.mp.
8. (lewy* and dement*).af.
9. multiple infarcts.mp.
10. exp Supranuclear Palsy, Progressive/
11. (pick adj5 disease).mp.
12. "Frontotemporal Dementia".mp. or exp Frontotemporal Dementia/
13. (park* and dement*).af.
14. 1 or 2 or 3 or 4 or 5 or 6 or 7 or 8 or 9 or 10 or 11 or 12 or 13
15. exp "Outcome Assessment (Health Care)"/
16. “outcome”.mp.
17. “instrument”.mp.
18. “measure”.mp.
19. outcome*.mp.
20. instrument*.mp.
21. measure*.mp.
22. 16 or 17 or 18 or 19 or 20 or 21
23. intervention.mp.
24. therap*.mp.
25. trial*.mp.
26. 23 or 24 or 25
27. control*.mp.
28. 22 or 26 or 27
29. limit 28 to english language

**TABLE A** Published trials

| **Author and year** | **Trial location** | **Trial type** | **Dementia type and severity** | **Criteria for dementia diagnosis** | **Total participants** | **Description of intervention (n)** | **Description of control/ comparator group (n)** | **Participants sex (% of females)** | **Participants mean age (range)** | **Outcomes related to disease modification** |
| --- | --- | --- | --- | --- | --- | --- | --- | --- | --- | --- |
| Aisen, 2000/ Aisen et al., 2000[1,2] | USA | RCT | mild to moderate AD | MMSE score 13-26 | 138 | prednisone 20mg/d for 4 weeks, then 10mg/d for 1 year (69) | placebo (69) | prednisone 49.3%, placebo 50.7% | prednisone 73.4, placebo 72.3 | *Primary:* |
|  |  |  |  |  |  |  |  |  |  | ADAS-Cog |
|  |  |  |  |  |  |  |  |  |  | *Secondary:* |
|  |  |  |  |  |  |  |  |  |  | CDR (SB); BDRS; HAM-D; BPRS |
| Aisen et al., 2002[3] | USA | RCT | mild to moderate AD | NINCDS-ADRDA | 40 | nimesulide 200mg/d for 12 weeks (21) | placebo (19) | nimesulide 38%, placebo 47% | nimesulide 73, placebo 74 | ADAS-Cog; MMSE; CDR (SB); BPRS; HAM-D; BDRS (ADL section) |
| Aisen et al., 2003[4] | USA | RCT | mild to moderate AD | MMSE score 13-26 | 351 | rofecoxib 25 mg/d (122) or naproxen 440mg/d (118) for 12 months | placebo (111) | placebo 55.9%, naproxen 48.3%, rofecoxib 54.9% | placebo 73.8, naproxen 74.1, rofecoxib 73.7 | *Primary:* |
|  |  |  |  |  |  |  |  |  |  | ADAS-Cog |
|  |  |  |  |  |  |  |  |  |  | *Secondary:* |
|  |  |  |  |  |  |  |  |  |  | CDR (SB); NPI; QOL-AD; ADCS-ADL |
| Aisen et al., 2006[5] | USA | RCT | mild to moderate AD | DSM-4 and MMSE score 13-25 | 58 | 3APS 100mg/d (15), 200mg/d (16) or 300mg/d (14) for 3 months | placebo (13) | 100mg 33%, 200mg 75%, 300mg 50%, placebo 46% | 75.1 | CSF (Aβ40, Aβ42, and t-tau); ADAS-Cog; MMSE; CDR (SB) |
| Aisen et al., 2007[6] | USA | RCT | mild to moderate AD | MMSE score 13-25 | 58 | alzhemed 100mg/d, 200mg/d or 300mg/d for 3 months (n not specified) | placebo (n not specified) | not specified | not specified | *Primary:* |
|  |  |  |  |  |  |  |  |  |  | MMSE; ADAS-Cog; CDR (SB) |
|  |  |  |  |  |  |  |  |  |  | *Secondary:* |
|  |  |  |  |  |  |  |  |  |  | CSF (biomarkers: only Aβ42 mentioned) |
| Aisen et al., 2008/ National Institute on Aging, and the General Clinical Research Center Programme, 2008/ Viswanathan, 2009[7-9] | USA | RCT | mild to moderate AD | MMSE score 14-26 | 409 | folic acid 5mg/d, vitamin B12 1mg/d, and vitamin B6 25mg/d for 18 months (240) | placebo (169) | 56% | 76.3 (50+) | *Primary:* |
|  |  |  |  |  |  |  |  |  |  | ADAS-Cog |
|  |  |  |  |  |  |  |  |  |  | *Secondary:* |
|  |  |  |  |  |  |  |  |  |  | MMSE; CDR (SB); ADCS-ADL; NPI; QOL-AD; Blood (Homocysteine Levels) |
| Aisen et al., 2011/ Gauthier et al., 2009/ Saumier et al., 2009[10-12] | Canada and USA | RCT | mild to moderate AD | DSM-4, NINCDS-ADRDA, and MMSE score 16-26, | 1052 | tramiprosate 200 mg/d (352) or tramiprosate 300 mg/d (347) for 78 weeks | placebo (353) | 53% | - 1. (48-94) | ADAS-Cog; CDR (SB); MMSE; CIBIC+; NPI; DAD; Blood (Aβ); CSF (tau and Aβ); Urine (Aβ) |
|  |  |  |  |  |  |  |  |  |  | *In substudy:* |
|  |  |  |  |  |  |  |  |  |  | MRI (n = 312; volumetric) |
| Akhondzadeh et al., 2010[13] | Iran | RCT | mild to moderate AD | DSM-4, NINCDS-ADRDA and MMSE score 15-26 | 54 | saffron 30mg/d for 22 weeks (27) | donepezil 10mg/d (27) | saffron 48%, donepezil 44% | saffron 72.7, donepezil 73.85 (55+) | MMSE; ADAS-Cog; CDR (SB) |
| Akhondzadeh et al., 2010[14] | Iran | RCT | mild to moderate AD | DSM-4 and MMSE score 15-26 | 46 | saffron 30mg/d for 16 weeks (23) | placebo (23) | saffron 43%, placebo 48% | saffron 72.65, placebo 73.13 (55+) | MMSE; ADAS-Cog; CDR (SB) |
| Alvarez et al., 2000[15] | Spain | RCT | mild to moderate AD or vascular dementia | NINCDS-ADRDA, DSM-4, and MMSE score 14-26 | 45 | anapsos 360mg/d (15) or 720mg/d (15) for 4 weeks | placebo (15) | not specified | 73.8 (56-89) | *Primary:* |
|  |  |  |  |  |  |  |  |  |  | ADAS-Cog |
|  |  |  |  |  |  |  |  |  |  | *Secondary:* |
|  |  |  |  |  |  |  |  |  |  | EEG; Doppler ultrasound (blood flow hemodynamics) |
| Alvarez et al., 2006/ Alvarez et al., 2011[16,17] | Spain | RCT | mild to moderate AD | NINCDS-ADRDA, DSM-4, and MMSE score 14-25 | 279 | cerebrolysin 10ml/d (69), 30ml/d (70), or 60ml/d (71) 5 days per week for 4 weeks and on 2 days per week for next 8 weeks | placebo (69) | 10ml 71.7%, 30ml 75.4%, 60ml 70.6%, placebo 65.5% | 10ml 72.2, 30ml 73.4, 60ml 74.6, placebo 73.9 | *Primary:* |
|  |  |  |  |  |  |  |  |  |  | ADAS-Cog; CIBIC+ |
|  |  |  |  |  |  |  |  |  |  | *Secondary:* |
|  |  |  |  |  |  |  |  |  |  | MMSE; NPI; Trail making test; DAD |
| Asthana et al., 1999[18] | USA | RCT | mild to moderate AD | NINCDS-ADRDA | 12 | 0.05 mg/d of 17 b-estradiol for 8 weeks (6) | placebo (6) | 100% | b-estrdiol 79.5 (66–89), placebo 77.6 (70–86) | Buschke Selective Reminding Test; Wechsler Memory Scale; Stroop Color Word Interference test; Trail making test; Fluency test; Token test; MMSE; BDRS (cognitive section); BPRS; Blood (IGF-1 and IGFBP-3) |
| Babiloni et al., 2009/ Pasqualetti et al., 2009[19,20] | Italy and USA | RCT | mild to moderate AD | NINCDS-ADRDA and MMSE score 16-25 | 132 | 800mg/d ibuprofen (and 20mg/d esomeprazol) for 12 months (66) | placebo (66) | placebo 65%, ibuprofen 61% | placebo 74.0, ibuprofen 73.7 | *Primary:* |
|  |  |  |  |  |  |  |  |  |  | ADAS-Cog |
|  |  |  |  |  |  |  |  |  |  | *Secondary:* |
|  |  |  |  |  |  |  |  |  |  | MMSE; Geriatric DS; Katz ADL; Lawton IADL; NPI; CDR (SB and global); CIBIC+ |
| Bae et al., 2000[21] | Korea | RCT | mild to moderate AD | NINCDS-ADRDA and MMSE score 10-24 | 53 | cerebrolysin 30ml/d for 4 weeks (34) | placebo (19) | cerebrolysin 68%, placebo 63% | cerebrolysin 73.1, placebo 69.0 | *Primary:* |
|  |  |  |  |  |  |  |  |  |  | ADAS-Cog; CGI |
|  |  |  |  |  |  |  |  |  |  | *Secondary:* |
|  |  |  |  |  |  |  |  |  |  | MMSE; Geriatric DS; Katz ADL; Lawton IADL |
| Ban et al., 1990[22] | Italy | RCT | mild to moderate primary degenerate dementia or vascular dementia | DSM-3 | 178 | nimodipine 90mg/d for 12 weeks (89) | placebo (89) | nimodipine 55%, placebo 61% | - 1. (55-95) | CGI; HAM-D; MMSE; Global DS; SCAG; PGRS; Wechsler Memory Scale; Blood (serum bilirubin, alkaline phosphatase, lactic dehydrogenase, electrolytes (Na, K, Cl), cholesterol, total cop, SGOT, SGPT and BUN determinations) |
| Bayer et al., 2005/ Holmes et al., 2008[23,24] | UK | RCT | mild to moderate AD | NINCDS-ADRDA and MMSE score 14-26 | 80 | immunisation AN1792 (50 μg or 225μg) with QS-21 adjuvant (50μg or 100μg) for 24 weeks (16 in each group) | QS-21 50 (8), QS-21 100 (8) | QS-21 50μg 25% QS-21 100μg 25% AN1792 50μg + QS-21 50μg 37.5%, AN1792 50μg + QS-21 100μg 43.8%, AN1792 225μg + QS-21 50μg 43.8%, AN1792 225μg + QS-21 100μg 31.3% | QS-21 50μg 70.3, QS-21 100μg 72.5, AN1792 50μg + QS-21 50μg 74.3, AN1792 50μg + QS-21 100μg 74.1, AN1792 225μg + QS-21 50μg 72.3, AN1792 225μg + QS-21 100μg 71.7 (under 85) | *Primary:* |
|  |  |  |  |  |  |  |  |  |  | ADAS-Cog; MMSE; ADCS-CGIC; DAD |
| Bentham et al., 2008[25] | UK | RCT | mild to moderate AD, with or without vascular dementia | DSM-4 | 310 | aspirin 75mg/d for 12 weeks (156) | avoid aspirin (154) | aspirin 63%, non-aspirin 62% | aspirin (51–90), non-aspirin (46–90) | *Primary:* |
|  |  |  |  |  |  |  |  |  |  | MMSE; Bristol ADL |
|  |  |  |  |  |  |  |  |  |  | *Secondary:* |
|  |  |  |  |  |  |  |  |  |  | NPI |
| Bilikiewicz, 2004[26] | Poland | RCT | mild to moderate AD | DSM-4, NINCDS-ADRDA, and MMSE score 10-24 | 105 | colostrinin was 100μg on alternate days for three weeks followed by two weeks drug-free -cycle repeated three times for 15 weeks (53) | placebo (52) | not specified | (50+) | *Primary:* |
|  |  |  |  |  |  |  |  |  |  | ADAS-Cog; CGI |
|  |  |  |  |  |  |  |  |  |  | *Secondary:* |
|  |  |  |  |  |  |  |  |  |  | Lawton IADL; MMSE; Global DS; Geriatric DS; ADAS-Noncog |
| Black et al., 2010[27] | USA | RCT | mild to moderate AD | NINCDS-ADRDA and MMSE score 14-26 | 30 | intravenous bapineuzumab 0.5mg/kg (6), 1.0mg/kg (6), or 5.0mg/kg (10) every 10 weeks for 52 weeks | placebo (8) | placebo 87.5%, 0.5mg 50%, 1.5mg 16.67%, 5mg 30% | placebo 69.88, 0.5mg 74.67, 1.5mg 72.33, 5mg 74.70 (50-85) | *Primary:* |
|  |  |  |  |  |  |  |  |  |  | MMSE |
|  |  |  |  |  |  |  |  |  |  | *Secondary:* |
|  |  |  |  |  |  |  |  |  |  | Blood (Aβ1 and Aβ40) |
| Blennow et al., 2012/ Rinne et al., 2010[28,29] | Finland and UK | RCT | mild to moderate AD | NINCDS-ADRDA and MMSE score 18−26 | 28 | intravenous bapineuzumab 0.5mg/kg (7), 1.0mg/kg (7), or 2.0mg/kg (6) every 13 weeks up to 78 weeks | placebo (8) | bapineuzumab groups 42%, placebo 57% | bapineuzumab groups 67.26, placebo 70.00 (50-80) | *Primary:* |
|  |  |  |  |  |  |  |  |  |  | ADAS-Cog; DAD; Neuropsychological test battery (NTB); MMSE; PET (amyloid and glucose) |
|  |  |  |  |  |  |  |  |  |  | *Secondary:* |
|  |  |  |  |  |  |  |  |  |  | CDR (SB); NPI; MRI (volumetric) |
|  |  |  |  |  |  |  |  |  |  | *In substudy:* |
|  |  |  |  |  |  |  |  |  |  | CSF (total tau, phosphorylated tau, and Aβ) |
| Blennow et al., 2012/ Salloway et al., 2009[29,30] | USA | RCT | mild to moderate AD | MMSE score 16-26 | 229 | intravenous bapineuzumab in four ascending dose groups 0.15mg/kg, 0.5mg/kg, 1.0mg/kg, or 2.0mg/kg every 13 weeks up to 78 weeks (124 between all 4 groups) | placebo (110) | bapineuzumab 50.0%, placebo 59.8% | 69.1 | *Primary:* |
|  |  |  |  |  |  |  |  |  |  | ADAS-Cog; DAD |
|  |  |  |  |  |  |  |  |  |  | *Secondary:* |
|  |  |  |  |  |  |  |  |  |  | Neuropsychological test battery (NTB); MMSE; CDR (SB); MRI (volumetric) |
|  |  |  |  |  |  |  |  |  |  | *In substudy:* |
|  |  |  |  |  |  |  |  |  |  | CSF (n = 35; total tau, phosphorylated-tau, and Aβ42) |
| Bowen et al., 2015[31] | USA | RCT | mild to moderate AD | NINCDS-ADRDA and MMSE score 12-24 | 108 | lupron depot 11.25mg (36) or 22.5mg (36) every 12 weeks for 48 weeks | placebo depot (36) | 100% | 11.25mg 78.75 (67-93), 22.5mg 78.25 (67-88), placebo 76.97 (65-88) | *Primary:* |
|  |  |  |  |  |  |  |  |  |  | ADAS-Cog; ADCS-CGIC |
|  |  |  |  |  |  |  |  |  |  | *Secondary:* |
|  |  |  |  |  |  |  |  |  |  | NPI; HAM-D; ADCS-ADL |
| Claxton et al., 2015[32] | USA | RCT | amnestic MCI (n=39) or mild to moderate AD (n=21) | NINCDS-ADRDA and MMSE score >15 | 60 | 20IU/d insulin (20) or 40IU/d insulin (20) for 21 days | placebo (20) | not specified | not specified | *Primary:* |
|  |  |  |  |  |  |  |  |  |  | Recall tasks; Buschke Selective Reminding Test |
|  |  |  |  |  |  |  |  |  |  | *Secondary:* |
|  |  |  |  |  |  |  |  |  |  | DSRS; BVRT; Dot Counting N-back task; Stroop Color-Word Interference task |
| Craft et al., 2012[33] | USA | RCT | amnestic MCI (n=64) or mild to moderate AD (n=40) | NINCDS-ADRDA and MMSE score >15 | 104 | 20IU/d insulin (36) or 40IU/d insulin (38) for 4 months | placebo (30) | placebo 43.3%, 20IU 38.9%, 40IU 47.4% | placebo 74.9, 20IU 72.8, 40IU 69.9 | ADAS-Cog; ADCS-ADL; DSRS |
|  |  |  |  |  |  |  |  |  |  | *In substudy:* |
|  |  |  |  |  |  |  |  |  |  | PET (n = 40; metabolic rate of glucose); CSF (n = 23; Aβ42, Aβ40, tau protein and P181-tau) |
| Crapper McLachlan et al., 1991/ Crapper McLachlan et al., 1993[34,35] | Canada and USA | RCT | AD (not specified if mild or moderate but all living at home) | NINCDS-ADRDA | 48 | desferrioxamine 250 mg intramuscularly daily, 5 days per week, for 24 months (25) | lethicin (9), No treatment (14) | DFO 52%, placebo 52% | 63.1 | *Primary:* |
|  |  |  |  |  |  |  |  |  |  | videorecorder home-behavioural assessment |
|  |  |  |  |  |  |  |  |  |  | *Secondary:* |
|  |  |  |  |  |  |  |  |  |  | Wechsler Adult Intelligence Scale-revised; Wechsler Memory Scale; Western Aphasia Battery |
| Cucinotta et al., 1998[36] | Italy | RCT | mild to moderate AD | NINCDS-ADRDA and MMSE score 15-23 | 142 | dihydroergocryptine 40mg/d for 1 year (70) | placebo (72) | 70% | 74.2 (63-83) | *Primary:* |
|  |  |  |  |  |  |  |  |  |  | GBS scale |
|  |  |  |  |  |  |  |  |  |  | *Secondary:* |
|  |  |  |  |  |  |  |  |  |  | Mental deterioration battery |
| Dodel et al., 2013[37] | Germany and USA | RCT | mild to moderate AD | NINCDS-ADRDA and MMSE score 16-26 | 55 | intravenous immunoglobulin 0·2 g/kg (12), 0·5 g/kg (15), or 0·8 g/kg (14) every 4 weeks till 20 or 22 weeks | placebo (14) | immunoglobulin 37%, placebo 64% | immunoglobulin 69·4, placebo 72·0 | *Primary:* |
|  |  |  |  |  |  |  |  |  |  | Blood (Aβ1–40) |
|  |  |  |  |  |  |  |  |  |  | *Secondary:* |
|  |  |  |  |  |  |  |  |  |  | Blood (Aβ1–42, and anti-Aβ autoantibodies); CSF (Aβ1–40, Aβ1–42, and anti -Aβ autoantibodies, total tau, and p-tau181); ADAS-Cog; CDR (SB); ADCS-ADL; MMSE; MRI (volumetric); PET (glucose metabolism) |
| Doody et al., 2008[38] | Russia | RCT | mild to moderate AD | DSM-4, NINCDS-ADRDA, and MMSE score 10-24 | 183 | dimebon 40mg/d for 26 weeks (89) | placebo (94) | dimebon 72%, placebo 62% | dimebon 68·1, placebo 68·4 | *Primary:* |
|  |  |  |  |  |  |  |  |  |  | ADAS-Cog |
|  |  |  |  |  |  |  |  |  |  | *Secondary:* |
|  |  |  |  |  |  |  |  |  |  | MMSE; NPI; ADCS-ADL; CIBIC+; ADCS-CGIC |
| Doody et al., 2013/ Doody et al., 2015[39,40] | Argentina, Australia, Belgium, Canada, Chile, Denmark, Finland, France, Germany, India, Israel, Italy, Japan, Poland, South Africa, Spain, Sweden, UK, USA | RCT | mild to moderate AD | NINCDS-ADRDA and MMSE score 16-26 | 1537 | semagacestat 100mg/d (507) or semagacestat 140mg/d (529) for 76 weeks | placebo (501) | 53% | 73.2 | *Primary:* |
|  |  |  |  |  |  |  |  |  |  | ADAS-Cog; ADCS-ADL |
|  |  |  |  |  |  |  |  |  |  | *Secondary:* |
|  |  |  |  |  |  |  |  |  |  | CDR (SB); NPI; MMSE; EQ5D; Blood (Aβ) |
|  |  |  |  |  |  |  |  |  |  | *In substudy:* |
|  |  |  |  |  |  |  |  |  |  | CSF (n = 844; Aβ and tau); MRI (n = 208; volumetric); PET (n = 108; Aβ) |
| Doody et al., 2014 / Liu-Seifert et al., 2015[41,42] | France, Japan, USA | RCT | mild to moderate AD | NINCDS-ADRDA and MMSE score 16-26 | 1659 | solanezumab 400mg intravenously every 4 weeks for 18 months (cohort 1 506; cohort 2 521) | placebo (cohort 1 506; cohort 2 519) | cohort 1: placebo 56.7%, solanezumab 59.1%; cohort 2: placebo 65.7%, solanezumab 50.6% | cohort 1: placebo 74.4, solanezumab 75.0; cohort 2: placebo 72.5, solanezumab 71.5 | *Primary:* |
|  |  |  |  |  |  |  |  |  |  | ADAS-Cog; ADCS-ADL |
|  |  |  |  |  |  |  |  |  |  | *Secondary:* |
|  |  |  |  |  |  |  |  |  |  | CDR (SB); NPI; MMSE; EQ5D; Blood (Aβ1-40, and Aβ1-42); MRI (volumetric) |
|  |  |  |  |  |  |  |  |  |  | *In substudy:* |
|  |  |  |  |  |  |  |  |  |  | CSF (n =121; Aβ1-40, Aβ1-42 and tau); PET (n = 266; amyloid) |
| Endres et al., 2014[43] | Germany | RCT | mild to moderate AD | NINCDS-ADRDA and MMSE score 14–27 | 22 | acitretin 30mg/d (11) for 4 weeks | placebo (11) | placebo 55%, acitretin 82% | placebo 73, acitretin 67 | CSF (Aβ42, phospho-tau, or total tau); MMSE; Neuropsychological test battery (CERAD) |
| Farlow et al., 2012[44] | USA | RCT | mild to moderate AD | MMSE score 15-26 | 52 | solanezumab 100 mg every 4 weeks (10), 100 mg weekly (11), 400 mg every 4 weeks (10), or 400 mg weekly (11) for 12 weeks | placebo (10) | 53.8% | - 1. (53-89) | CSF (Aβ1–40 and Aβ1–42); Blood (Aβ1–40 and Aβ1–42); ADAS-Cog |
|  |  |  |  |  |  |  |  |  |  | *In substudy:* |
|  |  |  |  |  |  |  |  |  |  | PET (n = 24; amyloid) |
| Faux et al., 2010/ Lannfelt et al., 2008[45,46] | Australia, Sweden, UK, USA | RCT | mild to moderate AD | NINCDS-ADRDA, MMSE score 20-26 or ADAS-Cog score 10-25 | 78 | PBT2 50mg/d (20) or PBT2 250mg/d (29) for 12 weeks | placebo (29) | placebo 52%, 50mg 45%, 250mg 52% | placebo 71·6 (60–83), 50mg 72·4 (58–83), 250mg 72·1 (58–83) | Blood (Aβ40, Aβ42, Zn²+, and Cu²); CSF (12 Aβ40, Aβ42, total tau, phoshophorylated tau, Zn²+, and Cu²+); Neuropsychological test battery (NTB); ADAS-Cog; MMSE |
| Faxen-Irving et al., 2013/ Freund-Levi et al., 2006[47,48] | Sweden | RCT | mild to moderate AD | DSM-4 and MMSE score 15-30 | 174 | 1.7g/d DHA and 0.6g/d EPA for 6 months (89) | placebo (85) | intervention 57%, placebo 46% | intervention 72.6, placebo 72.9 | *Primary:* |
|  |  |  |  |  |  |  |  |  |  | MMSE; ADAS-Cog |
|  |  |  |  |  |  |  |  |  |  | *Secondary:* |
|  |  |  |  |  |  |  |  |  |  | CDR (SB and global); Blood (transthyretin) |
|  |  |  |  |  |  |  |  |  |  | *In substudy:* |
|  |  |  |  |  |  |  |  |  |  | CSF (n = 35; transthyretin) |
| Ferrari et al., 1998[49] | Italy | RCT | mild to moderate AD | ICD-10 or DSM-4, NINCDS-ADRDA, and MMSE score 10-23 | 213 | posatirelin 10mg/d for 3 months (107) | placebo (106) | 58% | 78.8 | Rey memory test; GBS scale; MMSE; HAM-D; Global DS |
| Fleisher et al., 2008[50] | USA | RCT | mild to moderate AD | NINCDS-ADRDA | 51 | LY450139 100mg/d (22) or 140mg/d (14) for 14 weeks | placebo (15) | placebo 33%, 100mg 64%, 140mg 43% | placebo 68.7, 100mg 70.8, 140mg 68.1 | *Primary:* |
|  |  |  |  |  |  |  |  |  |  | Blood (Aβ); CSF (Aβ) |
|  |  |  |  |  |  |  |  |  |  | *Secondary:* |
|  |  |  |  |  |  |  |  |  |  | ADAS-Cog; ADCS-ADL |
| Fleisher et al., 2011[51] | USA | RCT | mild to moderate AD | MMSE score 12-20 | 89 | divalproex 10-12mg/kg daily for 24 months (43) | placebo (46) | placebo 67%, divalproex 33% | placebo 76, divalproex 73 | *Primary:* |
|  |  |  |  |  |  |  |  |  |  | MRI (volumetric); NPI |
|  |  |  |  |  |  |  |  |  |  | *Secondary:* |
|  |  |  |  |  |  |  |  |  |  | ADAS-Cog; CDR (SB); MMSE; ADCS-ADL; ADCS-CGIC; CMAI; QOL-AD |
| Fox et al., 2005/ Gilman et al., 2005/ Hock et al., 2003/ Koepsell et al., 2007/ Orgogozo et al., 2003/ Vellas et al., 2009[52-57] | France and USA | RCT | mild to moderate AD | MMSE score 15-26 | 131 | Immunisation AN1792 225μg plus QS-21 50μg at day 0 and months 1, 3, 6, 9, 12 (59 responders) | placebo (72) | not specified | Intervention 74.9, placebo 73.7 | *Primary:* |
|  |  |  |  |  |  |  |  |  |  | Blood (Aβ); DAD; CDR (global); MMSE; ADAS-Cog; MRI (volumetric) |
|  |  |  |  |  |  |  |  |  |  | *Secondary:* |
|  |  |  |  |  |  |  |  |  |  | Neuropsychological test battery (NTB) |
| Galasko et al., 2012[58] | USA | RCT | mild to moderate AD | NINCDS-ADRDA and MMSE score ≥16 | 79 | vitamin C 500mg/d and vitamin E 800IU/d and a-lipoic acid 900mg/d (E/C/ALA) (28) OR 1200mg/d coenzyme Q (25) for 16 weeks | placebo (26) | E/C/ALA 46%, coenzyme Q 44%, placebo 48% | E/C/ALA 73.6, coenzyme Q 71.4, placebo 73.2 | ADCS-ADL; MMSE; CSF (tau, phosphorylated tau, Aβ42, F2-isoprostane) |
| Galasko et al., 2014[59] | USA | RCT | mild to moderate AD | MMSE score 14-26 | 399 | PF-04494700 5mg/d (132) or PF-04494700 20mg/d (135) for 18 months | placebo (132) | 5mg/d 53%, 20mg/d 61%, placebo 57% | 5mg/d 73.6, 20mg/d 73.0, placebo 72.2 | *Primary:* |
|  |  |  |  |  |  |  |  |  |  | ADAS-Cog |
|  |  |  |  |  |  |  |  |  |  | *Secondary:* |
|  |  |  |  |  |  |  |  |  |  | CDR (SB); ADCS-ADL; NPI; MMSE; Digit Symbol Substitution Test; Forward and Backward Digit Span Test; Controlled Oral Word Association Test; Stroop Color Word Interference Test; Trail making test; DEMQOL; Blood (Aβ1-40, Aβ1-x, Aβ1-42) |
|  |  |  |  |  |  |  |  |  |  | *In substudy:* |
|  |  |  |  |  |  |  |  |  |  | MRI (n = 186; volumetric); CSF (n = 52; Aβ1-40, Aβ1-x, Aβ1-42, tau and phosphorylated tau) |
| Gauthier et al., 2015[60] | Canada and USA | RCT | moderate AD | NINCDS-ADRDA and MMSE score 10-20 | 403 | ST101 10mg/d (50), ST101 60mg/d (51) and ST101 120mg/d (51) for 12 weeks | placebo (51) | placebo 41.2%, 10mg 50%, 60mg 60.8%, 120mg 52.9% | placebo 78.3, 10mg 74.4, 60mg 77.8, 120mg 75.7 | *Primary:* |
|  |  |  |  |  |  |  |  |  |  | ADAS-Cog |
|  |  |  |  |  |  |  |  |  |  | *Secondary:* |
|  |  |  |  |  |  |  |  |  |  | ADCS-CGIC; ADCS-ADL; NPI; MMSE |
| Geldmacher et al., 2011[61] | USA | RCT | mild to moderate AD | NINCDS-ADRDA and MMSE score 12-26 | 29 | pioglitazone 45mg/d (14) for 18 months | placebo (15) | pioglitazone 64%, placebo 60% | pioglitazone 74.9, placebo 67.0 | *Secondary:* |
|  |  |  |  |  |  |  |  |  |  | CDR (SB); ADAS-Cog; NPI; ADFACS; CIBIC+; NOSGER |
| Gold et al., 2010[62] | Austria, Bulgaria, Chile, China, Croatia, Estonia, Germany, Greece, Hungary, Korea, Mexico, New Zealand, Pakistan, Peru, Philippines, Puerto Rico, Russia, UK and USA | RCT | mild to moderate AD | NINCDS-ADRDA and MMSE score 10-23 | 581 | RSG XR 2mg/d (166), RSG XR 8mg/d (165) for 24 weeks | placebo (166) or donepezil 10mg/d (84) | 2mg/d 64%, 8mg/d 65%, placebo 60%, donepezil 63% | 2mg/d 71.7, 8mg/d 72.6, placebo 72.5, donepezil 72.9 | *Primary:* |
|  |  |  |  |  |  |  |  |  |  | ADAS-Cog; CIBIC+ |
|  |  |  |  |  |  |  |  |  |  | *Secondary:* |
|  |  |  |  |  |  |  |  |  |  | NPI; DAD; MMSE; Blood (glycated haemoglobin); EQ5D |
| Green et al., 2009/ Myriad Pharmaceuticals, 2010[63,64] | USA | RCT | mild to moderate AD | MMSE score 15-26 | 1684 | tarenfurbil 800mg/d (42) or 1600mg/d (820) for 18 months | placebo (822) | 50.9% | 74.6 (55+) | *Primary:* |
|  |  |  |  |  |  |  |  |  |  | ADAS-cog; ADCS-ADL |
|  |  |  |  |  |  |  |  |  |  | *Secondary:* |
|  |  |  |  |  |  |  |  |  |  | CDR (SB); MMSE; NPI; QOL-AD |
| Grimaldi et al., 2014[65] | Italy | RCT | mild to moderate AD | DSM-4 and MMSE score 20-26 | 42 | 66mcg/d interferon beta 1a for 28 weeks (23) | placebo (19) | placebo 58%, IFNB1a 65% | placebo 64.6, IFNB1a 63.0 | ADAS-Cog; Global DS; CIBIC+; MMSE; ADAS-Noncog; Lawton IADL; PSMS; Geriatric DS |
| Hampel et al., 2009[66] | Germany | RCT | mild AD | DSM-4, NINCDS-ADRDA, and MMSE score 21-26 | 71 | lithium, various doses for 10 weeks (33) | Placebo (38) | 52.1% | 68.6 (50-84) | *Primary:* |
|  |  |  |  |  |  |  |  |  |  | CSF (phosphorylated tau) |
|  |  |  |  |  |  |  |  |  |  | *Secondary:* |
|  |  |  |  |  |  |  |  |  |  | CSF (total tau and Aβ42); Blood (Aβ42); MMSE; NPI; ADAS-Cog |
| Hock et al., 2003/ Hock et al., 2000[67,68] | Germany and Switzerland | RCT | mild to moderate AD | NINCDS-ADRDA and MMSE score 12-26 | 40 | talsaclidine, various doses for 4 weeks (34) | placebo (6) | talsaclidine 56%, placebo 83% | talsaclidine 67.1, placebo 69.7 | CSF (Aβ42 and Aβ40) |
| Jhee et al., 2004[69] | Korea and USA | RCT | mild to moderate AD | NINCDS- ADRDA and MMSE score 10-24 | 20 | celecoxib 100mg/d (5), 400mg/d (5) or 800mg/d (5) for 28 days | placebo (5) | placebo 20%, 100mg 20%, 400mg 40%, 800mg 20% | placebo 77.2, 100mg 68.6, 400mg 75.2, 800mg 69.7 | CSF (PGE2, IL-6, Aβ1-42 and tau); Blood (PGE2, IL-6, Aβ1-42 and tau); ADAS-Cog; MMSE; Computerised NTB |
| Jong et al., 2008[70] | The Netherlands | RCT | mild to moderate AD | NINCDS- ADRDA and MMSE score 10-26 | 51 | indomethacin 100mg/d for 12 months (26) | placebo (25) | placebo 76%, indomethacin 54% | placebo 72.2, indomethacin 72.7 | *Primary:* |
|  |  |  |  |  |  |  |  |  |  | ADAS-Cog |
|  |  |  |  |  |  |  |  |  |  | *Secondary:* |
|  |  |  |  |  |  |  |  |  |  | MMSE; CIBIC; ADAS-Noncog; NPI; IDDD |
| Kadir et al., 2008[71] | Sweden | RCT | mild AD | NINCDS- ADRDA and MMSE score ≥21 | 20 | phenserine 30mg/d for 3 months (10) | placebo (10) | 75% | 68 | PET (glucose and amyloid); CSF (Aβ42, total-tau, and phosphorylated tau, alpha and beta-secretase–cleaved amyloid precursor protein); Blood (Aβ40 and Aβ42); MMSE; Recall task; Word recognition; Digit symbol substitution test; Trail making test; Clock drawing task |
| Kessler et al., 2008/ Kessler et al., 2008[72,73] | Germany | RCT | mild AD | NINCDS ADRDA and MMSE score <25 | 68 | verum (51.62mg/d) containing 8mg/d Cu Orotate for 12 months (35) | placebo (33) | placebo 55%, verum 40% | placebo 69.4, verum 69.6 | *Primary:* |
|  |  |  |  |  |  |  |  |  |  | ADAS-Cog; MMSE |
| Landen et al., 2013[74] | Australia, Canada, Sweden, UK | RCT | mild to moderate AD | DSM-4, NINCDS- ADRDA, and MMSE score 16-26 | 37 | ponezumab - 1 infusion of 0.1mg/kg (4), 0.3mg/kg (4), 1mg/kg (4), 3mg/kg (6), or 10mg/kg (8) | placebo (11) | ponezumab (altogether) 42.3%, placebo 27.3% | ponezumab (altogether) 70.0 (50-84), placebo 71.8 (61-85) | ADAS-Cog; MMSE; CogState Alzheimer’s Battery; Blood (Aβ1-x, Aβ1-40, and Aβ1-42) |
|  |  |  |  |  |  |  |  |  |  | *In substudy:* |
|  |  |  |  |  |  |  |  |  |  | CSF (in 1 and 10mg groups, n = 12; Aβ1-x, Aβ1-40, Aβ1-42, tau, and p-tau) |
| Leszek et al., 1999[75] | Poland | RCT | mild to severe AD | NINCDS- ADRDA and DSM-3 | 46 | colostrinin 100mg every second day for 3 weeks followed by a 2-week hiatus - 10 cycles (15) | selenium 100mg (15) or placebo (16) | colostrinin 80%, selenium 80%, placebo 62.5% | colostrinin 70.75 (45–83), selenium 70.75 (50–82), placebo 67.8 (59–76) | *Primary:* |
|  |  |  |  |  |  |  |  |  |  | MMSE |
|  |  |  |  |  |  |  |  |  |  | *Secondary:* |
|  |  |  |  |  |  |  |  |  |  | Additional psycho-social functioning of the AD patients provided by the patients’ caregivers - no information as to what measures |
| Li, 2015[76] | China | CCT | moderate AD | MMSE score 10-20 and ADAS-Cog score 29-40 | 24 | cistanches herba 0.9g/d for 48 weeks (10) | no treatment (6) or Donepezil 5mg/d (8) | cistanches herb 60%, no treatment 50%, donepezil 62.5% | cistanches herb 70.3, no treatment 71.3, donepezil 73.5 | MMSE; ADAS-Cog; MRI (volumetric); CSF (protein, MRNA levels, total-tau, tumor-necrosis factor alpha, and interlukin 1beta) |
| Lovestone et al., 2015[77] | Finland, France, Germany, Spain, UK | RCT | mild to moderate AD | NINCDS-ADRDA and MMSE score 14-26 | 307 | tideglusib 500mg/d (45), 1000mg/d (86), or 1000mg every other day (91) for 26 weeks | placebo (85) | placebo 55.3%, 500mg 64%, 1000mg 51.9%. 1000mg every other day 54.4% | placebo 70.8, 500mg 71.1, 1000mg 72.3, 1000mg every other day 71.6 | *Primary:* |
|  |  |  |  |  |  |  |  |  |  | ADAS-Cog |
|  |  |  |  |  |  |  |  |  |  | *Secondary:* |
|  |  |  |  |  |  |  |  |  |  | MMSE; Fluency test; ADCS-ADL; EQ5D; NPI; CGI |
|  |  |  |  |  |  |  |  |  |  | *In substudy:* |
|  |  |  |  |  |  |  |  |  |  | MRI (n = 86; cerebral atrophy); CSF (n = 21; phosphorylated tau and Aβ1-42) |
| Maher-Edwards et al., 2015[78] | Bulgaria, Canada, Germany, Italy, Spain, Sweden | RCT | mild AD | NINCDS-ADRDA and MMSE score 10-26 | 124 | rilapladib 250mg/d for 24 weeks (62) | placebo (62) | placebo 54%, rilapladib 47% | placebo 73.1, rilapladib 72.9 | *Primary:* |
|  |  |  |  |  |  |  |  |  |  | CogState Alzheimer’s Battery; CSF (Aβ1-42) |
|  |  |  |  |  |  |  |  |  |  | *Secondary:* |
|  |  |  |  |  |  |  |  |  |  | CSF (Aβ1–40, total tau, 181 phosphorylated tau, Lp-PLA2, neurofilament light chain and Albumin quotient); Blood (Aβ1–40, Aβ1–42, and Lp-PLA2) |
| Marcusson et al., 1997[79] | Belgium, Croatia, France, Germany, Sweden, UK, Yugoslavia | RCT | mild to moderate AD or vascular dementia | DSM-3 and MMSE score 15-25 | 261 | 300mg propentofylline 3 times daily for 12 months (130) | placebo (131) | not specified | placebo 72.9, propentofylline 71.9 | *Primary:* |
|  |  |  |  |  |  |  |  |  |  | GBS scale; CGI; SKT |
|  |  |  |  |  |  |  |  |  |  | *Secondary:* |
|  |  |  |  |  |  |  |  |  |  | Digit symbol substitution test; MMSE; NAI-ADL; AMS |
| Molloy et al., 2013[80] | Canada | RCT | mild to moderate AD | NINCDS-ADRDA and MMSE score 14-26 | 406 | doxycycline 200mg/d + rifampin 300mg/d (101), doxycycline 200mg/d (102) or rifampin 300mg/d (101) for 12 months | placebo (102) | doxycycline and rifampin 50.5%, doxycycline 50%, rifampin 48% | doxycycline and rifampin 79.2, doxycycline 78.7, rifampin 78.6 | *Primary:* |
|  |  |  |  |  |  |  |  |  |  | ADAS-Cog; CDR |
|  |  |  |  |  |  |  |  |  |  | *Secondary:* |
|  |  |  |  |  |  |  |  |  |  | MMSE; Geriatric DS; SDD; Lawton IADL; DBRI |
| Muresanu et al., 2002[81] | Austria and Romania | RCT | mild to moderate AD | NINCDS-ADRDA, DSM-4, and MMSE score 14-25 | 60 | cerebrolysin 30ml/d for 5 days/week for 6 weeks (30) | placebo (30) | not specified | not specified | DAD; ADAS-Cog; CGI |
| Muresanu et al., 2008[82] | Romania and Spain | RCT | mild to moderately severe vascular dementia | NINDS-AIREN and MMSE score 9-26 | 41 | cerebrolysin 10ml/d (16) or 30ml/d (15) 5 days/week for 4 weeks. | placebo (10) | 51% | 70.7 (51–88) | ADAS-Cog; MMSE; EEG |
| Nygaard et al., 2015[83] | USA | RCT | mild to moderate AD | MMSE score 16-26 | 24 | AZD0530 50mg/d, 100mg/d, or 125mg/d for 4 weeks. 6 in each of the 3 treatment groups | placebo (6) | 61% | 73 | ADAS-Cog; ADCS-ADL; NPI; CDR (SB); MMSE; PET (glucose); CSF (Aβ40, Aβ42, Tau and P231-Tau) |
| Ostrowitzki et al., 2012[84] | Denmark, Israel, Netherlands, Sweden, UK | RCT | mild to moderate AD | NINCDS- ADRDA and MMSE score 16-26 | 18 | intravenous gantenerumab 60mg (8) or 200mg (6) every 4 weeks for 3 months | placebo (4) | placebo 75%, 60mg 25%, 200mg 50% | placebo 62.8, 60mg 70.9, 200mg 66.5 | *Primary:* |
|  |  |  |  |  |  |  |  |  |  | ADAS-Cog; MMSE; a modified NTB (not specified); DAD |
|  |  |  |  |  |  |  |  |  |  | *Secondary and in substudy:* |
|  |  |  |  |  |  |  |  |  |  | PET (n = 16; amyloid) |
| Quinn et al., 2010[85] | USA | RCT | mild to moderate AD | MMSE score 14-26 | 402 | docosahexaenoic acid (DHA) 2g/d for 18 months (238) | placebo (164) | 52.2% | 76 | *Primary:* |
|  |  |  |  |  |  |  |  |  |  | ADAS-Cog; CDR (SB) |
|  |  |  |  |  |  |  |  |  |  | *Secondary:* |
|  |  |  |  |  |  |  |  |  |  | MMSE; ADCS-ADL; NPI; QOL-AD |
|  |  |  |  |  |  |  |  |  |  | *In substudy:* |
|  |  |  |  |  |  |  |  |  |  | MRI (n = 102; volumetric) |
| Regland et al., 2001[86] | Sweden | RCT | mild to moderate AD | NINCDS- ADRDA and MMSE score 10-24 | 20 | clioquinol 20mg/d (10) or 80mg/d (10) for 21 days |  | 65% | 74.6 (61–83) | CSF (Aß42, Tau and GAP43); MMSE; ADAS-Cog; GBS scale |
| Reines et al., 2004[87] | USA | RCT | mild to moderate AD | NINCDS- ADRDA and MMSE score 14-26 | 692 | rofecoxib 25mg/d for 12 months (346) | placebo (346) | placebo 52%, rofecoxib 54% | placebo 75, rofecoxib 76 | ADAS-Cog; CDR (global); MMSE; ADCS-ADL; CIBIC+ |
| Ringman et al., 2012[88] | USA | RCT | mild to moderate AD | NINCDS- ADRDA and MMSE score 17-29 | 36 | curcumin C3 complex 2g/d (12) or 4d/d (12) for 24 weeks | placebo (12) | 63% | 73.5 | ADAS-Cog; NPI; ADCS-ADL; MMSE; Blood (Aß1-40 and Aß1-42); CSF (Aß1-42, total tau, phosphorylated tau 181 and isoprostanes) |
| Ritchie et al., 2003[89] | Australia | RCT | moderately severe AD | ADAS-Cog score 20-45 and MMSE score 10-24 | 36 | clioquinol ascending doses up to 750mg/d (18) | placebo (18) | clioquinol 50.0%, placebo 43.7% | 72.5 | ADAS-Cog; Blood (Aß, zinc, and copper) |
| Ruether et al., 2000/ Ruether et al., 1994[90,91] | Austria and Germany | RCT | mild to moderate AD | DSM-3 | 120 | cerebrolysin 30ml/d (for 5 days a week) for 4 weeks (60) | placebo (60) | 66% | (55-85) | CGI; NAI-ADL; SCAG; Trail making test; AMS |
| Ruether et al., 2001/ Ruether et al., 2002[92,93] | Austria and Germany | RCT | mild to moderate AD | NINCDS-ADRDA, ICD-10, and MMSE score 14-24 | 149 | cerebrolysin 30ml/d for 5 days a week for 4 weeks (76) | placebo (73) | cerebrolysin 64.9%, placebo 51.4% | cerebrolysin 72.5, placebo 73.5 | *Primary:* |
|  |  |  |  |  |  |  |  |  |  | ADAS-Cog; CGI |
|  |  |  |  |  |  |  |  |  |  | *Secondary:* |
|  |  |  |  |  |  |  |  |  |  | SKT; MADR-S; NAI-ADL; ADAS-Noncog |
| Salloway et al., 2011[94] | Canada and USA | RCT | moderate AD | MMSE score 16-26 | 353 | ELND005 500mg/d (89), 2000mg/d (89), or 4000mg/d (91) twice daily for 78 weeks | placebo (84) | placebo 56.5%, 500mg 58.0%, 2000mg 53.9%, 4000mg 56.0% | placebo 73.4, 500mg 73.4, 2000 mg 73.4, 4000 mg: 72.2 | *Primary:* |
|  |  |  |  |  |  |  |  |  |  | Neuropsychological test battery (NTB); ADCS-ADL |
|  |  |  |  |  |  |  |  |  |  | *Secondary:* |
|  |  |  |  |  |  |  |  |  |  | ADAS-Cog; CDR (SB); NPI; MRI (volumetric) |
|  |  |  |  |  |  |  |  |  |  | *In substudy:* |
|  |  |  |  |  |  |  |  |  |  | MRS (n not specified); CSF (n = 20; Aßx-40, Aßx-42, total tau, phosphorylated-tau) |
| Salloway et al., 2014[95] | Austria, Canada, Germany, US | RCT | mild to moderate AD | MMSE score 16-26 | 2451 | APOE ε4 carriers: 0.5mg/kg of bapineuzumab every 13 weeks up to 78 weeks (673); noncarriers: 0.5mg/kg (337), 1.0mg/kg (329) or 2.0mg/kd (141 but discontinued and received 1.0mg/kg) of bapineuzumab every 13 weeks up to 78 weeks | placebo (carriers: 448; noncarriers 524) | carriers: placebo 56.0%, 0.5mg/kg 54.4% ; noncarriers: placebo 50.3%, 0.5mg/kg 52.5%, 1.0mg/kg 57.0% | carriers: placebo 72.3, 0.5mg/kg 72.0 ; noncarriers: placebo 71.9, 0.5mg/kg 73.1, 1.0mg/kg 73.5 | *Primary:* |
|  |  |  |  |  |  |  |  |  |  | *ADAS-Cog; DAD* |
|  |  |  |  |  |  |  |  |  |  | *Secondary:* |
|  |  |  |  |  |  |  |  |  |  | Neuropsychological test battery (NTB); CDR (SB); MMSE; Dependence Scale |
|  |  |  |  |  |  |  |  |  |  | *In substudy:* |
|  |  |  |  |  |  |  |  |  |  | PET (n = 154; amyloid); CSF (n = 390; phosphorylated-tau 181); MRI (n = 1149; volumetric) |
| Sano et al., 1996[96] | USA | RCT | moderate AD | NINDCS-ADRDA | 486 | selegiline (4mg/day) and a-toc (1,000 IU/day), placebo and a-toc, selegine and placebo for 2 years (n not specified) | placebo (n not specified) | 64.9% | 73.3 | *Primary:* |
|  |  |  |  |  |  |  |  |  |  | Bristol ADL; CDR (global) |
|  |  |  |  |  |  |  |  |  |  | *Secondary:* |
|  |  |  |  |  |  |  |  |  |  | ADAS-Cog; MMSE; Dependence scale; CERAD behavioural rating scale |
| Sano et al., 2011[97] | USA | RCT | mild to moderate AD | MMSE score 12-26 | 406 | simvastatin ascending dose 40m/g for 18 months (204) | placebo (202) | placebo 59.9%, simvastatin 58.8% | placebo 75.1, simvastatin 74.0 | *Primary:* |
|  |  |  |  |  |  |  |  |  |  | ADAS-Cog |
|  |  |  |  |  |  |  |  |  |  | *Secondary:* |
|  |  |  |  |  |  |  |  |  |  | ADCS-CGIC; MMSE; Dependence Scale; ADCS-ADL; NPI; QOL-AD |
| Scharf et al., 1999[98] | Australia | RCT | mild to moderate AD | DSM-4 and MMSE score 11-25 | 41 | diclofenac 100mg/d and misoprostol 400ug/d for 25 weeks (24) | placebo (17) | D/M group 67%, placebo 47% | D/M group 71.8, placebo 73.9 | *Primary:* |
|  |  |  |  |  |  |  |  |  |  | ADAS-Cog; Global DS; CGI |
|  |  |  |  |  |  |  |  |  |  | *Secondary:* |
|  |  |  |  |  |  |  |  |  |  | MMSE; ADAS-Noncog; Lawton IADL; PSMS |
| Schwam et al., 2014[99] | Canada, Chile, Czech Republic, USA | RCT | mild to moderate AD | MMSE score 14-26 | 191 | PF-04447943 50mg/d for 12 weeks (91) | placebo (100) | PF-04447943 64%, placebo 64% | PF-0444794 73.6, placebo 73.5 | ADAS-Cog; NPI; CGI |
| Ser et al., 2013[100] | Germany | RCT | mild to moderate AD | NINCDS-ADRDA and MMSE score 16-26 | 30 | Tideglusib up to 1000mg/d (20) for 20 weeks | placebo (10) | tideglusib 65%, placebo 70% | tideglusib 73.1, placebo 72.6 | *Secondary:* |
|  |  |  |  |  |  |  |  |  |  | MMSE; ADAS-Cog*;* Fluency test; CGI |
| Sevigny et al., 2008[101] | USA | RCT | mild to moderate AD | MMSE score 14-26 | 563 | MK-677 25mg/d for 12 months (282) | placebo (281) | MK-677 56%, placebo 59.8% | MK-677 75.9, placebo 76.1 | *Primary:* |
|  |  |  |  |  |  |  |  |  |  | CIBIC+ |
|  |  |  |  |  |  |  |  |  |  | *Secondary:* |
|  |  |  |  |  |  |  |  |  |  | ADAS-Cog; CDR (SB); ADCS-ADL |
| Siemers et al., 2010[102] | USA | RCT | mild to moderate AD | NINCDS-ADRDA and MMSE score 14-26 | 19 | solanezumab 0.5 mg/kg (4), 1.5 mg/kg (4), 4.0 mg/kg (4), or 10.0 mg/kg (4) single dose | placebo (3) | placebo 100%, 0.5mg/kg 25%, 1.5mg/kg 25%, 4mg/kg 50%, 10mg/kg 25% | placebo 70.3, 0.5mg/kg 61.0, 1.5mg/kg 71.5, 4mg/kg 67.5, 10mg/kg 75.3 | Blood (Aß1-40 and Aß1-42); CSF (Aß1-40 and Aß1-42); ADAS-Cog |
| Silverberg et al., 2002[103] | USA | RCT | mild to moderate AD | MMSE score 15-24 | 29 | surgical shunt for low-flow CSF drainage (15) | no shunt (14) | 48% | 72.4 | *Primary:* |
|  |  |  |  |  |  |  |  |  |  | MDRS; MMSE |
|  |  |  |  |  |  |  |  |  |  | *Secondary:* |
|  |  |  |  |  |  |  |  |  |  | CSF (MAP-tau and Aß1-42) |
| Silverberg et al., 2008[104] | USA | RCT | mild to moderate AD | MMSE score 15-24 | 164 | low-flow ventriculoperitoneal shunt (Cognishunt) (110) | sham (occluded) shunt (120) | occluded 56%, cognishunt 61% | occluded 74.0, cognishunt 74.5 | *Primary:* |
|  |  |  |  |  |  |  |  |  |  | MDRS; Global DS |
|  |  |  |  |  |  |  |  |  |  | *Secondary:* |
|  |  |  |  |  |  |  |  |  |  | CSF (Aß1-42 and MAP-tau) |
| Simons et al., 2002[105] | Germany | RCT | mild to moderate AD | NINCDS-ADRDA and MMSE score 12-26 | 44 | simvastatin 80mg/d for 26 weeks (24) | placebo (20) | placebo 47%, simvastatin 63% | placebo 68.5, simvastatin 68.0 | CSF (AßA40, AßA42, lathosterol, cholesterol, and 24S-hydroxycholesterol); ADAS-Cog;MMSE |
| Soininen et al., 2006[106] | Australia, Belgium, Finland, France, Germany, Netherlands, and UK | RCT | mild to moderate AD | NINCDS-ADRDA and MMSE score 12-26 | 425 | celecoxib 400mg/d for 52 weeks (285) | placebo (140) | placebo 59%, celecoxib 53% | placebo 73.3, celecoxib 73.7 | *Primary:* |
|  |  |  |  |  |  |  |  |  |  | ADAS-Cog; CIBIC+ |
|  |  |  |  |  |  |  |  |  |  | *Secondary:* |
|  |  |  |  |  |  |  |  |  |  | Behave-AD; NOSGER; MMSE |
| Sparks et al., 2005[107] | USA | RCT | mild to moderate AD | NINCDS-ADRDA and MMSE score 12-28 | 63 | atorvastatin calcium 80mg/d for 12 months (32) | placebo (31) | placebo 35.5%, atorvastatin calcium 37.5% | placebo 78.9, atorvastatin calcium 78.15 | *Primary:* |
|  |  |  |  |  |  |  |  |  |  | ADAS-Cog; CGI |
|  |  |  |  |  |  |  |  |  |  | *Secondary:* |
|  |  |  |  |  |  |  |  |  |  | MMSE; Geriatric DS; ADCS-ADL |
| Sweetlove, 2012[108] | New Zealand | RCT | mild to moderate AD | MMSE score 12-24 | 1003 | dimebon 15 mg/d or 60 mg/d (n not specified) | placebo (n not specified) | males and females (n not specified) | (50+) | ADAS-Cog; ADCS-ADL |
| Tan, 2003[109] | USA | RCT | mild to moderate AD | NINCDS-ADRDA | 10 | intramuscular testosterone enanthate, 200mg every 2 weeks for 12 months (5) | placebo (5) | 0% (all male) | 72.4 (68-80) | *Primary:* |
|  |  |  |  |  |  |  |  |  |  | ADAS-Cog; MMSE |
|  |  |  |  |  |  |  |  |  |  | *Secondary:* |
|  |  |  |  |  |  |  |  |  |  | Clock drawing test |
| Turner et al., 2015[110] | USA | RCT | mild to moderate AD | NINCDS-ADRDA and MMSE score 14-26 | 119 | resveratrol escalating doses up to 2000 mg/d (64) | placebo (55) | resveratrol 62.5%, placebo 51% | resveratrol 69.8, placebo 73 | *Primary:* |
|  |  |  |  |  |  |  |  |  |  | Blood (Aß40 and Aß42, and insulin and glucose); CSF (Aß40, Aß42, tau, and phosphorylated-tau 181); MRI (volumetric) |
|  |  |  |  |  |  |  |  |  |  | *Secondary:* |
|  |  |  |  |  |  |  |  |  |  | MMSE; ADAS-Cog; ADCS-ADL; CDR (SB); NPI |
| Van Gool et al., 2001[111] | Netherlands | RCT | mild AD | Minimal or mild severity scores according to the CAMDEX | 168 | single dose of hydroxychloroquine (83; 400 mg in patients weighing ≥65 kg or 200 mg in those weighing <65 kg) | placebo (85) | Hydroxychloroquine 54%, placebo 60% | hydroxychlorquine 70.4, placebo 70.7 | *Primary:* |
|  |  |  |  |  |  |  |  |  |  | IDDD |
|  |  |  |  |  |  |  |  |  |  | *Secondary:* |
|  |  |  |  |  |  |  |  |  |  | ADAS-Cog; RMBCP |
| Vellas et al., 2011[112] | France | RCT | mild to moderate AD | NINCDS-ADRDA and MMSE score 12-24 | 159 | EHT0202 40mg/d (51) or 80 mg/d (55) for 3 months | placebo (53) | 56% | 40mg/d 76.4, 80mg/d 76.7, placebo 75.8 | *Primary:* |
|  |  |  |  |  |  |  |  |  |  | ADAS-Cog; Neuropsychological test battery (NTB); CDR (SB); NPI; ADCS-ADL; MMSE; CGI |
|  |  |  |  |  |  |  |  |  |  | *Secondary:* |
|  |  |  |  |  |  |  |  |  |  | Blood (sAPPα) |
| Wang et al., 2013[113] | China | RCT | moderate to severe AD | NINCDS-ADRDA, DSM-4, and MMSE score 4-20 | 26 | memantine 10mg/d for 24 weeks (13) | placebo (13) | placebo 54%, memantine 54% | placebo 64.7, memantine 65.7 (50-90) | *Primary:* |
|  |  |  |  |  |  |  |  |  |  | Severe Impairment Battery; PET (glucose); CSF (t-tau, p-tau181, Aβ40, and Aβ42) |
|  |  |  |  |  |  |  |  |  |  | *Secondary:* |
|  |  |  |  |  |  |  |  |  |  | ADAS-Cog; MMSE; NPI |
| Watson et al., 2005[114] | USA | RCT | amnestic MCI (n=9) or mild AD (n=21) | NINCDS-ADRDA and MMSE score >15 | 36 | rosiglitazone 4mg/d for 6 months (24) | placebo (12) | 30% | rosiglitazone 72.8, placebo 73.3 | Blood (Insulin and Aβ); Buschke Selective Reminding Test; Recall task; Stroop Color–Word Interference; Trail making test; Fluency test |
| Wilcock et al., 2008[115] | Canada and UK | RCT | mild to moderate AD | MMSE score 15-26 | 210 | tarenflurbil 400mg/d (69) or 800mg/d (70) for 12 months | placebo (71) | placebo 48%, 400mg/d 52%, 800mg/d 48% | placebo 74·4, 400mg/d 73·4, 800mg/d 75·8 | ADAS-Cog; ADCS-ADL; CDR (SB) |
| Winblad et al., 2001[116] | Belgium, Germany, Italy, Sweden, UK | RCT | mild to moderate AD | MMSE score 12-24 | 346 | nicergoline 60mg/d for 6 months (177) | placebo (169) | nicergoline 61%, placebo 63.9% | 73.7 | *Primary:* |
|  |  |  |  |  |  |  |  |  |  | ADAS-Cog; CGI; ADAS-Noncog |
|  |  |  |  |  |  |  |  |  |  | *Secondary:* |
|  |  |  |  |  |  |  |  |  |  | Lawton IADL; PSMS |
| Winblad et al., 2012[117] | Sweden | RCT | mild to moderate AD | DSM-4 and MMSE score 16-26 | 58 | cohort 1: 50mg CAD106 injection at 0, 6, 18 weeks (24); cohort 2: 150mg CAD106 injection at 0, 2, 6 weeks (22) | placebo (cohort 1: 7, cohort 2: 5) | cohort 1: CAD106 33%, placebo 57%; cohort 2: CAD106 59%, placebo 60% | cohort 1: CAD106 68·9, placebo 70·6; cohort 2: CAD106 68·2, placebo 67·0 | *Primary:* |
|  |  |  |  |  |  |  |  |  |  | CSF (serum Aβ-antibody) |
|  |  |  |  |  |  |  |  |  |  | *Secondary:* |
|  |  |  |  |  |  |  |  |  |  | CSF (Amyloid Biomarkers); Blood (Amyloid Biomarkers); Neuropsychological test battery (CERAD); MMSE; CDR (global); ADCS-ADL; MRI (volumetric) |
| Wischik, 2014[118] | Singapore and UK | RCT | mild to moderate AD | DSM-4, NINCDS-ADRDA, and MMSE score 10-26 | 321 | methylthioninium 69 mg/d (59), 138 mg/d (80) or 228 mg/d (90) for 24 weeks | placebo (92) | 54% | 73.8 | *Primary:* |
|  |  |  |  |  |  |  |  |  |  | ADAS-Cog |
|  |  |  |  |  |  |  |  |  |  | *Secondary:* |
|  |  |  |  |  |  |  |  |  |  | ADCS-CGIC; MMSE; CDR (SB); Bristol ADL; ADFACS; NPI; dementia ‘caseness' short CAMDEX |
|  |  |  |  |  |  |  |  |  |  | *In substudy:* |
|  |  |  |  |  |  |  |  |  |  | SPECT (n = 135; for regional cerebral blood flow) |
| Wolkowitz et al., 2003[119] | USA | RCT | mild to severe AD | MMSE score >8 | 58 | dehydroepiandrosterone (DHEA) 100mg/d for 6 months (28) | placebo (30) | 49% | DHEA 75.5, placebo 77.2 | ADAS-Cog; CIBIC+; MMSE; ADAS-Noncog; CSDD |
| AD, Alzheimer’s disease; ADAS-Cog, Alzheimer's Disease Assessment Scale-Cognitive subscale; ADAS-Noncog, Alzheimer's Disease Assessment Scale - Non-Cognitive subscale; ADCS-ADL, Alzheimer's Disease Co-operative Study - Activities of Daily Living Inventory; ADCS-CGIC, Alzheimer's Disease Cooperative Study - Clinical Global Impression of Change; ADFACS, Alzheimer's Disease Functional Assessment and Change Scale; ADL, activities of daily living; AMS, Adjective Mood Scale; BDRS, Blessed dementia rating scale; Behave-AD, Behavioural Pathology in Alzheimer's Disease Rating Scale; BPRS, Brief Psychiatric Rating Scale; BVRT, Benton Visual Retention Test; CAMDEX, Cambridge Mental Disorders of the Elderly Examination; CDR, Clinical Dementia Rating Scale; CERAD Consortium to Establish a Registry for Alzheimer’s Disease; CGI, Clinical Global Impression; CIBIC+, Clinician's Interview-Based Impression of Change plus carers input; CMAI, Cohen-Mansfield Agitation Inventory; CSF, cerebral spinal fluid; CSDD, Cornell Scale for Depression in Dementia; DAD, Disability Assessment for Dementia; DBRI, The Dysfunctional Behaviour Rating Instrument; DSM, Diagnostic and Statistical Manual of Mental Disorders; DSRS, Dementia Severity Rating Scale; EEG, electroencephalogram; EQ5D, EuroQol five dimensions questionnaire; FAQ, functional activities questionnaire; GBS scale, Gottfries-Brane-Steen Scale; Geriatric DS, Geriatric Depression Scale; Global DS, Global Deterioration Scale; HAM-D, Hamilton Rating Scale for Depression; IADL, instrumental activities of daily living; IDDD, Interview for Deterioration of Daily Living in Dementia; MADR-S, Montgomery-Asberg Depression Rating Scale; MCI, mild cognitive impairment; MDRS, Mattis Dementia Rating Scale; MMSE, Mini Mental State Examination; MRI, Magnetic resonance imaging; MRS, Magnetic resonance spectroscopy; NAI-ADL, Nuremberg Gerontopsychological Rating Scale for Activities of Daily Living; NINCDS-ADRDA, National Institute of Neurological and Communicative Disorders and Stroke/Alzheimer's disease and Related Disorders Association; NOSGER, Nurses' Observation Scale for Geriatric Patients; NPI, Neuropsychiatric Inventory; PET, positron emission tomography; PGRS, Plutchik geriatric rating scale; PSMS, Personal Self Maintenance Scale; QOL-AD, Quality of Life in Alzheimer's Disease; RCT, randomised controlled trial; RMBCP, Revised Memory and Behavioral Problems Checklist; SB, sum of boxes; SCAG, Sandoz Clinical Assessment-Geriatric Scale; SPECT, Single-photon emission computed tomography; SKT, Syndrom Kurt Syndrome Short test. | | | | | | | | | | |

**TABLE B** Published protocols

| **Author and year** | **Trial location** | **Trial type** | **Dementia type and severity** | **Criteria for dementia diagnosis** | **Participants sex** | **Participants age range** | **Description of intervention** | **Description of control/ comparator group** | **Outcomes related to disease modification** |
| --- | --- | --- | --- | --- | --- | --- | --- | --- | --- |
| Annweiler et al., 2011[120] | France | RCT | moderate AD | NINCDS-ADRDA, DSM-4, and MMSE score 10-20 | males and females | ≥60 | memantine 20mg/d plus vitamin D 3,571 IU/d for 24 weeks | memantine 20mg/d plus placebo | *Primary:* |
|  |  |  |  |  |  |  |  |  | ADAS-Cog |
|  |  |  |  |  |  |  |  |  | *Secondary:* |
|  |  |  |  |  |  |  |  |  | MMSE; Frontal Assessment Battery; Trail making test; Katz ADL; Lawton IADL |
| Egefjord et al., 2012[121] | Denmark | RCT | mild to moderate AD | MMSE score 18-21 | not specified | 50-80 | liraglutide 1.8mg/d for 6 months | placebo | *Primary:* |
|  |  |  |  |  |  |  |  |  | PET (glucose uptake and Aβ deposits) |
|  |  |  |  |  |  |  |  |  | *Secondary:* |
|  |  |  |  |  |  |  |  |  | MRI (perfusion); Wechsler Memory Scale (Brief Cognitive Examination) |
| Lawlor et al., 2014[122] | France, Germany, Greece, Holland, Hungary, Ireland, Italy, Sweden and UK | RCT | mild to moderate AD | NINCDS- ADRDA and MMSE score 12-27 | males and females | ≥50 | nilvadipine 8mg/d for 78 weeks | placebo | ADAS-Cog; CDR (SB); DAD |
| AD, Alzheimer’s disease; ADAS-Cog, Alzheimer's Disease Assessment Scale-Cognitive subscale; ADL, activities of daily living; CDR, Clinical Dementia Rating Scale; DAD, Disability Assessment for Dementia; DSM, Diagnostic and Statistical Manual of Mental Disorders; IADL, instrumental activities of daily living; MMSE, Mini Mental State Examination; MRI, Magnetic resonance imaging; NINCDS-ADRDA, National Institute of Neurological and Communicative Disorders and Stroke/Alzheimer's disease and Related Disorders Association; PET, positron emission tomography; RCT, randomised controlled trial. | | | | | | | | | |

**TABLE C** Ongoing trials

| **Trial register number** | **Trial location** | **Trial type** | **Dementia type and severity** | **Criteria for dementia diagnosis** | **Participants sex** | **Participants age range** | **Description of intervention** | **Description of control/ comparator group** | **Outcomes related to disease modification** |
| --- | --- | --- | --- | --- | --- | --- | --- | --- | --- |
| ISRCTN16105064[123] | UK | RCT | early AD | NIAAA criteria and MMSE score >23 | males and females | 45-100 | minocycline 400mg/d or minocycline 200mg/d for 2 years | placebo | MMSE; Bristol ADL |
| ISRCTN31208535[124] | UK | RCT | mild to moderate subcortical ischemic vascular dementia | DSM-4 and MMSE score 15-26 | males and females | ≥ 50 | amlodipine 5 mg/d for 2 weeks then 10mg/d for 50 weeks | placebo | *Primary:* |
|  |  |  |  |  |  |  |  |  | VADAS-Cog |
|  |  |  |  |  |  |  |  |  | *Secondary:* |
|  |  |  |  |  |  |  |  |  | MMSE; Trail making test; TICS-M; CGI; MRI (quantitation of lacunar lesions and diffuse white matter lesions); EQ5D; DEMQOL; DAD; NPI |
| ISRCTN89711766[125] | UK | RCT | early AD | Dubois criteria for early AD or NINCDS-ADRDA/ NIA-AA, and MMSE score ≥22 | males and females | 50-85 | intravenous liraglutide 1.8 mg/d for 12 months | placebo | PET (change in glucose metabolism) |
| ISRCTN93682878[126] | UK | RCT | mild to moderate AD | MMSE score 15-28 or MoCA score 12-24 | males and females | ≥55 | losartan escalating doses to 100mg/d for 12 months | placebo | *Primary:* |
|  |  |  |  |  |  |  |  |  | MRI (whole brain atrophy) |
|  |  |  |  |  |  |  |  |  | *Secondary:* |
|  |  |  |  |  |  |  |  |  | MRI (white matter hyperintensity volume and cerebral blood flow); ADAS-Cog; DEMQOL; NPI; Bristol ADL |
| NCT01409915[127] | USA | RCT | mild to moderate AD | MMSE score 10-26 | males and females | 55-85 | sagramostim (leukine) 250 mcg/m2 subcutaneously for 5 days a week for 3 weeks | placebo | *Secondary:* |
|  |  |  |  |  |  |  |  |  | MMSE; ADAS-Cog; CDR (global); Trail making test; Mohs Number Cancellation Test |
| NCT01561053[128] | Spain | RCT | mild-moderate AD | MMSE score 18-26 | males and females | 55-85 | low dose albumin and immune globulin, high dose albumin and immune globulin, or low dose albumin with no immune globulin | no intervention | *Primary:* |
|  |  |  |  |  |  |  |  |  | ADAS-Cog; ADCS-ADL |
|  |  |  |  |  |  |  |  |  | *Secondary:* |
|  |  |  |  |  |  |  |  |  | MMSE; Neuropsychological test battery (not specified); NPI; CDR (SB); ADCS-CGIC; CSDD; C-SSRS; QoL-AD; PET (glucose metabolism); CSF (Aβ1-40 and Aβ1-42, t-tau and p-tau); Blood (Aβ1-40 and Aβ1-42) |
| NCT01767311[129] | USA and Japan | RCT | MCI or mild AD | NIA-AA and MMSE score ≥22 | males and females | 50-90 | BAN2401 2.5mg/kg, BAN2401 5mg/kg or BAN2401 10mg/kg every 2 weeks; or 5mg/kg or 10mg/kg every 4 weeks, with placebo every 2 weeks |  | *Primary:* |
|  |  |  |  |  |  |  |  |  | AD Composite Score (ADAS-Cog, MMSE, CDR SB) |
|  |  |  |  |  |  |  |  |  | *Secondary:* |
|  |  |  |  |  |  |  |  |  | MRI (volumetric); PET (amyloid) |
| NCT01965756[130] | USA | RCT | early AD | MMSE score >21 | males and females | 55-80 | metformin escalating doses to 4000mg/d - then crossover with placebo | placebo | *Primary:* |
|  |  |  |  |  |  |  |  |  | ADAS-Cog |
|  |  |  |  |  |  |  |  |  | *Secondary:* |
|  |  |  |  |  |  |  |  |  | CogState Alzheimer’s Battery; DSRS; MRI (pCASL, MPRAGE and Flair); CSF |
| NCT01966666[131] | USA | RCT | mild-moderate AD | NIA-AA and MMSE score 14-26 | males and females | 50-82 | TPI-287 2mg/m2, 6.3 mg/m2, or 20 mg/m2 intravenous infusion once every 3 weeks for 9 weeks | placebo | *Secondary:* |
|  |  |  |  |  |  |  |  |  | CSF (biomarkers for AD but not specified); MRI (changes in brain network functional and structural connectivity and perfusion); ADAS-Cog; MMSE; ADCS-ADL; Geriatric DS |
| NCT02036645[132] | USA | RCT | mild-moderate AD | Not specified | males and females | 55-85 | MEDI1814 IV either IV or subcutaneous injection (single or multiple doses, 25-1800mg) | placebo | *Primary:* |
|  |  |  |  |  |  |  |  |  | C-SSRS |
|  |  |  |  |  |  |  |  |  | *Secondary:* |
|  |  |  |  |  |  |  |  |  | Blood (Aβ1-42); CSF (Aβ1-42 and Aβ1-40) |
| NCT02051608[133] | Argentina, Australia, Belgium, Bulgaria, Canada, Denmark, Finland, France, Germany, Guatamala, Hungary, Italy, Japan, Korea, Netherlands, Portugal, Russia, Spain, Sweden, Switzerland, Turkey, UK, USA | RCT | mild AD | NINCDS-ADRDA | males and females | 50-90 | gantenerumab (dose not specified) subcutaneously every 4 weeks for 100 weeks | placebo | *Primary:* |
|  |  |  |  |  |  |  |  |  | ADAS-Cog; ADCS-ADL |
|  |  |  |  |  |  |  |  |  | In subsample: |
|  |  |  |  |  |  |  |  |  | PET (for amyloid) |
|  |  |  |  |  |  |  |  |  | *Secondary:* |
|  |  |  |  |  |  |  |  |  | CSF (t-tau, p-tau and Aβ1-42); NPI; CDR (SB and global); MMSE; MRI (volumetric) |
| NCT02080364[134] | USA | RCT | mild AD | MMSE score 21-26 | males and females | 50+ | azeliragon 5mg/d for 18 months | placebo | *Primary:* |
|  |  |  |  |  |  |  |  |  | ADAS-Cog; CDR (SB) |
|  |  |  |  |  |  |  |  |  | *Secondary:* |
|  |  |  |  |  |  |  |  |  | MRI (volumetric); PET (glucose); NPI; MMSE; ADCS-ADL; Controlled Oral Word Association Test; Fluency test; DEMQOL; Blood (Aβ) |
| NCT02245737[135] | Argentina, Australia, Belgium, Canada, France, Germany, Hungary, Italy, Japan, Poland, Romania, Spain, South Korea, Sweden, UK, USA | RCT | MCI or mild AD | NIA-AA and MMSE score 20-30 | males and females | 55-85 | LY3314814 20mg/d or 50mg/d for 104 weeks | placebo | *Primary:* |
|  |  |  |  |  |  |  |  |  | CDR (global) |
|  |  |  |  |  |  |  |  |  | *Secondary:* |
|  |  |  |  |  |  |  |  |  | ADAS-Cog; FAQ; ACDS-ADL; NPI |
|  |  |  |  |  |  |  |  |  | *In substudy:* |
|  |  |  |  |  |  |  |  |  | CSF (Aβ1-42, Aβ1-40, total tau and partial tau); PET (amyloid and glucose); MRI (volumetric) |
| NCT02322021[136] | USA | RCT | MCI or mild-moderate AD | NIA-AA | males and females | 50-85 | E2609 low, middle or high doses (not specified) for 18 months | placebo | *Secondary:* |
|  |  |  |  |  |  |  |  |  | AD Composite Score (ADAS-Cog, MMSE, CDR SB) |
| NCT02353598[137] | USA | RCT | mild to moderate AD | NINCDS-ADRDA or DSM-5, and MMSE score 18-28 | males and females | 50-90 | intravenous crenezumab dose level 1, 2 or 3 (not specified) every 4 weeks till week 13 | placebo | *Primary:* |
|  |  |  |  |  |  |  |  |  | C-SSRS; MRI (amyloid) |
| NCT02386306[138] | USA | RCT | mild to moderate AD | NIA-AA and MMSE score 12-26 | males and females | 55-85 | GC021109 - one of three different doses (not specified) for 28 days | placebo | *Secondary:* |
|  |  |  |  |  |  |  |  |  | Blood (IL-12, Aβ, and tau); CSF (IL-12, Aβ, and tau) |
| NCT02389413[139] | Belgium, France, Germany, Finland, Netherlands, Sweden | RCT | MCI or mild AD | MMSE score 21-30 | males and females | 50-89 | PQ912 twice daily for 12 weeks | placebo | *Secondary:* |
|  |  |  |  |  |  |  |  |  | MMSE; Fluency tests; Geriatric DS; CogState Alzheimer’s Battery; CSF (QC activity, total-tau, phospho-tau, Aβ pattern, pro-inflammatory panel); MRI (brain functional connectivity); EEG |
| NCT02406027[140] | Belgium, France, Germany, Netherlands, Spain, Sweden | RCT | early AD | Not specified | males and females | 50-85 | JNJ-54861911 10mg/d or 25 mg/d for 52 weeks | placebo | *Secondary:* |
|  |  |  |  |  |  |  |  |  | CSF ((Aβ1-37, Aβ1-38, Aβ1-40, Aβ1-42) and sAPP Fragments (sAPP-alpha, sAPP-beta) and total); Blood (Aβ1-40 Levels and sAPP Fragments (sAPP-alpha, sAPP-beta)) |
| NCT02431468[141] | USA | RCT | moderate severe to severe AD | MMSE score 4-15 | males and females | 55-85 | bryostatin 1 10ug, 20ug, or 40ug via IV for 45 minutes every other week | placebo | Severe impairment battery |
| NCT02434718[142] | Japan | RCT | mild to moderate AD | Not specified | males and females | 55-85 | aducanumab (BIIB037) IV infusion in cohorts assigned to doses (single or multiple) up to 10 mg/kg | placebo | MRI (for amyloid) |
| NCT02477800[143] | Australia, Austria, Canada, Denmark, France, Germany, Hungary, Italy, Japan, Korea, Portugal, Spain, Taiwan, UK, USA | RCT | early AD | MMSE score 24-30 | males and females | 55-85 | aducanumab (BIIB037) low or high dose via monthly intravenous (IV) infusion of for 18 months | placebo | *Primary:* |
|  |  |  |  |  |  |  |  |  | CDR (SB) |
|  |  |  |  |  |  |  |  |  | *Secondary:* |
|  |  |  |  |  |  |  |  |  | MMSE; ADAS-Cog |
| NCT02484547[144] | Belgium, Canada, France, Germany, Italy, Japan, Netherlands, Poland, Spain, Sweden, Switzerland, USA | RCT | early AD | MMSE score 24-30 | males and females | 50-85 | aducanumab (BIIB037) low or high dose via monthly intravenous (IV) infusion of for 18 months | placebo | *Primary:* |
|  |  |  |  |  |  |  |  |  | CDR (SB) |
|  |  |  |  |  |  |  |  |  | *Secondary:* |
|  |  |  |  |  |  |  |  |  | MMSE; ADAS-Cog |
| NCT02503501[145] | USA | RCT | Amnestic MCI or mild AD | NINCDS-ARDRA and MoCA score 18-27 | males and females | 50-90 | insulin glulisine 40IU/d for 6 months | placebo | *Primary:* |
|  |  |  |  |  |  |  |  |  | ADAS-Cog; FAQ; CDR (SB and global); CSF (Aβ42, tau, and phosphorylated-tau); PET (glucose) |
|  |  |  |  |  |  |  |  |  | *Secondary:* |
|  |  |  |  |  |  |  |  |  | Digit Span; Trail making test; Fluency test; Wechsler Memory Scale; Geriatric DS |
| NCT02547818[146] | USA | RCT | early AD | score below the education adjusted cut off on delayed Paragraph Recall (from the Wechsler Memory Scale) | males and females | 55-79 | ALZT-OP1a (cromolyn) and ALZT-OP1b (ibuprofen) together, or cromolyn and placebo, or ibuprofen and placebo | placebo | *Primary:* |
|  |  |  |  |  |  |  |  |  | CDR (SB) |
| NCT02551809[147] | Taiwan | RCT | Mild AD | MMSE score 20-26 | males and females | 60+ | UB-311 either 7 doses or 5 doses (with 2 placebo doses) | placebo | *Secondary:* |
|  |  |  |  |  |  |  |  |  | ADAS-Cog; ADCS-ADL; MMSE; CDR (SB); NPI |
| NCT02579252[148] | Austria | RCT | Mild AD | NIA-AA and MMSE score 20-26 | males and females | 50-85 | AADvac1 (40µg Axon peptide 108) for 8 doses - 6 every 4 weeks, then 2 booster every 6 months | placebo | CogState Alzheimer’s Battery; CDR (SB); PET (for glucose metabolism); MRI (volumetric); CSF (biomarkers not specified) |
| NCT02600130[149] | USA | RCT | mild to moderate AD | DSM-4, NINCDS-ADRDA, and MMSE score 18-24 | males and females | 55-75 | target dose of 20 million or 100 million Longeveron Mesenchymal Stem Cells (LMSCs) via intravenous infusion. | placebo | ADAS-Cog; CogState Alzheimer’s Battery; MMSE; NPI; Geriatric DS ; ADCS-ADL; QOL-AD; CSF (inflammatory biomarkers, and Tau, Phosphorylated tau, and Aβ); MRI (volumetric); Blood (IL-1, IL-6, TGF-β1, TNF-α, CRP, D-Dimer, Fibrinogen, ApoE, and CBC) |
| AD, Alzheimer’s disease; ADAS-Cog, Alzheimer's Disease Assessment Scale-Cognitive subscale; ADCS-ADL, Alzheimer's Disease Co-operative Study - Activities of Daily Living Inventory; ADCS-CGIC, Alzheimer's Disease Cooperative Study - Clinical Global Impression of Change; ADL, activities of daily living; CDR, Clinical Dementia Rating Scale; CGI, Clinical Global Impression; CSF, cerebral spinal fluid; CSDD, Cornell Scale for Depression in Dementia; C-SSRS, Columbia Suicide Severity Rating Scale; DAD, Disability Assessment for Dementia; DSM, Diagnostic and Statistical Manual of Mental Disorders; DSRS, Dementia Severity Rating Scale; EQ5D, EuroQol five dimensions questionnaire; FAQ, functional activities questionnaire; Geriatric DS, Geriatric Depression Scale; Global DS, Global Deterioration Scale; IADL, instrumental activities of daily living; MCI, mild cognitive impairment; MMSE, Mini Mental State Examination; MRI, Magnetic resonance imaging; NIA-AA, National Institute on Aging/Alzheimer’s Association; NINCDS-ADRDA, National Institute of Neurological and Communicative Disorders and Stroke/Alzheimer's disease and Related Disorders Association; NPI, Neuropsychiatric Inventory; PET, positron emission tomography; QOL-AD, Quality of Life in Alzheimer's Disease; RCT, randomised controlled trial; TICS-M, Modified Telephone Interview for Cognitive Status; VADAS-Cog, Vascular Dementia Assessment Scale cognitive subscale. | | | | | | | | | |

**TABLE D** Validation of cognitive outcomes

| **Measure** | **n of trials** | **n of participants** | **Time taken** | **Who completes** | **Relevant populations validated with** | **MCID** | **Ceiling & floor effects** | **Sensitivity to change** | **Acceptability** | **Inter-rater reliability** | **Test-retest reliability** |
| --- | --- | --- | --- | --- | --- | --- | --- | --- | --- | --- | --- |
| *Global:* |  |  |  |  |  |  |  |  |  |  |  |
| Alzheimer's Disease Assessment Scale - Cognitive Scale[150] | 92 (75 published, 2 protocols, and 15 ongoing) | 20,419 | 20-25 minutes | Administered to PWD | Validated in Chinese, Indian, Turkish, Brazilian, and Spanish[151,152]; Validated for mild-moderate dementia[153] | Minimal clinically important difference of 3 may be relevant for early Alzheimer’s[154] and was 4 for VISTA trial[153] | No ceiling or floor effects reported across 20 studies[151]; Ceiling effects in mild dementia[155] | Sensitivity to change across 21 dementia treatment studies[151]; But can have poor sensitivity to detect change in mild-moderate Alzheimer’s[156,157] | No information found | Good across 4 studies[150,158-160] | Good across 7 studies[150,152,155,159-162] but low on some items[155] |
| Mini Mental State Examination[163] | 83 (68 published, 1 protocol, and 14 ongoing) | 17,736 | 5-10 minutes | Administered to PWD | Translated into 50 languages and validated in many including Slovenian, Persian, Urdu, Greek, and Spanish[164-169] | Minimum clinically important difference of 1.4 points in the DOMINO trial[170] | Moderate ceiling effects, and small floor effects[171] | Sensitive to change in Alzheimer’s[172]; May not be sensitive to change in early dementia and dementia with Lewy bodies/ frontotemporal dementia[173] | Described as acceptable to patients[163] | Good[163,174] | Good[175-177] |
| *Batteries:* |  |  |  |  |  |  |  |  |  |  |  |
| CERAD Neuropsychological test battery[178] | 2 (2 published) | 80 | 30 minutes | Interviewer administered with PWD | Validated in French, Korean and Russian and Cantonese[179-182]; Validated for various types of dementia including Alzheimer’s and frontotemporal[183,184] | No information found | No ceiling or floor effects[185] | Sensitive to progression of Alzheimer's[178,186] | No information found | Good inter-rater reliability in PWD living in community[187] | Good[184] |
| CogState Alzheimer’s Battery [188] | 6 (2 published and 4 ongoing) | 161 | 15-20 minutes | Computer administered with PWD | Validated for use in dementia[189] | No information found | No floor or ceiling effects[190,191] | Sensitive to cognitive changes in dementia across 3 studies[190-192] | No information found | No information found | Good in Alzheimer’s[190,193] |
| Neuropsychological Test Battery[194] | 7 (7 published) | 3429 | 70 minutes for all components | Interviewer administered with PWD | Validated for mild to moderate dementia[195]; Validated for use in China, Taiwan, Singapore, Hong Kong, and South Korea[175] | No information found | No floor or ceiling effect in total score, but floor effect on RAVLT delayed recall test in moderate dementia[155] | Good ability to detect change in mild-moderate[155,175,195] | No information found | Good[175] | Good in mild-moderate[155,194,195] |

**TABLE E** Validation of neuropsychiatric outcomes

| **Measure** | **n of trials** | **n of participants** | **Time taken** | **Who completes** | **Relevant populations validated with** | **MCID** | **Ceiling & floor effects** | **Sensitivity to change** | **Acceptability** | **Inter-rater reliability** | **Test-retest reliability** |
| --- | --- | --- | --- | --- | --- | --- | --- | --- | --- | --- | --- |
| Alzheimer's Disease Assessment Scale - Non Cognitive Scale[150] | 7 (all published) | 792 | 20-25 minutes | Interview with PWD and caregiver | Validated for mild to moderate dementia[196] | No information found | No floor or ceiling effects[197] | Sensitivity to change in mild-moderate[161,198] | No information found | Good across 3 studies[158,161,199] | Good[161] |
| Behavioural Pathology in Alzheimer's Disease Rating Scale[200] | 1 (published) | 425 | 20 minutes | Informant interview by clinician | Validated in French, Swedish, German, Dutch, Spanish, Chinese, and Korean[151] | No information found | No floor or ceiling effects[201,202] | Sensitive to change in moderate-severe[203] | No information found | Good[201,202,204] | No information found |
| Brief Psychiatric Rating Scale[205] | 3 (all published) | 190 | 20 minutes | Rated by an observer | May be validated for Alzheimer’s disease[206] | No information found | No information found | No information found | No information found | Good[206] | No information found |
| CERAD Behavioural Scale[207] | 1 (published) | 486 | 20-30 minutes | Semi-structured informant interview | Validated in French, Spanish, Arabic, Chinese and Japanese[151,208] | No information found | No information found | Some evidence of sensitivity[209] | No information found | Good[207] | Good[209] |
| Dysfunctional Behavior Rating Instrument[210] | 1 (published) | 406 | 20 minutes | Informant rated | Validated for PwD living in the community[210] | No information found | No information found | No information found | No information found | Good[210] | Good[211] |
| Neuropsychiatric inventory[212] | 38 (30 published and 8 ongoing) | 11,756 | 10-20 minutes | Informant interview | Validated across dementia severity[212]; Validated in Italian, Greek, Japanese, Korean, Mexican, Polish, Spanish, and Dutch[151,213] | MCID of 8 points in DOMINO trial[170] | No floor or ceiling effects[214] | Sensitive to change across dementia severities and types[214-217] | No information found | Good[212,213] | Good across three studies[212,213,218] |
| Nurses observation scale for geriatric patients[219] | 2 (all published) | 454 | 3-5 minutes | Nurses on wards normally rate with a caregiver | Validated for people with dementia in hospitals[220] | No information found | No information found | Good sensitivity to change in two studies including PwD[219,221] | 83% acceptability in mild-moderate dementia[220] | Good[220] | Good[220] |
| Plutchik Geriatric Rating Scale[222] | 1 (published) | 178 | 5-10 minutes | Rated by an observer | Doesn’t appear to be validated for use with people with dementia | No information found | No information found | No information found | No information found | No information found | No information found |
| Revised Memory and Behavior Problems Checklist[223] | 1 (published) | 168 | 10 minutes | Informant questionnaire | Validated in Taiwanese and Spanish[224,225] | No information found | No information found | May not be sensitive to detect progression of dementia in 1 study[226]; But appear sensitive to changes in another study[227] | No information found | No information found | Good[224] |

**TABLE F** Validation of quality of life outcomes

| **Measure** | **n of trials** | **n of participants** | **Time taken** | **Who completes** | **Relevant populations validated with** | **MCID** | **Ceiling & floor effects** | **Sensitivity to change** | **Acceptability** | **Inter-rater reliability** | **Test-retest reliability** |
| --- | --- | --- | --- | --- | --- | --- | --- | --- | --- | --- | --- |
| DEMQOL[228] | 4 (1 published and 3 ongoing) | 399 | 10-20 minutes | PwD and/or informant | Valid for mild to moderate dementia living in the community or residential care[228]; Validated in Spanish, German and Russian[229-231] | No information found | No ceiling or floor effect[228] | Sensitive to change in mild-moderate dementia[232-234] | Good[228] | Good inter-rater reliability of PWD and proxy versions for mild/moderate,[228] thought PwD rate higher than proxy in 1 study[235] and proxy rate higher than PwD in another study[234] | Good across studies including PwD[228,236] |
| European Quality of Life–5 Dimensions Scale[237] | 5 (4 published and 1 ongoing) | 4084 | 4-15 minutes for PWD + 2 minutes for proxy | PwD and/or informant | Validated for mild-moderate dementia living in the community or residential homes[238]; Available in 100 languages[239]; Validated in French[240] | No information found | No ceiling or floor effect observed in 1 study[240]; Substantial ceiling effect for patient ratings, but not proxy, in 2 studies[241,242] | Not sufficiently sensitive to detect changes in the progression of dementia[239] | High completion rate but acceptability decreases with dementia severity[239,240] | PwD provides significantly higher rating than proxy across 4 studies[239]; But in 1 study PWD and proxy ratings are similar for mild-moderate[238] | Patients’ test-retest ratings unreliable for mild to moderate and less reliable than carers ratings[239] but 2 studies[240,241] report good test-retest reliability |
| Quality of Life in Alzheimer’s Disease Scale[243] | 8 (6 published and 2 ongoing) | 3341 | 5 minutes for informant version + 10-15 minutes for PWD | PwD and/or informant | Validated for people living in the community or residential care[244]; Validated for use in MMSE scores over 10[245]; Validated in English, French, Portuguese, Spanish, Japanese, Cantonese, Mandarin, Korean, Danish, Swedish, German and Greek[151,245] | One standard deviation[246] | No ceiling or floor effect observed[247]; Minimal ceiling and floor effects across 8 studies[4,248-254] | Sensitive to change in studies of mild-moderate dementia[244] | Good[247] | Mixed inter-rater reliability of PWD and informant responses across 8 studies[243,244,255-261] | Good[243,244] |

**TABLE G** Validation of activities of daily living outcomes

| **Measure** | **n of trials** | **n of participants** | **Time taken** | **Who completes** | **Relevant populations validated with** | **MCID** | **Ceiling & floor effects** | **Sensitivity to change** | **Acceptability** | **Inter-rater reliability** | **Test-retest reliability** |
| --- | --- | --- | --- | --- | --- | --- | --- | --- | --- | --- | --- |
| Alzheimer's Disease Co-operative Study - Activities of Daily Living[262] | 34 (28 published and 6 ongoing) | 11,500 | 15-20 minutes | Informant rated | Validated for use in Spain, Sweden, Latvia, and Bosnia and Hercegovina[151]; Validated for mild to moderate living in the community[263] | No information found | No information found | Sensitive to change in mild-moderate dementia in three studies[264-266] but not in one[267] | No information found | No information found | Good across 4 studies[262,265,268,269] |
| Alzheimer's Disease Functional Assessment and Change Scale[262] | 2 (2 published) | 350 | 15-20 minutes | Informant rated | Validated in Spanish[270] | No information found | No information found | No information found | No information found | No information found | No information found |
| Bristol Activities of Daily Living Scale[271] | 5 (3 published and 2 ongoing) | 1117 | 15 minutes | Informant rated | Validated for PwD living in community[272] | Minimum clinically important difference of 3.5 points in DOMINO trial[170] | No information found | Sensitive to change in dementia[221,272] | Carers report it’s easy to complete[271] | Mixed across studies[273] | Good[271,273] |
| Dependence scale[274] | 3 (all published) | 3343 | 15-20 minutes | Informant rated | Valid for use with PwD living in the community[274] | No information found | Floor effect on cognition subscale[275] | Sensitive to dementia progression[274] but may not pick up small changes in clinical trials[276] | No information found | Good[31] | Good[274] |
| Disability Assessment For Dementia[277] | 13 (11 published, 1 protocol and 1 ongoing) | 2914 | 15 minutes | Informant rated | Validated in Korean, Chinese, Italian, Spanish, Persian, Portuguese, and Turkish[278-284]; Validated across dementia severities living in the community[279] | No information found | No floor or ceiling effects[277,285] | Good sensitivity to change in 6 studies[286-291] but one study[292] found it wasn't sensitive to change in comparison to other measures | No information found | Good[277] | Good[277] |
| Functional Activities Questionnaire[293] | 2 (ongoing trials only) | N/A | 10 minutes | Informant interview | Validated for mild dementia[294] | No information found | No information found | No information found | No information found | Good[293] | No information found |
| Interview for deterioration in Daily Living Activities in Dementia[295] | 2 (published trials only) | 219 | 15 minutes | Informant rated | Validated for mild dementia living at home[296]; Validated in Spanish and Dutch[70,111,297] | No information found | No ceiling or floor effect[298] | Thought to be responsive to change[275] | No information found | Good[296] | Good[275] |
| Katz Index of Activities of Daily Living Scale[299] | 3 (2 published 1 protocol) | 185 | 10 minutes | Informant rated | Validated as more of a clinical assessment than a measure of treatment effectiveness[300] | No information found | No information found | Sensitive to dementia progression [301,302] but may not be sensitive to small changes[303] | No information found | Good[303,304] | Good[303] |
| Lawton Instrumental Activities of Daily Living Scale[305] | 8 (7 published and 1 protocol) | 1125 | 10 minutes | Clinician/ researcher rated | Validated in Asian older adults living in the community (some with dementia)[306] | No information found | Ceiling effect reported[307] | Sensitive to treatment effects in moderate-severe[308] | No information found | Good[309] | Good[309] |
| Nuremberg Gerontopsychological Rating Scale for Activities of Daily Living[310] | 3 (all published) | 530 | Not specified | Self-complete or informant questionnaire | Doesn’t appear to be validated for use with people with dementia | No information found | No information found | May not be sensitive to change over time[311] | No information found | Family carers rate more deficits than paid carers[311] | No information found |
| Physical self-maintenance scale[305] | 3 (all published) | 429 | 5 minutes | Self-complete or observer rated | Validated for PwD living in the community[304] | No information found | Ceiling effect likely in PwD living in community[275] | Sensitive to treatment effects in moderate-severe[308] | No information found | Good[309] | Good[309] |
| Video recorder home-behavioural assessment[35] | 1 (published) | 48 | Not specified | Researcher rated | Doesn’t appear to be validated for use with people with dementia | No information found | No information found | May be sensitive to change[35] | No information found | Good[35] | Good[35] |

**TABLE H** Validation of global outcomes

| **Measure** | **n of trials** | **n of participants** | **Time taken** | **Who completes** | **Relevant populations validated with** | **MCID** | **Ceiling & floor effects** | **Sensitivity to change** | **Acceptability** | **Inter-rater reliability** | **Test-retest reliability** |
| --- | --- | --- | --- | --- | --- | --- | --- | --- | --- | --- | --- |
| *Impression of change scales:* | |  |  |  |  |  |  |  |  |  |  |
| Alzheimer's Disease Cooperative Study - Clinical Global Impression of Change[312] | 8 (7 published and 1 ongoing) | 1590 | 20 minutes | PwD + informant | Validated for use in clinical and home settings[313] | No information found | No information found | Sensitive to detect changes in mild to moderate dementia over 12 months[312] | No information found | Good[312] | Good[312] |
| Clinical Global Impression’s Scale[314] | 15 (14 published and 1 ongoing) | 2063 | 5 minutes | Clinician rated by interview with PWD/informant | Doesn’t appear to be validated for use with people with dementia | No information found | No information found | No information found | No information found | No information found | No information found |
| Clinician's Interview-Based Impression of Change Plus Caregiver Input[315] | 12 (12 published) | 4087 | 10-40 minutes | Clinician semi-structured interview with PWD + informant | Validated in Japanese[316] | No information found | No information found | Sensitive to change in dementia treatment studies[312,317] | Good acceptability[317] | Good[318] | Good[317] |
| *Multiple domain scales:* | |  |  |  |  |  |  |  |  |  |  |
| Short CAMDEX[319] | 1 (published) | 321 | 30 minutes | Clinician | Used to screen for and diagnose dementia[319] | No information found | No information found | No information found | No information found | No information found | No information found |
| Blessed Dementia rating scale[320] | 3 (3 published) | 190 | 15 minutes | Informant rated | Validated in Taiwanese, Chinese, Korean, and Czech[321-324] | No information found | Floor and ceiling effects in 2 studies[320,325] | Sensitive to progression of dementia [326] | No information found | Good in 2 studies[274,327] | Good in 3 studies[326,328,329] |
| Dementia Severity Rating Scale[330] | 3 (2 published and 1 ongoing) | 164 | 5 minutes | Carer questionnaire | Validated for mild to severe dementia[331] | No information found | No floor or ceiling effects[330] | Sensitive to change across mild-severe[330-332] | No information found | Good inter-rater reliability of caregiver responses compared to clinician information[330] | Good[330,333] |
| Gottfries-Brane-Steen rating scale for dementia[334] | 4 (4 published) | 636 | 20-30 minutes | Clinician interview with PwD + informant interview | Translated in Czech, Danish, Italian, Japanese, Norwegian, Spanish and Swedish[335]; Validated across dementia severities[335] | No information found | Can have a ceiling effect in mild dementia[336] | Sensitive to change across dementia severities[335] | No information found | Good across 9 studies[335] | No information found |
| Sandoz Clinical Assessment-Geriatric Scale[337] | 2 (2 published) | 298 | 15-30 minutes | Clinician observation | Validated in French and German[151] | No information found | No information found | Sensitive to change across 3 studies[337-339] | No information found | Good in 2 studies[337,338] | Low across 3 studies [328,338,340] |
| *Staging of dementia scales:* | |  |  |  |  |  |  |  |  |  |  |
| Clinical Dementia Rating[341] | 48 (34 published, 1 protocol, and 13 ongoing) | 14,596 | 40 minutes | PwD + carer | Valid for mild-severe dementia[342]; Valid in community and residential care settings[151]; Available in Chinese, Czech, Dutch, English, Finnish, French, German, Hebrew, Polish, Spanish, Swedish, Portugese[151,343,344] | No information found | Minimal floor and ceiling effects across 11 studies[345-355]; Floor and ceiling effects in 1 study[356] | Sensitive to treatment effects across 12 studies[345-355,357,358] | No information found | Good to very good across 12 studies [341,356,359-368] | Good[364] |
| Global Deterioration Scale | 6 (6 published) | 809 | 2 minutes | Informant rated | English version has been translated and validated in German and Korean[151,369]; Validated in community or residential care[370] | No information found | No information found | Good sensitivity to change in 2 studies[371,372] but not in another study[373] | No information found | Good across 4 studies[206,374-376] | Good[374] |

**TABLE I** Validation of biological markers outcomes

| **Type of biological technique** | **n of trials** | **n of participants** | **Type of biological marker in trials** | **Accuracy** | **Sensitivity to change** | **Risks** |
| --- | --- | --- | --- | --- | --- | --- |
| MRI | 30 (16 published, 1 protocol, and 13 ongoing) | 4788 | Mostly serial structural MRI for volume (22 trials; 4788 participants). The other 8 trials used MRI for: Perfusion (1 trial); Amyloid (2 trials); Changes in brain network functional and structural connectivity and perfusion (1 trial); Brain functional connectivity (1 trial); Quantitation of lacunar lesions and diffuse white matter lesions (1 trial); Whole brain atrophy, white matter hyperintensity volume and cerebral blood flow (1 trial); Unknown (1 trial) | Serial structural MRI: Gives accurate measurements of hippocampal volume and correlates with neuronal numbers[377] | Serial structural MRI: | - Strong magnetic field so not appropriate for anyone who has metallic impalnts inside their body e.g pacemaker[388] |
|  |  |  |  |  | - Hippocampal atrophy correlates with AD pathology[378] including with braak staging of dementia, tau, Amyloid beta burden in people with AD[379] |  |
|  |  |  |  |  | - There is consistent replication of hippocampal atrophy in assessing neurodegeneration caused by AD across disease severity, including evidence from 11 different research groups[379] | - Can be noisy so need appropriate hearing protection[388] |
|  |  |  |  |  | - Atrophy in the entorhinal cortex, ventricle, and particularly the hippocampus and whole brain, accelerates with increasing cognitive decline across dementia[378,380-384] | - Short term exposure causes no harmful biological effects[389] |
|  |  |  |  |  | - Structural MRI more sensitive to change across AD severities than amyloid markers measured via PET imaging or CSF[385-387] | - Can have psychological risks e.g. anxiety and claustrophobia[390,391] |

**References within the tables**

1. Aisen PS (2000) Anti-inflammatory therapy for Alzheimer's disease: implications of the prednisone trial. Acta Neurol Scand 176: 85-89.

2. Aisen PS, Davis K, Berg J, Schafer K, Campbell K, et al. (2000) A randomized controlled trial of prednisone in Alzheimer's disease. Neurology 54: 588-593.

3. Aisen PS, Schmeidler J, Pasinetti GM (2002) Randomized pilot study of nimesulide treatment in Alzheimer's disease. Neurology 58: 1050-1054.

4. Aisen PS, Schafer KA, Grundman M, Pfeiffer E, Sano M, et al. (2003) Effects of rofecoxib or naproxen vs placebo on Alzheimer disease progression: a randomized controlled trial. JAMA 289: 2819-2826.

5. Aisen PS, Saumier D, Briand R, Laurin J, Gervais F, et al. (2006) A Phase II study targeting amyloid-beta with 3APS in mild-to-moderate Alzheimer disease. Neurology 67: 1757-1763.

6. Aisen PS, Gauthier S, Vellas B, Briand R, Saumier D, et al. (2007) Alzhemed: A potential treatment for Alzheimer's disease. Curr Alzheimer Res 4: 473-478.

7. National IoA, and the General Clinical Research Center Programme (2008) High-dose vitamin B does not slow cognitive decline in AD. Brown University Geriatric Psychopharmacology Update 12: 3-3 1p.

8. Aisen PS, Schneider LS, Sano M, Diaz-Arrastia R, Dyck CH, et al. (2008) High-dose B vitamin supplementation and cognitive decline in Alzheimer disease: a randomized controlled trial. JAMA 300: 1774-1783.

9. Viswanathan A (2009) High-dose B vitamin supplementation as a disease-modifying therapy in alzheimer disease. Arch Neurol 66: 520-522.

10. Saumier D, Duong A, Haine D, Garceau D, Sampalis J (2009) Domain-specific cognitive effects of tramiprosate in patients with mild to moderate Alzheimer's disease: ADAS-cog subscale results from the Alphase Study. J Nutr Health Aging 13: 808-812.

11. Gauthier S, Aisen PS, Ferris SH, Saumier D, Duong A, et al. (2009) Effect of tramiprosate in patients with mild-to-moderate Alzheimer's disease: exploratory analyses of the MRI sub-group of the Alphase study. J Nutr Health Aging 13: 550-557.

12. Aisen PS, Gauthier S, Ferris SH, Saumier D, Haine D, et al. (2011) Tramiprosate in mild-to-moderate Alzheimers disease -- a randomized, double-blind, placebo-controlled, multi-centre study (the Alphase Study). Arch Med Sci 7: 102-111 110p.

13. Akhondzadeh S, Sabet MS, Harirchian MH, Togha M, Cheraghmakani H, et al. (2010) A 22-week, multicenter, randomized, double-blind controlled trial of Crocus sativus in the treatment of mild-to-moderate Alzheimer's disease. Psychopharmacol 207: 637-643.

14. Akhondzadeh S, Shafiee-Sabet M, Harirchian MH, Togha M, Cheraghmakani H, et al. (2010) Saffron in the treatment of patients with mild to moderate Alzheimer’s disease: a 16-week, randomized and placebo-controlled trial. J Clin Pharm Ther 35: 581-588 588p.

15. Alvarez XA, Pichel V, Perez P, Laredo M, Corzo D, et al. (2000) Double-blind, randomized, placebo-controlled pilot study with anapsos in senile dementia: Effects on cognition, brain bioelectrical activity and cerebral hemodynamics. Methods Find Exp Clin Pharmacol 22: 585-594.

16. Alvarez XA, Cacabelos R, Laredo M, Couceiro V, Sampedro C, et al. (2006) A 24-week, double-blind, placebo-controlled study of three dosages of Cerebrolysin in patients with mild to moderate Alzheimer's disease. Eur J Neurol 13: 43-54.

17. Alvarez XA, Cacabelos R, Sampedro C, Aleixandre M, Linares C, et al. (2011) Efficacy and safety of Cerebrolysin in moderate to moderately severe Alzheimer's disease: results of a randomized, double-blind, controlled trial investigating three dosages of Cerebrolysin. Eur J Neurol 18: 59-68.

18. Asthana S, Craft S, Baker LD, Raskind MA, Birnbaum RS, et al. (1999) Cognitive and neuroendocrine response to transdermal estrogen in postmenopausal women with Alzheimer's disease: results of a placebo-controlled, double-blind, pilot study. Psychoneuroendocrinology 24: 657-677.

19. Pasqualetti P, Bonomini C, Dal Forno G, Paulon L, Sinforiani E, et al. (2009) A randomized controlled study on effects of ibuprofen on cognitive progression of Alzheimer's disease. Aging Clin Exp Res 21: 102-110.

20. Babiloni C, Frisoni, G. B., Del Percio, C., Zanetti, O., Bonomini, C., Cassetta, E., et al. (2009) Ibuprofen treatment modifies cortical sources of EEG rhythms in mild Alzheimer’s disease. Clin Neurophysiol 120: 709-718.

21. Bae C-y, Cho C-Y, Cho K, Oh BH, Choi KG, et al. (2000) A double-blind, placebo-controlled, multicenter study of Cerebrolysin for Alzheimer's disease. J Am Geriatr Soc 48: 1566-1571.

22. Ban TA, Morey L, Aguglia E, Azzarelli O, Balsano F, et al. (1990) Nimodipine in the treatment of old age dementias. Prog Neuropsychopharmacol Biol Psychiatry 14: 525-551.

23. Bayer AJ, Bullock R, Jones RW, Wilkinson D, Paterson KR, et al. (2005) Evaluation of the safety and immunogenicity of synthetic Abeta42 (AN1792) in patients with AD. Neurology 64: 94-101.

24. Holmes C, Boche D, Wilkinson D, Yadegarfar G, Hopkins V, et al. (2008) Long-term effects of Abeta42 immunisation in Alzheimer's disease: follow-up of a randomised, placebo-controlled phase I trial. Lancet 372: 216-223.

25. Bentham P, Gray R, Sellwood E, Hills R, Crome P, et al. (2008) Aspirin in Alzheimer's disease (AD2000): a randomised open-label trial. Lancet Neurol 7: 41-49.

26. Bilikiewicz A, Gaus W (2004) Colostrinin (a naturally occurring, proline-rich, polypeptide mixture) in the treatment of Alzheimer's disease. J Alzheimers Dis 6: 17-26.

27. Black RS, Sperling RA, Safirstein B, Motter RN, Pallay A, et al. (2010) A single ascending dose study of bapineuzumab in patients with alzheimer disease. Alzheimer Dis Assoc Disord 24: 198-203.

28. Rinne JO, Brooks DJ, Rossor MN, Fox NC, Bullock R, et al. (2010) 11C-PiB PET assessment of change in fibrillar amyloid-beta load in patients with Alzheimer's disease treated with bapineuzumab: A phase 2, double-blind, placebo-controlled, ascending-dose study. Lancet Neurol 9: 363-372.

29. Blennow K, Zetterberg H, Rinne JO, Salloway S, Wei J, et al. (2012) Effect of immunotherapy with bapineuzumab on cerebrospinal fluid biomarker levels in patients with mild to moderate Alzheimer disease. Arch Neurol 69: 1002-1010.

30. Salloway S, Sperling R, Gilman S, Fox NC, Blennow K, et al. (2009) A phase 2 multiple ascending dose trial of bapineuzumab in mild to moderate Alzheimer disease. Neurology 73: 2061-2070.

31. Bowen RL, Perry G, Xiong C, Smith MA, Atwood CS (2015) A clinical study of lupron depot in the treatment of women with Alzheimer's disease: preservation of cognitive function in patients taking an acetylcholinesterase inhibitor and treated with high dose lupron over 48 weeks. J Alzheimers Dis 44: 549-560.

32. Claxton A, Baker LD, Hanson A, Trittschuh EH, Cholerton B, et al. (2015) Long-acting intranasal insulin detemir improves cognition for adults with mild cognitive impairment or early-stage Alzheimer's disease dementia. J Alzheimers Dis 44: 897-906.

33. Craft S, Baker LD, Montine TJ, Minoshima S, Watson G, et al. (2012) Intranasal insulin therapy for Alzheimer disease and amnestic mild cognitive impairment: A pilot clinical trial. Arch Neurol 69: 29-38.

34. Crapper McLachlan DR, Smith WL, Kruck TP (1993) Desferrioxamine and Alzheimer's disease: video home behavior assessment of clinical course and measures of brain aluminum. Ther Drug Monit 15: 602-607.

35. Crapper McLachlan DR, Dalton AJ, Kruck TP, Bell MY, Smith WL, et al. (1991) Intramuscular desferrioxamine in patients with Alzheimer's disease. Lancet 337: 1304-1308.

36. Cucinotta D, De Leo D, Frattola L, Trabucchi M, Albizatti MG, et al. (1998) Dihydroergokryptine as long-term treatment of Alzheimer type dementia: A multicenter two-year follow-up. Arch Gerontol Geriatr 27: 103-110.

37. Dodel R, Rominger A, Bartenstein P, Barkhof F, Blennow K, et al. (2013) Intravenous immunoglobulin for treatment of mild-to-moderate Alzheimer's disease: A phase 2, randomised, double-blind, placebo-controlled, dose-finding trial. Lancet Neurol 12: 233-243.

38. Doody RS, Gavrilova SI, Sano M, Thomas RG, Aisen PS, et al. (2008) Effect of dimebon on cognition, activities of daily living, behaviour, and global function in patients with mild-to-moderate Alzheimer's disease: a randomised, double-blind, placebo-controlled study. Lancet 372: 207-215.

39. Doody RS, Raman R, Sperling RA, Seimers E, Sethuraman G, et al. (2015) Peripheral and central effects of gamma-secretase inhibition by semagacestat in Alzheimer's disease. Alzheimers Res Ther 7: 36.

40. Doody RS, Raman R, Farlow M, Iwatsubo T, Vellas B, et al. (2013) A phase 3 trial of semagacestat for treatment of Alzheimer's disease. N Engl J Med 369: 341-350.

41. Liu-Seifert H, Siemers E, Holdridge KC, Andersen SW, Lipkovich I, et al. (2015) Delayed-start analysis: Mild Alzheimer's disease patients in solanezumab trials, 3.5 years. Alzheimer's and Dementia: Translational Research and Clinical Interventions 1: 111-121.

42. Doody RS, Thomas RG, Farlow M, Iwatsubo T, Vellas B, et al. (2014) Phase 3 trials of solanezumab for mild-to-moderate Alzheimer's disease. N Engl J Med 370: 311-321.

43. Endres K, Fahrenholz F, Lotz J, Hiemke C, Teipel S, et al. (2014) Increased CSF APPs-a levels in patients with Alzheimer disease treated with acitretin. Neurology 83: 1930-1935.

44. Farlow M, Arnold SE, van Dyck CH, Aisen PS, Snider BJ, et al. (2012) Safety and biomarker effects of solanezumab in patients with Alzheimer's disease. Alzheimers Dement 8: 261-271.

45. Lannfelt L, Blennow K, Zetterberg H, Batsman S, Ames D, et al. (2008) Safety, efficacy, and biomarker findings of PBT2 in targeting Abeta as a modifying therapy for Alzheimer's disease: a phase IIa, double-blind, randomised, placebo-controlled trial. Lancet Neurol 7: 779-786.

46. Faux NG, Ritchie CW, Gunn A, Rembach A, Tsatsanis A, et al. (2010) PBT2 rapidly improves cognition in Alzheimer's Disease: additional phase II analyses. J Alzheimers Dis 20: 509-516.

47. Faxen-Irving G, Freund-Levi Y, Eriksdotter-Jonhagen M, Basun H, Hjorth E, et al. (2013) Effects on transthyretin in plasma and cerebrospinal fluid by DHA-rich n - 3 fatty acid supplementation in patients with Alzheimer's disease: the OmegAD study. J Alzheimers Dis 36: 1-6.

48. Freund-Levi Y, Eriksdotter-Jönhagen M, Cederholm T, Basun H, Faxén-Irving G, et al. (2006) Omega-3 fatty acid treatment in 174 patients with mild to moderate Alzheimer disease: OmegAD study: a randomized double-blind trial. Arch Neurol 63: 1402-1408.

49. Ferrari E, Cucinotta D, Albizatti M, Bartorelli L, Colombo N, et al. (1998) Effectiveness and safety of posatirelin in the treatment of senile dementia: A multicenter, double-blind, placebo-controlled study. Arch Gerontol Geriatr 6: 163-174.

50. Fleisher AS, Raman R, Siemers ER, Becerra L, Clark CM, et al. (2008) Phase 2 safety trial targeting amyloid beta production with a gamma-secretase inhibitor in Alzheimer disease. Arch Neurol 65: 1031-1038.

51. Fleisher AS, Truran D, Mai JT, Langbaum JBS, Aisen PS, et al. (2011) Chronic divalproex sodium use and brain atrophy in Alzheimer disease. Neurology 77: 1263-1271.

52. Hock C, Konietzko U, Streffer JR, Tracy J, Signorell A, et al. (2003) Antibodies against beta-amyloid slow cognitive decline in Alzheimer's disease. Neuron 38: 547-554.

53. Orgogozo JM, Gilman S, Dartigues JF, Laurent B, Puel M, et al. (2003) Subacute meningoencephalitis in a subset of patients with AD after Abeta42 immunization. Neurology 61: 46-54.

54. Koepsell TD, Chi YY, Zhou XH, Lee WW, Ramos EM, et al. (2007) An alternative method for estimating efficacy of the AN1792 vaccine for Alzheimer disease. Neurology 69: 1868-1872.

55. Gilman S, Koller M, Black RS, Jenkins L, Griffith SG, et al. (2005) Clinical effects of Abeta immunization (AN1792) in patients with AD in an interrupted trial. Neurology 64: 1553-1562.

56. Fox N, Black R, Gilman S, Rossor M, Griffith S, et al. (2005) Effects of Abeta immunization (AN1792) on MRI measures of cerebral volume in Alzheimer disease. Neurology 64: 1563-1572.

57. Vellas B, Black R, Thal LJ, Fox NC, Daniels M, et al. (2009) Long-term follow-up of patients immunized with AN1792: reduced functional decline in antibody responders. Curr Alzheimer Res 6: 144-151.

58. Galasko DR, Peskind E, Clark CM, Quinn JF, Ringman JM, et al. (2012) Antioxidants for Alzheimer disease: a randomized clinical trial with cerebrospinal fluid biomarker measures. Arch Neurol 69: 836-841.

59. Galasko D, Bell J, Mancuso JY, Kupiec JW, Sabbagh MN, et al. (2014) Clinical trial of an inhibitor of RAGE-Abeta interactions in Alzheimer disease. Neurology 82: 1536-1542.

60. Gauthier S, Rountree S, Finn B, LaPlante B, Weber E, et al. (2015) Effects of the Acetylcholine Release Agent ST101 with Donepezil in Alzheimer's Disease: A Randomized Phase 2 Study. J Alzheimers Dis 48: 473-481.

61. Geldmacher DS, Fritsch T, McClendon MJ, Landreth G (2011) A randomized pilot clinical trial of the safety of pioglitazone in treatment of patients with Alzheimer disease. Arch Neurol 68: 45-50.

62. Gold M, Alderton C, Zvartau-Hind M, Egginton S, Saunders AM, et al. (2010) Rosiglitazone monotherapy in mild-to-moderate Alzheimer's disease: results from a randomized, double-blind, placebo-controlled phase III study. Dement Geriatr Cogn Disord 30: 131-146 116p.

63. Green RC, Schneider LS, Amato DA, Beelen AP, Wilcock G, et al. (2009) Effect of tarenflurbil on cognitive decline and activities of daily living in patients with mild Alzheimer disease: a randomized controlled trial. JAMA 302: 2557-2564.

64. Myriad P (2010) No significant effect of tarenflurbil on cognition in early Alzheimer's disease. Brown University Geriatric Psychopharmacology Update 14: 1-6 3p.

65. Grimaldi LM, Zappala G, Iemolo F, Castellano AE, Ruggieri S, et al. (2014) A pilot study on the use of interferon beta-1a in early Alzheimer's disease subjects. J Neuroinflammation 11: 30.

66. Hampel H, Ewers M, Burger K, Annas P, Mortberg A, et al. (2009) Lithium trial in Alzheimer's disease: a randomized, single-blind, placebo-controlled, multicenter 10-week study. J Clin Psychiatry 70: 922-931.

67. Hock C, Maddalena A, Heuser I, Naber D, Oertel W, et al. (2000) Treatment with the selective muscarinic agonist talsaclidine decreases cerebrospinal fluid levels of total amyloid beta-peptide in patients with Alzheimer's disease. Ann N Y Acad Sci 920: 285-291.

68. Hock C, Maddalena A, Raschig A, Muller-Spahn F, Eschweiler G, et al. (2003) Treatment with the selective muscarinic m1 agonist talsaclidine decreases cerebrospinal fluid levels of A beta 42 in patients with Alzheimer's disease. Amyloid 10: 1-6.

69. Jhee SS, Frackiewicz EJ, Tolbert D, Sainati S, Karim A, et al. (2004) A pharmacokinetic, pharmacodynamic, and safety study of celecoxib in subjects with probable Alzheimer's disease. Clinical research and regulatory affairs 21: 49-66.

70. Jong D, Jansen R, Hoefnagels W, Jellesma-Eggenkamp M, Verbeek M, et al. (2008) No effect of one-year treatment with indomethacin on Alzheimer's disease progression: a randomized controlled trial. PloS one 3: e1475.

71. Kadir A, Andreasen N, Almkvist O, Wall A, Forsberg A, et al. (2008) Effect of phenserine treatment on brain functional activity and amyloid in Alzheimer's disease. Ann Neurol 63: 621-631.

72. Kessler H, Pajonk FG, Bach D, Schneider-Axmann T, Falkai P, et al. (2008) Effect of copper intake on CSF parameters in patients with mild Alzheimer's disease: a pilot phase 2 clinical trial. J Neural Transm 115: 1651-1659.

73. Kessler H, Bayer TA, Bach D, Schneider-Axmann T, Supprian T, et al. (2008) Intake of copper has no effect on cognition in patients with mild Alzheimer's disease: a pilot phase 2 clinical trial. J Neural Transm 115: 1181-1187.

74. Landen JW, Zhao Q, Cohen S, Borrie M, Woodward M, et al. (2013) Safety and pharmacology of a single intravenous dose of ponezumab in subjects with mild-to-moderate alzheimer disease: A phase I, randomized, placebo-controlled, double-blind, dose-escalation study. Clin Neuropharmacol 36: 14-23.

75. Leszek J, Inglot AD, Janusz M, Lisowski J, Krukowska K, et al. (1999) Colostrinin: a proline-rich polypeptide (PRP) complex isolated from ovine colostrum for treatment of Alzheimer's disease. A double-blind, placebo-controlled study. Arch Immunol Ther Exp 47: 377-385.

76. Li N (2015) Neuroprotective Effects of Cistanches Herba Therapy on Patients with Moderate Alzheimer’s Disease. Evid Based Complement Alternat Med 2015: 1-12 12p.

77. Lovestone S, Boadab M, Dubois B, Hull M, Rinne JO, et al. (2015) A phase II trial of tideglusib in Alzheimer's disease. J Alzheimers Dis 45: 75-88.

78. Maher-Edwards G, De'Ath J, Barnett C, Lavrov A, Lockhart A (2015) A 24-week study to evaluate the effect of rilapladib on cognition and cerebrospinal fluid biomarkers of Alzheimer's disease. Alzheimer's and Dementia: Translational Research and Clinical Interventions 1: 131-140.

79. Marcusson J, Rother M, Kittner B, Rossner M, Smith R, et al. (1997) A 12-month, randomized placebo-controlled trial of propentofylline (HWA 285) in patients with dementia according to DSM III-R. Dement Geriatr Cogn Disord 8: 320-328.

80. Molloy D, Standish T, Zhou Q, Guyatt G (2013) A multicenter, blinded, randomized, factorial controlled trial of doxycycline and rifampin for treatment of Alzheimer's disease: The DARAD trial. Int J Geriatr Psychiatry 28: 463-470.

81. Muresanu DF, Rainer M, Moessler H (2002) Improved global function and activities of daily living in patients with AD: a placebo-controlled clinical study with the neurotrophic agent Cerebrolysin. J Neural Transm Supplementum.: 277-285.

82. Muresanu DF, Alvarez X, Moessler H, Buia M, Stan A, et al. (2008) A pilot study to evaluate the effects of Cerebrolysin on cognition and qEEG in vascular dementia: Cognitive improvement correlates with qEEG acceleration. J Neurol Sci 267: 112-119.

83. Nygaard HB, Wagner AF, Bowen GS, Good SP, MacAvoy MG, et al. (2015) A phase Ib multiple ascending dose study of the safety, tolerability, and central nervous system availability of AZD0530 (saracatinib) in Alzheimer's disease. Alzheimers Res Ther 7: 35.

84. Ostrowitzki S, Deptula D, Thurfjell L, Barkhof F, Bohrmann B, et al. (2012) Mechanism of amyloid removal in patients with Alzheimer disease treated with gantenerumab. Arch Neurol 69: 198-207.

85. Quinn JF, Raman R, Thomas RG, Yurko-Mauro K, Nelson EB, et al. (2010) Docosahexaenoic acid supplementation and cognitive decline in Alzheimer disease: A randomized trial. JAMA 304: 1903-1911.

86. Regland B, Lehmann W, Abedini I, Blennow K, Jonsson M, et al. (2001) Treatment of Alzheimer's disease with clioquinol. Dement Geriatr Cogn Disord 12: 408-414.

87. Reines S, Block G, Morris J, Liu G, Nessly M, et al. (2004) Rofecoxib: No effect on Alzheimer's disease in a 1-year, randomized, blinded, controlled study. Neurology 62: 66-71.

88. Ringman JM, Frautschy SA, Teng E, Begum AN, Bardens J, et al. (2012) Oral curcumin for Alzheimer's disease: tolerability and efficacy in a 24-week randomized, double blind, placebo-controlled study. Alzheimers Res Ther 4: 43.

89. Ritchie C, Bush AI, Mackinnon A, Macfarlane S, Mastwyk M, MacGregor L, et al. (2003) Metal-protein attenuation with iodochlorhydroxyquin (clioquinol) targeting Abeta amyloid deposition and toxicity in Alzheimer disease: a pilot phase 2 clinical trial. Arch Neurol 60: 1685-1691.

90. Ruether E, Ritter R, Apecechea M, Freytag S, Windisch M (1994) Efficacy of the peptidergic nootropic drug cerebrolysin in patients with senile dementia of the Alzheimer type (SDAT). Pharmacopsychiatry 27: 32-40.

91. Ruether E, Ritter R, Apecechea M, Freytag S, Gmeinbauer R, et al. (2000) Sustained improvements in patients with dementia of Alzheimer's type (DAT) 6 months after termination of Cerebrolysin therapy. J Neural Transm 107: 815-829.

92. Ruether E, Husmann R, Kinzler E, Diabl E, Klingler D, et al. (2001) A 28-week, double-blind, placebo-controlled study with Cerebrolysin in patients with mild to moderate Alzheimer's disease. Int Clin Psychopharm 16: 253-263.

93. Ruether E, Alvarez XA, Rainer M, Moessler H (2002) Sustained improvement of cognition and global function in patients with moderately severe Alzheimer's disease: a double-blind, placebo-controlled study with the neurotrophic agent Cerebrolysin. J Neural Transm Supplementum.: 265-275.

94. Salloway S, Sperling R, Keren R, Porsteinsson AP, van Dyck CH, et al. (2011) A phase 2 randomized trial of ELND005, scyllo-inositol, in mild to moderate Alzheimer disease. Neurology 77: 1253-1262.

95. Salloway S, Sperling R, Fox NC, Blennow K, Klunk W, et al. (2014) Two phase 3 trials of bapineuzumab in mild-to-moderate Alzheimer's disease. N Engl J Med 370: 322-333.

96. Sano M, Ernesto C, Klauber MR, Schafer K, Woodbury P, et al. (1996) Rationale and design of a multicenter study of selegiline and alpha-tocopherol in the treatment of Alzheimer disease using novel clinical outcomes. Alzheimer's Disease Cooperative Study. Alzheimer Dis Assoc Disord 10: 132-140.

97. Sano M, Bell K, Galasko D, Galvin J, Thomas R, et al. (2011) A randomized, double-blind, placebo-controlled trial of simvastatin to treat Alzheimer disease. Neurology 77: 556-563.

98. Scharf S, Mander A, Ugoni A, Vajda F, Christophidis N (1999) A double-blind, placebo-controlled trial of diclofenac/misoprostol in Alzheimer's disease. Neurology 53: 197-201.

99. Schwam EM, Nicholas T, Chew R, Billing CB, Davidson W, et al. (2014) A multicenter, double-blind, placebo-controlled trial of the PDE9A inhibitor, PF-04447943, in Alzheimer's disease. Curr Alzheimer Res 11: 413-421.

100. Ser T, Steinwachs KC, Gertz HJ, Andrés MV, Gómez-Carrillo B, et al. (2013) Treatment of Alzheimer's disease with the GSK-3 inhibitor tideglusib: a pilot study. J Alzheimers Dis 33: 205-215.

101. Sevigny JJ, Ryan JM, Dyck CH, Peng Y, Lines CR, et al. (2008) Growth hormone secretagogue MK-677: no clinical effect on AD progression in a randomized trial. Neurology 71: 1702-1708.

102. Siemers ER, Friedrich, S., Dean, R. A., Gonzales, C. R., Farlow, M. R., Paul, S. M., et al. (2010) Safety and changes in plasma and cerebrospinal fluid amyloid β after a single administration of an amyloid β monoclonal antibody in subjects with Alzheimer disease. Clin Neuropharmacol 32: 67-33.

103. Silverberg GD, Levinthal E, Sullivan EV, Bloch DA, Chang SD, et al. (2002) Assessment of low-flow CSF drainage as a treatment for AD: Results of a randomized pilot study. Neurology 59: 1139-1145.

104. Silverberg G, Mayo M, Saul T, Fellmann J, Carvalho J, et al. (2008) Continuous CSF drainage in AD: Results of a double-blind, randomized, placebo-controlled study. Neurology 71: 202-209.

105. Simons M, Schwärzler, F., Lütjohann, D., Von Bergmann, K., Beyreuther, K., Dichgans, J., et al. (2002) Treatment with simvastatin in normocholesterolemic patients with Alzheimer's disease: A 26-week randomized, placebo-controlled, double-blind trial. Ann Neurol 52: 346-350.

106. Soininen H, West C, Robbins J, Niculescu L (2006) Long-term efficacy and safety of celecoxib in Alzheimer's Disease. Dement Geriatr Cogn Disord 23: 8-21 14p.

107. Sparks DL, Sabbagh MN, Connor DJ, Lopez J, Launer LJ, et al. (2005) Atorvastatin for the treatment of mild to moderate Alzheimer disease: preliminary results. Arch Neurol 62: 753-757.

108. Sweetlove M (2012) Phase III CONCERT trial of latrepirdine: Negative results. Pharmaceutical medicine 26: 113-115.

109. Tan RS, Pu SJ (2003) A pilot study on the effects of testosterone in hypogonadal aging male patients with Alzheimer's disease. Aging Male 6: 13-17.

110. Turner RS, Thomas RG, Craft S, van Dyck CH, Mintzer J, et al. (2015) A randomized, double-blind, placebo-controlled trial of resveratrol for Alzheimer disease. Neurology 85: 1383-1391.

111. Van Gool WA, Weinstein HC, Scheltens P, Walstra GJ (2001) Effect of hydroxychloroquine on progression of dementia in early Alzheimer's disease: an 18-month randomised, double-blind, placebo-controlled study. Lancet 358: 455-460.

112. Vellas B, Sol O, Snyder PJ, Ousset PJ, Haddad R, et al. (2011) EHT0202 in Alzheimer's disease: A 3-Month, randomized, placebo-controlled, Double-Blind study. Curr Alzheimer Res 8: 203-212.

113. Wang T, Huang Q, Reiman EM, Chen K, Li X, et al. (2013) Effects of memantine on clinical ratings, fluorodeoxyglucose positron emission tomography measurements, and cerebrospinal fluid assays in patients with moderate to severe Alzheimer dementia: a 24-week, randomized, clinical trial. J Clin Psychopharmacol 33: 636-642.

114. Watson GS, Cholerton BA, Reger MA, Baker LD, Plymate SR, et al. (2005) Preserved cognition in patients with early Alzheimer disease and amnestic mild cognitive impairment during treatment with rosiglitazone: a preliminary study. Am J Geriatr Psychiatry 13: 950-958.

115. Wilcock GK, Black SE, Hendrix SB, Zavitz KH, Swabb EA, et al. (2008) Efficacy and safety of tarenflurbil in mild to moderate Alzheimer's disease: a randomised phase II trial. Lancet Neurol 7: 483-493.

116. Winblad B, Bonura ML, Rossini BM, Battaglia A (2001) Nicergoline in the treatment of mild-to-moderate Alzheimer's disease: A European multicentre trial. Clin Drug Investig 21: 621-632.

117. Winblad B, Andreasen N, Minthon L, Floesser A, Imbert G, et al. (2012) Safety, tolerability, and antibody response of active Abeta immunotherapy with CAD106 in patients with Alzheimer's disease: randomised, double-blind, placebo-controlled, first-in-human study. Lancet Neurol 11: 597-604.

118. Wischik CM (2015) Tau Aggregation Inhibitor Therapy: An Exploratory Phase 2 Study in Mild or Moderate Alzheimer's Disease. J Alzheimers Dis 44: 705-720 716p.

119. Wolkowitz OM, Kramer JH, Reus VI, Costa MM, Yaffe K, et al. (2003) DHEA treatment of Alzheimer's disease: a randomized, double-blind, placebo-controlled study. Neurology 60: 1071-1076.

120. Annweiler C, Fantino B, Parot-Schinkel E, Thiery S, Gautier J, et al. (2011) Alzheimer's disease--input of vitamin D with mEmantine assay (AD-IDEA trial): study protocol for a randomized controlled trial. Trials 12: 230.

121. Egefjord L, Gejl M, Møller A, Brændgaard H, Gottrup H, et al. (2012) Effects of liraglutide on neurodegeneration, blood flow and cognition in Alzheimer´s disease - protocol for a controlled, randomized double-blinded trial. Dan Med J 59: A4519.

122. Lawlor B, Kennelly S, O'Dwyer S, Cregg F, Walsh C, et al. (2014) NILVAD protocol: a European multicentre double-blind placebo-controlled trial of nilvadipine in mild-to-moderate Alzheimer's disease. BMJ Open 4: e006364.

123. MRC/NIHR Efficacy and Mechanism Evaluation Programme ISRCTN16105064 Minocycline in Alzheimers disease.

124. Alzheimer's Society (UK) BHFU ISRCTN31208535 A clinical trial to test amlodipine as a new treatment for vascular dementia.

125. Alzheimer's Society AsDDFU, Van Geest Foundation and King's BRC (UK), Novo Nordisk, ISRCTN89711766 Evaluating the effects of the novel GLP1 analogue, Liraglutide, in patients with Alzheimer's Disease (ELAD study).

126. National Institute for Health Research ISRCTN93682878 Reducing pathology in Alzheimer's disease through angiotensin targeting.

127. University of Colorado Denver TDF NCT01409915 Study of the Safety & Efficacy of Leukine® in the Treatment of Alzheimer's Disease.

128. Instituto G, S.A., Grifols Biologicals Inc. NCT01561053 A Study to Evaluate Albumin and Immunoglobulin in Alzheimer's Disease (AMBAR).

129. Eisai Inc NCT01767311 A Study to Evaluate Safety, Tolerability, and Efficacy of BAN2401 in Subjects With Early Alzheimer's Disease.

130. University of Pennsylvania NCT01965756 Effect of Insulin Sensitizer Metformin on AD Biomarkers.

131. University oC, San Francisco NCT01966666 A Safety, Tolerability, Pharmacokinetics, Pharmacodynamics and Preliminary Efficacy Study of TPI-287 in Alzheimer's Disease.

132. AstraZeneca NCT02036645 SAD/MAD Study to Assess Safety, Tolerability, PK & PD of MEDI1814 in Subjects With Mild-Moderate Alzheimer's Disease.

133. Hoffmann-La Roche NCT02051608 A Study of Gantenerumab in Patients With Mild Alzheimer Disease.

134. Therapeutics v NCT02080364 Evaluation of the Efficacy and Safety of Azeliragon (TTP488) in Patients With Mild Alzheimer's Disease (STEADFAST).

135. Eli Lilly and Company A NCT02245737 An Efficacy and Safety Study of LY3314814 in Early Alzheimer's Disease (AMARANTH).

136. Eisai Inc. B NCT02322021 Dose-Finding Study To Evaluate Safety, Tolerability, and Efficacy of E2609 in Subjects With Mild Cognitive Impairment Due to Alzheimer's Disease (Prodromal Alzheimer's Disease) and Mild to Moderate Dementia Due to Alzheimer's Disease.

137. Genentech Inc NCT02353598 A Study of Crenezumab in Participants With Mild to Moderate Alzheimer Disease.

138. GliaCure Inc NCT02386306 Study Evaluating Safety, Tolerability, and PK of Multiple Ascending Doses of GC021109 in Subjects With Mild to Moderate Alzheimer's Disease.

139. Probiodrug A, Julius Clinical, VU University Medical Center NCT02389413 Safety and Tolerability of PQ912 in Subjects With Early Alzheimer's Disease (SAPHIR).

140. Janssen Research & Development L NCT02406027 An Extension Study to Evaluate the Long-Term Safety and Tolerability of JNJ-54861911 in Participants in the Early Alzheimer's Disease Spectrum.

141. Inc. NB NCT02431468 A Study Assessing Bryostatin in the Treatment of Moderately Severe to Severe Alzheimer's Disease.

142. Biogen NCT02434718 Single and Multiple Ascending Dose Study of BIIB037 in Japanese Participants With Alzheimer's Disease (PROPEL)

143. Biogen NCT02477800 221AD301 Phase 3 Study of Aducanumab (BIIB037) in Early Alzheimer's Disease (ENGAGE).

144. Biogen NCT02484547 221AD302 Phase 3 Study of Aducanumab (BIIB037) in Early Alzheimer's Disease (EMERGE).

145. HealthPartners Institute NCT02503501 Intranasal Glulisine in Amnestic Mild Cognitive Impairment and Probable Mild Alzheimer's Disease.

146. AZTherapies Inc PG, KCAS Bio, APCER Life Sciences, NCT02547818 Safety and Efficacy Study of ALZT-OP1 in Subjects With Evidence of Early Alzheimer's Disease.

147. United Neuroscience Ltd NCT02551809 Evaluate the Safety, Tolerability, Immunogenicity and Efficacy of UB-311 in Mild Alzheimer's Disease (AD) Patients.

148. Axon Neuroscience SE NCT02579252 24 Months Safety and Efficacy Study of AADvac1 in Patients With Mild Alzheimer's Disease (ADAMANT).

149. Longeveron LLC NCT02600130 A Allogeneic Human Mesenchymal Stem Cell Infusion Versus Placebo in Patients With Alzheimer's Disease.

150. Rosen W, Mohs R, Davis K. (1984) A new rating scale for Alzheimer's disease. Am J Psychiatry 141: 1356-1364.

151. Sansoni J, Marosszeky N, Jeon Y, Chenoweth L, Hawthorne G, King MT, et al (2007) Dementia Outcomes Measurement Suite (DOMS) Project: Final Report. Centre for Health Service Development, University of

Wollongong. .

152. Peña-Casanova J, Aguilar M, Santacruz P, Bertran-Serra I, Hernández G, Sol JM, et al (1997) [Adaptation and normalization of the Alzheimer's disease Assessment Scale for Spain (NORMACODEM)]. Neurologia 12: 69-77.

153. Rockwood K, Fay, S., & Gorman, M. (2010) The ADAS‐cog and clinically meaningful change in the VISTA clinical trial of galantamine for Alzheimer's disease. Int J Geriatr Psychiatry 25: 191-201.

154. Schrag A, Schott, J. M., & Alzheimer's Disease Neuroimaging Initiative. (2011) What is the clinically relevant change on the ADAS-Cog? J Neurol Neurosurg Psychiatry 83: 171-173.

155. Karin A, Hannesdottir, K., Jaeger, J., Annas, P., Segerdahl, M., Karlsson, P., et al (2014) Psychometric evaluation of ADAS‐Cog and NTB for measuring drug response. Acta Neurol Scand 129: 114-122.

156. Sevigny JJ, Peng, Y., Liu, L., & Lines, C. R (2010) Item analysis of ADAS-Cog: Effect of baseline cognitive impairment in a clinical AD trial. Am J Alzheimers Dis Other Demen 25: 119–124.

157. Grochowalski JH, Liu, Y., & Siedlecki, K. L. (2015) Examining the reliability of ADAS-Cog change scores. Aging Neuropsychol Cogn: 1-17.

158. Mohs R, Cohen L. (1988) Alzheimer’s Disease Assessment Scale (ADAS). Psychopharmacol Bull 24: 627-628.

159. Chu L, Chiu KC, Hui SL, Yu GK, Tsui WJ, Lee PW. (2000) The reliability and validity of the Alzheimer's Disease Assessment Scale Cognitive Subscale (ADAS-Cog) among the elderly Chinese in Hong Kong. Ann Acad Med Singapore 29: 474-485.

160. Liu H, Lee Teng E, Chuang YY, Lin KN, Fuh JL, Wang PN (2002) The Alzheimer's Disease Assessment Scale: findings from a loweducation population. Dement Geriatr Cogn Disord 13: 21-26.

161. Weyer G, Erzigkeit H, Kanowski S, Ihl R, Hadler D. (1997) Alzheimer’s Disease Assessment Scale: reliability and validity in a multicenter clinical trial. Int Psychogeriatr 9: 123-138.

162. Kim Y, Nibbelink DW, Overall JE (1994) Factor structure and reliability of the Alzheimer's Disease Assessment Scale in a multicenter trial with linopirdine. J Geriatr Psychiatry Neurol 7: 74-83.

163. Folstein M, Folstein SE, McHugh PR (1975) Mini-Mental State: a practical method for grading the cognitive state of patients for the clinician. J Psychiatr Res 12: 189-198.

164. Fountoulakis K, Tsolaki M, Chantzi H, Kazis A (2000) Mini mental state examination (MMSE): a validation study in Greece. Am J Alzheimers Dis Other Demen 15: 342–345.

165. Beaman S, Beaman PE, Garcia-Peña C, Villa MA, Heres J, Córdova A, et al (2004) Validation of a modified version of the Mini-Mental State Examination (MMSE) in Spanish. Aging Neuropsychol Cogn 11: 1–11.

166. Ansari NN, Naghdi, S., Hasson, S., Valizadeh, L., & Jalaie, S (2010) Validation of a Mini-Mental State Examination (MMSE) for the Persian population: a pilot study. Appl Neuropsychol 17: 190-195.

167. Awan S, Shahbaz, N., Akhtar, S. W., Ahmad, A., Iqbal, S., Ahmed, S., et al (2015) Validation study of the Mini-Mental State Examination in Urdu language for Pakistani population. Open Neurol J 9: 53.

168. Carnero-Pardo C (2014) Should the mini-mental state examination be retired? Neurología 29: 473-481.

169. Rakuša M, Granda, G., Kogoj, A., Mlakar, J., & Vodušek, D. B. (2006) Mini‐Mental State Examination: standardization and validation for the elderly Slovenian population. Eur J Neurol 13: 141-145.

170. Howard R, Phillips, P., Johnson, T., O'Brien, J., Sheehan, B., Lindesay, J., et al (2011) Determining the minimum clinically important differences for outcomes in the DOMINO trial. Int J Geriatr Psychiatry 26: 812-817.

171. Galasko DR, Gould, R. L., Abramson, I. S., Salmon, D. P. (2000) Measuring cognitive change in a cohort of patients with Alzheimer's disease. Stat Med 19: 1421-1432.

172. Salmon D, Thal LJ, Butters N, Heindel WC. (1990) Longitudinal evaluation of dementia of the Alzheimer type A comparison of 3 standardized mental status examinations. Neurology 40: 1225.

173. Velayudhan L, Ryu SH, Raczek M, Philpot M, Lindesay J, Critchfield M, et al. (2014) Review of brief cognitive tests for patients with suspected dementia. Int Psychogeriatr 26: 1247-1262.

174. Schramm U, Berger, G., Müller, R., Kratzsch, T., Peters, J., & Frölich, L. (2002) Psychometric properties of Clock Drawing Test and MMSE or Short Performance Test (SKT) in dementia screening in a memory clinic population. Int J Geriatr Psychiatry 17: 254-260.

175. Shen J, Shen Q, Yu H, Lai JS, Beaumont JL, Zhang Z, et al (2014) Validation of an Alzheimer’s disease assessment battery in Asian participants with mild to moderate Alzheimer’s disease. Am J Neurodegener Dis 3: 158.

176. Tombaugh TN (2005) Test-retest reliable coefficients and 5-year change scores for the MMSE and 3MS. Arch Clin Neuropsychol 20: 485-503.

177. Pangman VC, Sloan, J., & Guse, L. (2000) An examination of psychometric properties of the mini-mental state examination and the standardized mini-mental state examination: implications for clinical practice. Appl Nurs Res 13: 209-213.

178. Rossetti H, Cullum CM, Hynan LS, Lacritz L. (2010) The CERAD Neuropsychological Battery Total Score and the Progression of Alzheimer's Disease. Alzheimer Dis Assoc Disord 24: 138–142.

179. Demers P, Robillard, A., Laflèche, G., Nash, F., Heyman, A., & Fillenbaum, G. (1994) Translation of clinical and neuropsychological instruments into French: the CERAD experience. Age Ageing 23: 449-451.

180. Glezerman A, & Drexler, M. L. (2001) The Russian Adaptation of the CERAD Battery (CERAD-RA). Arch Clin Neuropsychol 16: 826-826.

181. Liu KP, Kuo, M. C., Tang, K. C., Chau, A. W., Ho, I. H., Kwok, M. P., et al (2011) Effects of age, education and gender in the Consortium to Establish a Registry for the Alzheimer's Disease (CERAD)-Neuropsychological Assessment Battery for Cantonese-speaking Chinese elders. Int Psychogeriatr 23: 1575-1581.

182. Lee JH, Lee, K. U., Lee, D. Y., Kim, K. W., Jhoo, J. H., Kim, J. H., et al (2002) Development of the Korean Version of the Consortium to Establish a Registry for Alzheimer's Disease Assessment Packet (CERAD-K) Clinical and Neuropsychological Assessment Batteries. J Gerontol B Psychol Sci Soc Sci 57: 47-53.

183. Haanpää RM, Suhonen, N. M., Hartikainen, P., Koivisto, A. M., Moilanen, V., Herukka, S. K.,et al (2015) The CERAD Neuropsychological Battery in Patients with Frontotemporal Lobar Degeneration. Dement Geriatr Cogn Disord Extra 5: 147-154.

184. Seo EH, Lee, D. Y., Lee, J. H., Choo, I. H., Kim, J. W., Kim, S. G., et al (2010) Total scores of the CERAD neuropsychological assessment battery: validation for mild cognitive impairment and dementia patients with diverse etiologies. Am J Geriatr Psychiatry 18: 801-809.

185. Paajanen T, Hänninen, T., Aitken, A., Hallikainen, M., Westman, E., Wahlund, L. O., et al (2013) CERAD neuropsychological total scores reflect cortical thinning in prodromal Alzheimer's disease. Dement Geriatr Cogn Disord Extra 3: 446-458.

186. Hallikainen I, Hänninen T, Fraunberg M, Hongisto K, Välimäki T, Hiltunen A, et al (2013) Progression of Alzheimer's disease during a three-year follow-up using the CERAD-NB total score: Kuopio ALSOVA study. Int Psychogeriatr 25: 1335-1344.

187. Trapp-Moen B, Tyrey, M., Cook, G., Heyman, A., & Fillenbaum, G. G. (2001) In-Home Assessment of Dementia by Nurses Experience Using the CERAD Evaluations. Gerontologist 41: 406-409.

188. Maruff P, Thomas E, Cysique L, et al. (2009) Validity of the CogState brief battery: relationship to standardized tests and sensitivity to cognitive impairment in mild traumatic brain injury, schizophrenia, and AIDS dementia complex. Arch Clin Neuropsychol 24: 165-178.

189. Hammers D, Spurgeon E, Ryan K, Persad C, Barbas N, Heidebrink J, et al (2012) Validity of a brief computerized cognitive screening test in dementia. J Geriatr Psychiatry Neurol 25: 89-99.

190. Hammers D, Spurgeon, E., Ryan, K., Persad, C., Heidebrink, J., Barbas, N., et al (2011) Reliability of repeated cognitive assessment of dementia using a brief computerized battery. Am J Alzheimers Dis Other Demen 26: 326-333.

191. Fredrickson J, Maruff, P., Woodward, M., Moore, L., Fredrickson, A., Sach, J., et al (2010) Evaluation of the usability of a brief computerized cognitive screening test in older people for epidemiological studies. Neuroepidemiology 34: 65–75.

192. Lim YY, Ellis, K. A., Harrington, K., Ames, D., Martins, R. N., Masters, C. L., et al (2012) Use of the CogState Brief Battery in the assessment of Alzheimer's disease related cognitive impairment in the Australian Imaging Biomarkers and Lifestyle (AIBL) study. J Clin Exp Neuropsychol 34: 345-358.

193. Maruff P, Lim, Y. Y., Darby, D., Ellis, K. A., Pietrzak, R. H., Snyder, P. J., et al (2013) Clinical utility of the cogstate brief battery in identifying cognitive impairment in mild cognitive impairment and Alzheimer’s disease. BMC Psychol 1: 30.

194. Harrison J, Minassian, S. L., Jenkins, L., Black, R. S., Koller, M., & Grundman, M. (2007) A neuropsychological test battery for use in Alzheimer disease clinical trials. Arch Neurol 64: 1323-1329.

195. Harrison J, Rentz, D. M., McLaughlin, T., Niecko, T., Gregg, K. M., Black, R. S., et al (2014) Cognition in MCI and Alzheimer’s Disease: Baseline Data from a Longitudinal Study of the NTB. Clin Neuropsychol 28: 252-268.

196. Fernández M, Gobartt AL, Balañá M (2010) Behavioural symptoms in patients with Alzheimer's disease and their association with cognitive impairment. BMC Neurol 10: 1.

197. Cano SJ, Posner, H. B., Moline, M. L., Hurt, S. W., Swartz, J., Hsu, T., et al (2010) The ADAS-cog in Alzheimer's disease clinical trials: psychometric evaluation of the sum and its parts. J Neurol Neurosurg Psychiatry 81: 1363-1368.

198. Yesavage J, Poulsen SL, Sheikh J, Tanke E (1988) Rates of change of common measures of impairment in senile dementia of the Alzheimer’s type. Psychopharmacol Bull 24: 531-534.

199. Standish T, Molloy DW, Bédard M, Layne EC, Murray E, Strang D (1996) Improved reliability of the Standardized Alzheimer’s Disease Assessment Scale (SADAS) compared with the Alzheimer’s Disease Assessment Scale (ADAS). J Am Geriatr Soc 44: 712-716.

200. Reisberg B, Borenstein J, Salob SP, Ferris SH, Franssen E, Georgotas A (1987) Behavioral symptoms in Alzheimer's disease: phenomenology and treatment. J Clin Psychiatry 48: 9-15.

201. Monteiro I, Boksay I, Auer SR, Torossian C, Ferris SH, Reisberg B. (2001) Addition of a frequency-weighted score to the Behavioral Pathology in Alzheimer's Disease Rating Scale: the BEHAVE-AD-FW: methodology and reliability. Eur Psychiatry 16: 5-24.

202. Sclan S, Saillon A, Franssen E, Hugonot‐Diener La, Saillon A, Reisberg B (1996) The Behavior Pathology in Alzheimer's Disease Rating Scale (BEHAVEAD): Reliability and analysis of symptom category scores. Int J Geriatr Psychiatry 11: 819-830.

203. Katz I, Jeste DV, Mintzer JE, Clyde C, Napolitano J, Brecher M. (1999) Comparison of risperidone and placebo for psychosis and behavioral disturbances associated with dementia: a randomized, double-blind trial. Risperidone Study Group. J Clin Psychiatry 60: 107–115.

204. Auer S, Monteiro IM, Reisberg B (1996) The empirical behavioral pathology in Alzheimer's disease (E-BEHAVE-AD) rating scale. Int Psychogeriatr 8: 247-266.

205. Overall J, Gorham D. (1962) The Brief Psychiatric Scale. Psychol Rep 10: 799-812.

206. Gottlieb G, Gur RE, Gur RC (1997) Reliability of psychiatric scales in patients with Dementia of the Alzheimer Type. Am J Psychiatry 145: 857-860.

207. Tariot P, Mack JL, Patterson MB, Edland SD, Weiner MF, Fillenbaum G et al. (1995) The Behavior Rating Scale for Dementia of the Consortium to Establish a Registry for Alzheimer's Disease. The Behavioral Pathology Committee of the Consortium to Establish a Registry for Alzheimer's Disease. Am J Psychiatry 152: 1349-1357.

208. Fillenbaum G, van Belle G, Morris JC, Mohs RC, Mirra SS, Davis PC, et al (2008) Consortium to Establish a Registry for Alzheimer’s Disease (CERAD): the first twenty years. Alzheimers Dement 4: 96-109.

209. Patterson M, Mack JL, Mackell JA, Thomas R, Tariot P, Weiner M, et al (1997) A longitudinal study of behavioral pathology across five levels of dementia severity in Alzheimer’s disease: the CERAD Behavior Rating Scale for Dementia. Alzheimer Dis Assoc Disord 11: 40-44.

210. Molloy D, Mcllroy WE, Guyatt GH, Lever JA (1991) Validity and reliability of the Dysfunctional Behaviour Rating Instrument. Acta Psychiatr Scand 84: 103-106.

211. Molloy D, Bédard M, Guyatt GH, Lever J. (1997) Dysfunctional behavior rating instrument. Int Psychogeriatr 8: 333-341.

212. Cummings J, Mega M, Gray K, Rosenberg-Thompson S, Carusi DA, Gornbein J. (1994) The Neuropsychiatric Inventory: comprehensive assessment of psychopathology in dementia. Neurology 44: 2308-2314.

213. Frisoni G, Rozzini L, Gozzetti A, Binetti G, Zanetti O, Bianchetti A et al (1999) Behavioural syndromes in Alzheimer’s Disease: description and correlates. Dement Geriatr Cogn Disord 10: 130-138.

214. Mega M, Cummings JL, Fiorello R, Gornbein J (1996) The spectrum of behavioural change in Alzheimer’s Disease. Neurology 46: 130-135. .

215. Hatoum H, Lin S-J, Arcona S, Thomas SK, Koumaris B, Mirski D (2005) The use of the occupational disruptiveness scale of the neuropsychiatric inventory-nursing home version to measure the impact of rivistagmine on the disruptive behaviour of nursing home residents with Alzheimer’s disease. J Am Med Dir Assoc 6: 238-245.

216. Kaufer D, Cummings JL, Christine D (1998) Differential neuropsychiatric symptom response in Tacrine in Alzhiemer’s disease: relationship to symptom severity. J Neuropsychiatry Clin Neurosci 10: 5563.

217. Cummings J, Street J, Masterman D, Clarke WS (2002) Efficacy of olanzipine in the treatment of psychosis in dementia with lewy bodies. Dement Geriatr Cogn Disord 13: 67-73.

218. Cummings J (1997) The Neuropsychiatric Inventory: assessing psychopathology in dementia patients. Neurology 48: 10-16.

219. Spiegel R, Brunner C, Erminifunfschilling D, Monsch A, Notter M, Puxty J et al (1991) A New Behavioral-Assessment Scale for Geriatric Out-Patients and Inpatients - the Nosger (Nurses Observation Scale for Geriatric-Patients). J Am Geriatr Soc 39: 339-347.

220. Wahle M, Häller S, Spiegel R. (1996) Validation of the NOSGER (Nurses' Observation Scale for Geriatric Patients): reliability and validity of a caregiver rating instrument. Int Psychogeriatr 8: 525-547.

221. Tremmel L, Spiegel R. (1993) Clinical experience with the NOSGER (Nurses' Observation Scale for Geriatric Patients): Tentative normative data and sensitivity to change. Int J Geriatr Psychiatry 8: 311-317.

222. Plutchik R, Conte H, Lieberman M, Bakur M, Grossman J, Lehrman N. (1970) Reliability and validity of a scale for assessing the functioning of geriatric patients. J Am Geriatr Soc 18: 491-500.

223. Teri L, Truax P, Logsdon R, Uomoto J, Zarit S, Vitaliano PP (1992) Assessment of behavioral problems in dementia: the revised memory and behavior problems checklist. Psychol Aging 7: 622-631.

224. Fuh JL, Liu, C. Y., Wang, S. J., Wang, H. C., & Liu, H. C. (1999) Revised memory and behavior problems checklist in Taiwanese patients with Alzheimer's disease. Int Psychogeriatr 11: 181-189.

225. Nogales-González C, Losada, A., & Romero-Moreno, R. (2015) Confirmatory factor analysis of the Spanish version of the revised memory and behavior problems checklist. Int Psychogeriatr 27: 683-692.

226. Johnson MM, Wackerbarth, S. B., Schmitt, F. A. (2001) Revised memory and behavior problems checklist. Clin Gerontol 22: 87-108.

227. Weiner MF, Tractenberg, R., Teri, L., Logsdon, R., Thomas, R. G., Gamst, A., et al (2000) Quantifying behavioral disturbance in Alzheimer's disease patients. J Psychiatr Res 34: 163-167.

228. Smith S, Lamping DL, Banerjee S, Harwood R, Foley B, Smith P (2005) Measurement of health-related quality of life for people with dementia: development of a new instrument (DEMQOL) and an evaluation of current methodology. Health Technol Assess 9.

229. Lucas-Carrasco R, Lamping, D. L., Banerjee, S., Rejas, J., Smith, S. C., & Gómez-Benito, J. (2010) Validation of the Spanish version of the DEMQOL system. Int Psychogeriatr 22: 589-597.

230. Berwig M, Leicht, H., Hartwig, K., & Gertz, H. J. (2011) Self-rated quality of life in mild cognitive impairment and Alzheimer’s disease: The problem of affective distortion. GeroPsych 24: 45-51.

231. Gavrilova SI, Ferri, C. P., Mikhaylova, N., Sokolova, O., Banerjee, S., & Prince, M. (2009) Helping carers to care—The 10/66 dementia research group’s randomized control trial of a caregiver intervention in Russia. Int J Geriatr Psychiatry 24: 347–354.

232. Shi G, Liu, C., Li, Q., Zhu, H., & Wang, L. (2012) Influence of acupuncture on cognitive function and markers of oxidative DNA damage in patients with vascular dementia. J Tradit Chin Med 32: 199–202.

233. Orrell M, Aguirre E, Spector A, Hoare Z, Woods RT, Streater A, et al (2014) Maintenance cognitive stimulation therapy for dementia: single-blind, multicentre, pragmatic randomised controlled trial. Br J Psychiatry 204: 454-461.

234. Low L-F, Brodaty, H., Goodenough, B., Spitzer, P., Bell, J.-P., Fleming, R., et al (2013) The Sydney Multisite Intervention of LaughterBosses and ElderClowns (SMILE) study: cluster randomised trial of humour therapy in nursing homes. BMJ Open 3: e002072.

235. Schulz R, Cook, T. B., Beach, S. R., Lingler, J. H., Martire, L. M., Monin, J. K., et al (2013) Magnitude and Causes of Bias Among Family Caregivers Rating Alzheimer Disease Patients. Am J Geriatr Psychiatry 21: 14-25.

236. Schulz R, Monin, J. K., Czaja, S. J., Lingler, J. H., Beach, S. R., Martire, L. M., et al (2010) Measuring the Experience and Perception of Suffering. Gerontologist 50: 774–784.

237. EuroQol Group (1990) EuroQol-a new facility for the measurement of health-related quality of life. Health Policy 16: 199-208.

238. Aguirre E, Kang, S., Hoare, Z., Edwards, R. T., & Orrell, M. (2016) How does the EQ-5D perform when measuring quality of life in dementia against two other dementia-specific outcome measures? Qual Life Res 25: 45-49.

239. Hounsome N, Orrell, M., & Edwards, R. T. (2011) EQ-5D as a quality of life measure in people with dementia and their carers: evidence and key issues. Value Health 14: 390-399.

240. Ankri J, Beaufils, B., Novella, J. L., Morrone, I., Guillemin, F., Jolly, D., et al. (2003) Use of the EQ-5D among patients suffering from dementia. Clin Epidemiol 56: 1055-1063.

241. Naglie G, Tomlinson G, Tansey C, Irvine J, Ritvo P, Black SE, et al (2006) Utility-based quality of life measures in Alzheimer's disease. Qual Life Res 15: 631-643.

242. Coucill W, Bryan S, Bentham P, et al (2001) ED-5D in patients with dementia. Med Care 8: 760–771.

243. Logsdon R, Gibbons LE, McCurry SM, Teri L. (1999) Quality of life in Alzheimer’s disease: patient and caregiver reports. J Ment Health Aging 5: 21-32.

244. Thorgrimsen L, Selwood A, Spector A, Royan L, de Madariaga Lopez M, Woods RT, Orrell M. (2003) Whose quality of life is it anyway? The validity and reliability of the Quality of Life-Alzheimer's Disease (QoL-AD) scale. Alzheimer Dis Assoc Disord 17: 201-208.

245. Bowling A, Rowe, G., Adams, S., Sands, P., Samsi, K., Crane, M., et al (2015) Quality of life in dementia: a systematically conducted narrative review of dementia-specific measurement scales. Aging Ment Health 19: 13-31.

246. Naglie G, Hogan DB, Krahn M, Black SE, Beattie BL, Patterson C, et al (2011) Predictors of family caregiver ratings of patient quality of life in Alzheimer disease: cross-sectional results from the Canadian Alzheimer's Disease Quality of Life Study. Am J Geriatr Psychiatry 19: 891-901.

247. Wolak A, Novella, J. L., Drame, M., Guillemin, F., Di Pollina, L., Ankri, J., et al (2009) Transcultural adaptation and psychometric validation of a French-language version of the QoL-AD. Aging Ment Health 13: 593-600.

248. Woods B, Thorgrimsen L, Spector A, Royan L, Orrell M (2006) Improved quality of life and cognitive stimulation therapy in dementia. Aging Ment Health 10: 219 –226.

249. Selwood A, Thorgrimsen L, Orrell M (2005) Quality of life in dementia-a one-year follow-up study. Int J Geriatr Psychiatry 20: 232-237.

250. Teri L, McCurry SM, Logsdon R, Gibbons LE (2005) Training community consultants to help family members improve dementia care: a randomized controlled trial. Gerontologist 45: 802-811.

251. Sloane P, Zimmerman S, Williams CS, Reed PS, Gill KS, Preisser JS. (2005) Evaluating the quality of life of long-term care residents with dementia. Gerontologist 45: 37-49.

252. Chapman S, Weiner MF, Rackley A, Hynan LS, Zientz J (2004) Effects of cognitive-communication stimulation for Alzheimer's disease patients treated with donepezil. J Speech Lang Hear Res 47: 1149-1163.

253. Orrell M, Spector A, Thorgrimsen L, Woods B (2005) A pilot study examining the effectiveness of maintenance Cognitive Stimulation Therapy (MCST) for people with dementia. Int J Geriatr Psychiatry 20: 446-451.

254. Spector A, Thorgrimsen L, Woods BO, Royan L, Davies S, Butterwoth M, et al (2003) Br J Psychiatry 183: 248-254.

255. Edelman P, Fulton BR and Kuhn D (2004) Comparison of dementia-specific quality of life measures in adult day centers. Home Health Care Serv Q 23: 25-42.

256. Edelman P, Fulton BR, Kuhn D, Chang CH. (2005) A comparison of three methods of measuring dementia-specific quality of life: perspectives of residents, staff, and observers. Gerontologist 45: 27-36.

257. Logsdon R, Gibbons LE, McCurry SM, Teri L. (2002) Assessing quality of life in older adults with cognitive impairment. Psychosom Med 64: 510-519.

258. Fuh J, Wang SJ (2005) Assessing quality of life in Taiwanese patients with Alzheimer's disease. Int J Geriatr Psychiatry 21: 103-107.

259. Spector A, Orrell M (2006) Quality of Life (QoL) in Dementia: A Comparison of the Perceptions of People With Dementia and Care Staff in Residential Homes. Alzheimer Dis Assoc Disord 20: 160-165.

260. Hoe J, Hancock G, Livingston G, Orrell M (2006) Quality of life of people with dementia in residential care homes. Br J Psychiatry 188: 460-464.

261. Shin I, Carter M, Masterman D, Fairbanks L, Cummings JL (2005) Neuropsychiatric symptoms and quality of life in Alzheimer disease. Am J Geriatr Psychiatry 13: 469-474.

262. Galasko D, Bennet D, Sano M, Ernesto C, Thomas R, Grundman M, Ferris S (1997) An inventory to assess activities of daily living for clinical trials in Alzheimer’s disease. The Alzheimer’s Disease Cooperative Study. Alzheimer Dis Assoc Disord 11: 33-39.

263. Galasko D, Bennett, D. A., Sano, M., Marson, D., Kaye, J., & Edland, S. D. (2006) ADCS Prevention Instrument Project: assessment of instrumental activities of daily living for community-dwelling elderly individuals in dementia prevention clinical trials. Alzheimer Dis Assoc Disord 20: 152-169.

264. Bullock R, Bergman H, Touchon J (2006) Effect of age on response to rivastigmine or donepezil in patients with Alzheimer's disease. Curr Med Res Opin 22: 483-494.

265. Galasko D, Kershaw PR, Schneider L, et al (2004) Galantamine maintains ability to perform activities of daily living in patients with Alzheimer's disease. J Am Geriatr Soc 52: 1070-1076.

266. Brodaty H, Corey-Bloom J, Potocnik FCV, et al (2005) Galantamine prolonged-release formulation in the treatment of mild to moderate Alzheimer's disease. Dement Geriatr Cogn Disord 20: 120-132.

267. Peskind E, Potkin SG, Pomara N, et al (2006) Memantine treatment in mild to moderate Alzheimer disease: a 24-week randomized, controlled trial. Am J Geriatr Psychiatry 14: 704-715.

268. Schneider L (2001) Assessing outcomes in Alzheimer Disease. Alzheimer Dis Assoc Disord 15: 8-18.

269. Burns A, Lawlor B, Craig S (2004) Assessment scales in old age psychiatry (2nd ed.). London: Taylor & Francis.

270. Manero RM, Casals-Coll, M., Sánchez-Benavides, G., Rodríguez-de Los Reyes, O. N., Aguilar, M., Badenes, D., et al (2014) Diagnostic Validity of the Alzheimer's Disease Functional Assessment and Change Scale in Mild Cognitive Impairment and Mild to Moderate Alzheimer's Disease. Dement Geriatr Cogn Disord 37: 366-375.

271. Bucks RS AD, Wilcock GK, Siegfried K (1996) Assessment of activities of daily living in dementia: Development of the Bristol activities of daily living scale. Age Ageing 25: 113-120.

272. Byrne LM, Wilson, P., Bucks, R. S., Hughes, A. O., & Wilcock, G. K. (2000) The sensitivity to change over time of the Bristol Activities of Daily Living Scale in Alzheimer's disease. Int J Geriatr Psychiatry 15: 656-661.

273. Bucks RS, & Haworth, J. (2002) Bristol activities of daily living scale: a critical evaluation. Expert Rev Neurother 2: 669-676.

274. Stern Y, Albert SM, Sano M, et al (1994) Assessing patient dependence in Alzheimer’s disease. J Gerontol 49: 216-222.

275. Demers L, Oremus M, Perrault A, Champoux N, Wolfson C. (2000) Review of outcome measurement instruments in Alzheimer's disease drug trials: psychometric properties of functional and quality of life scales. J Geriatr Psychiatry Neurol 13: 170-180.

276. Bavazzano A, Magnolfi SU, Calvani D, Valente C, Boni F, Baldini A, et al (1998) Functional evaluation of Alzheimer patients during clinical trials: a review. Arch Gerontol Geriatr 26: 27–32.

277. Gelinas I, Gauthier L, McIntyre M, Gauthier S. (1999) Development of a functional measure for persons with Alzheimer's disease: the disability assessment for dementia. Am J Occup Ther 53: 471-481.

278. Suh GH (2003) Development of the Korean Version of Disability Assessment for Dementia Scale (DAD-K) to assess function in dementia. J Korean Geriatr Soc 7: 278-287.

279. Mok CC, Siu, A. M., Chan, W. C., Yeung, K. M., Pan, P. C., & Li, S. W. (2005) Functional disabilities profile of Chinese elderly people with Alzheimer’s disease–a validation study on the Chinese version of the Disability Assessment for Dementia. 20: 112-119.

280. De Vreese LP, Caffarra, P., Savarè, R., Cerutti, R., Franceschi, M., Grossi, E. (2008) Functional disability in early Alzheimer’s disease – A validation study of the Italian version of the disability assessment for dementia scale. Dement Geriatr Cogn Disord 25: 186-194.

281. Carthery-Goulart MT, Areza-Fegyveres, R., Schultz, R. R., Okamoto, I., Caramelli, P., Bertolucci, P. H. F., et al (2007) Cross-cultural adaptation of the Disability Assessment for Dementia (DAD). Arq Neuropsiquiatr 65: 916-919.

282. Sánchez-Pérez A, López-Roig, S., Pérez, A. P., Gómez, P. P., Pastor, M. Á., & Pomares, M. H. (2015) Validation Study of the Spanish Version of the Disability Assessment for Dementia Scale. . Medicine 94: e1925.

283. Tozlu M, Cankurtaran, M., Yavuz, B. B., Cankurtaran, E. S., Kutluer, İ., Erkek, B. M. (2014) Functional Disability in Alzheimer Disease A Validation Study of the Turkish Version of the Disability Assessment for Dementia Scale. J Geriatr Psychiatry Neurol 27: 237-246

284. Zangbar HS, Mehraban, A. H., Akbarfahimi, M., & Rasanani, F. M. (2016) Validity and Reliability of the Persian Version of the Disability Assessment for Dementia Scale. Middle East Journal of Rehabilitation and Health 3: e35619.

285. Feldman H, Sauter A, Donald A, et al (2001) The disability assessment for dementia scale: a 12-month study of functional ability in mild to moderate severity Alzheimer disease. Alzheimer Dis Assoc Disord 15: 89-95.

286. Arrighi HM, Gélinas, I., McLaughlin, T. P., Buchanan, J., Gauthier, S. (2013) Longitudinal changes in functional disability in Alzheimer's disease patients. Int Psychogeriatr 25: 929-937.

287. Blesa R (2000) Galantamine: therapeutic effects beyond cognition. Dement Geriatr Cogn Disord 11: 28-34.

288. Blesa R, Davidson M, Kurz A, Reichman W, van Baelen B, Schwalen S. (2003) Galantamine provides sustained benefits in patients with 'advanced moderate' Alzheimer's disease for at least 12 months. Dement Geriatr Cogn Disord 15: 79-87.

289. Farlow M, Cyrus, PA (2000) Metrifonate therapy in Alzheimer's disease: a pooled analysis of four randomized, double-blind, placebo-controlled trials. Dement Geriatr Cogn Disord 11: 202-211.

290. Raskind M, Peskind ER, Wessel T, Yuan W (2000) Galantamine in AD: A 6-month randomized, placebocontrolled trial with a 6-month extension. The Galantamine USA-1 Study Group. Neurology 54: 2261-2268.

291. Gelinas I, Gauthier S, Cyrus PA (2000) Metrifonate enhances the ability of Alzheimer's disease patients to initiate, organize, and execute instrumental and basic activities of daily living. J Geriatr Psychiatry Neurol 13: 9-16.

292. Rockwood K, Fay S, Song X, MacKnight C, Gorman M, (2006) Attainment of treatment goals by people with Alzheimer's disease receiving galantamine: a randomized controlled trial. CMAJ 174: 1099-1105.

293. Pfeffer R, Kurosaki TT, Harrah CH, Jr, et al (1982) Measurement of functional activities in older adults in the community. J Gerontol 37: 323–329.

294. Teng E, Becker, B. W., Woo, E., Knopman, D. S., Cummings, J. L., & Lu, P. H. (2010) Utility of the Functional Activities Questionnaire for distinguishing mild cognitive impairment from very mild Alzheimer’s disease. Alzheimer Dis Assoc Disord 24: 348.

295. Teunisse S, Derix MM (1997) The interview for deterioration in daily living activities in dementia: agreement between primary and secondary caregivers. Int Psychogeriatr 9: 155-162.

296. Teunisse S, Derix, MM (1991) Measurement of activities of daily living in patients with dementia living at home: development of a questionnaire. Tijdschr Gerontol Geriatr 22: 53-59.

297. Böhm P, Peña-Casanova J, Aguilar M, Hernández G, Sol JM, Blesa R (1998) Clinical validity and utility of the interview for deterioration of daily living in dementia for Spanish-speaking communities. Int Psychogeriatr 10: 261-270.

298. Voigt-Radloff S, Leonhart R, Schützwohl M, Jurjanz L, Reuster T, Gerner A, et al (2012) Interview for Deterioration in Daily Living Activities in Dementia: construct and concurrent validity in patients with mild to moderate dementia. Int Psychogeriatr 24: 382-390.

299. Katz S, Ford AB, Moskowitz RW, Jackson BA, Jaffe MW (1963) Studies of illness in the aged. The Index of ADL: a standardized measure of biological and psychosocial function. JAMA 185: 914-919.

300. Desai A, Grossberg GT, Sheth DN (2004) Activities of Daily Living in patients with Dementia. CNS drugs 18: 853-875.

301. Londos E, Passant, U., Brun, A., Gustafson, L. (2000) Clinical Lewy body dementia and the impact of vascular components. Int J Geriatr Psychiatry 15: 40-49.

302. Agüero-Torres H, Fratiglioni, L., Guo, Z., Viitanen, M., von Strauss, E., Winblad, B. (1988) Dementia is the major cause of functional dependence in the elderly: 3-year follow-up data from a population-based study. Am J Public Health 88: 1452-1456.

303. Katz S, Ford A, Moskowitz R, et al (2000) Katz index of activities of daily living. In: Handbook of psychiatric measures. Washington, DC: American Psychiatric Association. 130–131 p.

304. Hokoishi K, Ikeda M, Maki N, Nomura M, Torikawa S, Fujimoto N, et al (2001) Interrater reliability of the physical self-maintenance scale and instrumental activities of daily living scale in a variety of health professional representatives. Aging Ment Health 5: 38–40.

305. Lawton M, Brody EM (1969) Assessment of older people: self-maintaining and instrumental activities of daily living. Gerontologist 9: 179–186.

306. Ng TP, Niti, M., Chiam, P. C., & Kua, E. H. (2006) Physical and cognitive domains of the instrumental activities of daily living: validation in a multiethnic population of Asian older adults. J Gerontol A Biol Sci Med Sci 61: 726-735.

307. Sikkes SAM, De Lange-de Klerk, E. S. M., Pijnenburg, Y. A. L., Scheltens, P (2009) A systematic review of Instrumental Activities of Daily Living scales in dementia: room for improvement. J Neurol Neurosurg Psychiatry 80: 7-12.

308. Feldman H, Gauthier, S., Hecker, J., Vellas, B., Emir, B., Mastey, V., et al (2003) Efficacy of donepezil on maintenance of activities of daily living in patients with moderate to severe Alzheimer's disease and the effect on caregiver burden. J Am Geriatr Soc 51: 737-744.

309. Green CR, Mohs, R. C., Schmeidler, J., Aryan, M., & Davis, K. L. (1993) Functional decline in Alzheimer's disease: a longitudinal study. J Am Geriatr Soc 41: 654-661.

310. Oswald W, Fleischmann, UM (1995) Nürnberger-Alters-Inventar (NAI)-testmanual und-textband Hogrefe, Göttingen.

311. Zank S, & Frank, S. (2002) Family and professional caregivers' ratings of dementia symptoms and activities of daily living of day care patients: do differences change over time? Aging Ment Health 6: 161-165.

312. Schneider LS (1997) Validity and reliability of the Alzheimer disease cooperative study-clinical global impression of change. Alzheimer Dis Assoc Disord 11: 22-32.

313. Schneider LS, Clark, C. M., Doody, R., Ferris, S. H., Morris, J. C., Raman, R., et al (2006) ADCS Prevention Instrument Project: ADCS-clinicians' global impression of change scales (ADCS-CGIC), self-rated and study partner-rated versions. Alzheimer Dis Assoc Disord 20: 124-138.

314. Guy W (1976) Clinical Global Impression Scale. ECDEU Assessment Manual for Psychopharmacology - Revised. Rockville, MD.

315. Schneider L, Olin JT (1996) Clinical global impressions in Alzheimer's clinical trials. Int Psychogeriatr 8: 277-288.

316. Homma A, Nakamura, Y., Kobune, S., Haraguchi, H., Kodani, N., Takami, I., et al. (2006) Reliability study on the Japanese version of the Clinician’s Interview-Based Impression of Change. Dement Geriatr Cogn Disord 21: 97-103.

317. Knopman D, Knapp MJ, Gracon SI, Davis CS (1994) The Clinician Interview-Based Impression (CIBI): A clinician’s global change rating scale in Alzheimer’s disease. Neurology 44: 2315-2321.

318. Nakamura Y, Usui M, Nishikawa T, Takita M, Shigeta M, Imai Y, et al (2012) CIBIC Plus-J Assessment Using a Videotaped Method in Alzheimer’s Disease Patients. Dement Geriatr Cogn Disord Extra 2: 271-277.

319. Roth M, Tym E, Mountjoy CQ, Huppert FA, Hendrie H, Verma S, et al (1986) CAMDEX. A standardised instrument for the diagnosis of mental disorder in the elderly with special reference to the early detection of dementia. Br J Psychiatry 149: 698-709.

320. Blessed G, Tomlinson, B. E., Roth, M. (1968) The association between quantitative measures of dementia and of senile change in the cerebral grey matter of elderly subjects. Br J Psychiatry 114: 797-811.

321. Lee DY, Yoon, J. C., Lee, K. U., Jhoo, J. H., Kim, K. W., Lee, J. H., et al. (1999) Reliability and validity of the Korean version of Short Blessed Test (SBT-K) as a dementia screening instrument. J Korean Neuropsychiatr Assoc, 38: 1365–1375.

322. Lam LCW, Chiu, H. F. K., Li, S. W., Chan, W. F., Chan, C. K. Y, Wong, M., et al. (1997) Screening for dementia: A preliminary study on the validity of the Chinese version of the Blessed-Roth Dementia Scale. Int Psychogeriatr 9: 39–46.

323. Yang YH, Lai, C. L., Lin, R. T., Tai, C. T., & Liu, C. K. (2006) Cut-off values of Blessed dementia rating scale and its clinical application in elderly Taiwanese. Kaohsiung J Med Sci 22: 377-384.

324. Vajdickova K, Kolibas, E., Heretik, A., & Kosc, M. (1995) Application of behavioural scale in the diagnosis of dementia of advanced age. Ceska a Slovenska Psychiatrie 91: 7-14.

325. Davis P, Morris J, Grant E (1990) Brief screening test versus clinical staging in senile dementia of the Alzheimer type. J Am Geriatr Soc 38: 129-135.

326. Zillmer E, Fowler P, Gutnick HN and Becker E (1990) Comparison of two cognitive bedside screening instruments in nursing home residents: a factor analysis study. J Gerontol 45: 69-74.

327. Landes A, Sperry S and Strauss M (2005) Prevalence of Apathy, Dysphoria and Depression in Relation to Dementia Severity in Alzheimer’s Disease. J Neuropsychiatry Clin Neurosci 17: 342-349.

328. Villardita C, Lomeo C (1992) Alzheimer’s disease: correlational analysis of three screening tests and three behaviour scales. Acta Neurol Scand 80: 603-608.

329. Kawas C, Karagiozis, H., Resau, L., Corrada, M., & Brookmeyer, R. (1995) Reliability of the blessed telephone information-memory-concentration test. J Geriatr Psychiatry Neurol 8: 238-242.

330. Clark CM, Ewbank, D.C. (1996) Performance of the dementia severity rating scale: a caregiver questionnaire for rating severity in Alzheimer disease. Alzheimer Dis Assoc Disord 10: 31-39.

331. Xie SX, Ewbank, D. C., Chittams, J., Karlawish, J. H., Arnold, S. E., & Clark, C. M. (2009) Rate of decline in Alzheimer's disease measured by a dementia severity rating scale. Alzheimer Dis Assoc Disord 23: 268.

332. Newberg A, Cotter A, Udeshi M, Alavi A, Clark C (2003) A Metabolic Imaging Severity Rating Scale for the Assessment of Cognitive Impairment. Clin Nucl Med 28: 565-570.

333. Karlawish J, Casarett DJ, James BD, Tenhave T, Clark CM, Asch DA (2003) Why would caregivers not want to treat their relative’s Alzheimer’s Disease? J Am Geriatr Soc 51: 1391-1397.

334. Gottfries C, Brane G, Gullberg B, Steen G. (1982) A new rating scale for dementia syndromes. Arch Gerontol Geriatr 1: 311–330.

335. Bråne G, Gottfries, C. G., & Winblad, B. (2000) The Gottfries-Bråne-Steen scale: validity, reliability and application in anti-dementia drug trials. Dement Geriatr Cogn Disord 12: 1-14.

336. Robert P, Ferris, S., Gauthier, S., Ihl, R., Winblad, B., & Tennigkeit, F. (2010) Review of Alzheimer’s disease scales: is there a need for a new multi-domain scale for therapy evaluation in medical practice. Alzheimers Res Ther 2: 24.

337. Shader R, Harmaz JS, Salzman C (1974) A new scale for clinical assessment in geriatric populations: Sandoz Clinical Assessment - Geriatric (SCAG). J Am Geriatr Soc 22: 107-103.

338. Venn R (1983) The Sandoz Clinical Assessment-Geriatric (SCAG) scale. Gerontology 29: 185-198.

339. Herrmann W, Stephan K, Gaede K, Apeceche M. (1996) A Multicenter Randomized Double-Blind Study on the Efficacy and Safety of Nicergoline in Patients with Multi-lnfarct Dementia. Dement Geriatr Cogn Disord 8: 9-17.

340. Gräsel E (2002) When home care ends—changes in the physical health of informal caregivers caring for dementia patients: a longitudinal study. J Am Geriatr Soc 50: 843-849.

341. Hughes C, Berg L, Danziger WL, Coben LA, Martin RL (1982) A new clinical scale for the staging of dementia. Br J Psychiatry 140: 566-572.

342. O’Bryant SE, Lacritz, L. H., Hall, J., Waring, S. C., Chan, W., Khodr, Z. G., et al (2010) Validation of the new interpretive guidelines for the clinical dementia rating scale sum of boxes score in the national Alzheimer's coordinating center database. Arch Neurol 67: 746-749.

343. Macedo Montaño MBM, & Ramos, L. R (2005) Validity of the Portuguese version of clinical dementia rating. Revista de saúde pública 39: 912-917.

344. Chaves MLF, Camozzato, A. L., Godinho, C., Kochhann, R., Schuh, A., De Almeida, V. L., et al (2007) Validity of the clinical dementia rating scale for the detection and staging of dementia in Brazilian patients. Alzheimer Dis Assoc Disord 21: 210-217.

345. Berg L, Miller JP, Baty J, Rubin EH, Morris JC, Figiel G (1992) Mild senile dementia of the Alzheimer type. 4. Evaluation of intervention. Ann Neurol 31: 242-249.

346. Zemlan F (1996) Velnacrine for the treatment of Alzheimer's disease: a double-blind, placebo-controlled trial. The Mentane Study Group. J Neural Transm 103: 1105-1116.

347. Sano M, Ernesto C, Thomas RG, Klauber MR, Schafer K, Grundman M, et al (1997) A controlled trial of selegiline, alpha-tocopherol, or both as treatment for Alzheimer's disease. The Alzheimer's Disease Cooperative Study. N Engl J Med 336: 1216-1222.

348. Jones R, Soininen H, Hager K, Aarsland D, Passmore P, Murthy A, et al (2004) A multinational, randomised, 12-week study comparing the effects of donepezil and galantamine in patients with mild to moderate Alzheimer's disease. Int J Geriatr Psychiatry 19: 58-67.

349. Rockwood K (2004) Size of the treatment effect on cognition of cholinesterase inhibition in Alzheimer's disease. J Neurol Neurosurg Psychiatry 75: 677-685.

350. Cortes F, Gillette-Guyonnet S, Nourhashemi F, Andrieu S, Cantet C, Vellas B (2005) Recent data on the natural history of Alzheimer's disease: results from the REAL. J Nutr Health Aging 9: 86-93.

351. McLendon B, Doraiswamy PM (1999) Defining meaningful change in Alzheimer's disease trials: the donepezil experience. J Geriatr Psychiatry Neurol 12: 39- 48.

352. Tariot P, Cummings JL, Katz IR, Mintzer J, Perdomo CA, Schwam EM, Whalen E. (2001) A randomized, double-blind, placebo-controlled study of the efficacy and safety of donepezil in patients with Alzheimer's disease in the nursing home setting. J Am Geriatr Soc 49: 1590-1599.

353. Imbimbo B, Troetel WM, Martelli P, Lucchelli F (2000) A 6-month, double-blind, placebo-controlled trial of eptastigmine in Alzheimer's disease. Dement Geriatr Cogn Disord 11: 17-24.

354. Rogers S, Farlow MR, Doody RS, Mohs R, Friedhoff LT (1998) A 24-week, double-blind, placebo-controlled trial of donepezil in patients with Alzheimer's disease. Neurology 50: 136-145.

355. Burns A, Rossor M, Hecker J, Gauthier S, Petit H, Möller HJ, et al (1999) The effects of donepezil in Alzheimer's disease - results from a multinational trial. Dement Geriatr Cogn Disord 10: 237-244.

356. Summers W, DeBoynton V, Marsh GM, Majovski LV (1990) Comparison of seven psychometric instruments used for evaluation of treatment effect in Alzheimer's dementia. Neuroepidemiology 9: 193-207.

357. Roman G, Wilkinson DG, Doody RS, Black SE, Salloway SP, Schindler RJ (2005) Donepezil in vascular dementia: combined analysis of two large-scale clinical trials. Dement Geriatr Cogn Disord 20: 338-344.

358. Riepe M, Adler G, Ibach B, Weinkauf B, Gunay I, Tracik F. (2006) Adding memantine to rivastigmine therapy in patients with mild-to moderate Alzheimer's disease: Results of a 12-week, open-label pilot study. Prim Care Companion J Clin Psychiatry 8: 258-263.

359. Tractenberg R, Schafer K and Morris JC (2001) Interobserver disagreements on clinical dementia rating assessment: interpretation and implications for training. Alzheimer Dis Assoc Disord 15: 155-161.

360. Tractenberg R, Weiner MF, Cummings JL, Patterson MB, Thal LJ (2005) Independence of changes in behavior from cognition and function in community-dwelling persons with Alzheimer's disease: a factor analytic approach. J Neuropsychiatry Clin Neurosci 17: 51-60.

361. Waite L, Grayson D, Jorm AF, Creasey H, Cullen J, Bennett H, et al (1999) Informant-based staging of dementia using the clinical dementia rating. Alzheimer Dis Assoc Disord 13: 34-37.

362. Rockwood K, Strang D, MacKnight C, Downer R, Morris JC. (2000) Interrater reliability of the Clinical Dementia Rating in a multicenter trial. J Am Geriatr Soc 48: 558-559.

363. O'Connor D, Blessed G, Cooper B, Jonker C, Morris JC, Presnell IB, et al (1996) Cross-national interrater reliability of dementia diagnosis in the elderly and factors associated with disagreement. Neurology 47: 1194-1199.

364. Marin D, Flynn S, Mare M, Lantz M, Hsu MA, Laurans M, et al (2001) Reliability and validity of a chronic care facility adaptation of the Clinical Dementia Rating scale. Int J Geriatr Psychiatry 16: 745-750.

365. Morris J, Ernesto C, Schafer K, Coats M, Leon S, Sano M, et al (1997) Clinical dementia rating training and reliability in multicenter studies: the Alzheimer's Disease Cooperative Study experience. Neurology 48: 1508-1510.

366. McCulla M, Coats M, Van Fleet N, Duchek J, Grant E, Morris JC (1989) Reliability of clinical nurse specialists in the staging of dementia. Arch Neurol 46: 1210-1211.

367. Haroutunian V, Perl DP, Purohit DP, Marin D, Khan K, Lantz M, et al (1998) Regional distribution of neuritic plaques in the nondemented elderly and subjects with very mild Alzheimer disease. Arch Neurol 55: 1185-1191.

368. Burke W, Miller JP, Rubin EH, Morris JC, Coben LA, Duchek J, et al (1988) Reliability of the Washington University Clinical Dementia Rating. Arch Neurol 45: 31-32.

369. Choi SH, Na, D. L., Lee, B. H., Hahm, D. S., Jeong, J. H., Jeong, Y., et al (2002) The validity of the Korean version of Global Deterioration Scale. J Korean Neurol Assoc 20: 612-617.

370. Reisberg B (2007) Global measures: utility in defining and measuring treatment response in dementia. Int Psychogeriatr 19: 421-456.

371. Solomon P, Adams FA, Groccia ME, DeVeaux R, Growdon JH, Pendlebury WW. (1999) Correlational Analysis of Five Commonly Used Measures of Mental Status/Functional Abilities in Patients with Alzheimer Disease. Alzheimer Dis Assoc Disord 13: 147-150.

372. Solomon TM, Budson, A. E., Mirkovic, N., Murphy, C. A., & Solomon, P. R. (2014) Correlational Analysis of 5 Commonly Used Measures of Cognitive Functioning and Mental Status An Update. Am J Alzheimers Dis Other Demen 29: 718-722.

373. Eisdorfer C, Cohen D, Paveza GJ, Ashford JW, Luchins DJ, Gorelick PB, et al. (1992) An empirical evaluation of the Global Deterioration Scale for Staging Alzheimer’s Disease. Am J Psychiatry 149: 190-194.

374. Reisberg B, Franssen EH, Bobinski M, Auer S, Monteiro I, Boksay I, et al (1996) Overview of methodologic issues for pharmacological trials in mild, moderate and severe Alzheimer’s Disease. Int Psychogeriatr 8: 159-193.

375. Auer S, Reisberg B (1996) Reliability of the Modified Ordinal Scales of Psychological Development: a cognitive assessment battery for severe dementia. Int Psychogeriatr 8: 225-231.

376. Choi S, Lee BH, Kim S, Hahm DS, Jeong JH, Yoon SJ, et al (2003) Interchanging scores between Clinical Dementia Rating Scale and Global Deterioration Scale. Alzheimer Dis Assoc Disord 17: 98-105.

377. Bobinski M, De Leon MJ, Wegiel J, Desanti S, Convit A, Saint Louis LA, et al (1999) The histological validation of post mortem magnetic resonance imaging-determined hippocampal volume in Alzheimer's disease. Neuroscience 95: 721-725.

378. Jack C, Dickson DW, Parisi JE, Xu YC, Cha RH, O’brien PC, et al (2002) Antemortem MRI findings correlate with hippocampal neuropathology in typical aging and dementia. Neurology 58: 750-757.

379. Apostolova L, Zarow C, Biado K, Hurtz S, Boccardi M, Somme J, et al (2015) Relationship between hippocampal atrophy and neuropathology markers: A 7T MRI validation study of the EADC-ADNI Harmonized Hippocampal Segmentation Protocol. Alzheimers Dement 11: 139-150.

380. O’Brien J, Paling S, Barber R, Williams ED, Ballard C, McKeith IG, et al (2001) Progressive brain atrophy on serial MRI in dementia with Lewy bodies, AD, and vascular dementia. Neurology 56: 1386-1388.

381. Jack C, Shiung MM, Gunter JL, O’brien PC, Weigand SD, Knopman DS, et al (2004) Comparison of different MRI brain atrophy rate measures with clinical disease progression in AD. Neurology 62: 591-600.

382. Fox N, Scahill RI, Crum WR, Rossor MN (1999) Correlation between rates of brain atrophy and cognitive decline in AD. Neurology 52: 1687.

383. Henneman W, Sluimer JD, Barnes J, Van Der Flier WM, Sluimer IC, Fox NC, et al (2009) Hippocampal atrophy rates in Alzheimer disease Added value over whole brain volume measures. Neurology 72: 999-1007.

384. Sluimer J, Vrenken H, Blankenstein MA, Fox NC, Scheltens P, Barkhof F, et al (2008) Whole-brain atrophy rate in Alzheimer disease Identifying fast progressors. Neurology 70: 1836-1841.

385. Sluimer J, Bouwman FH, Vrenken H, Blankenstein MA, Barkhof F, van der Flier WM, et al (2010) Whole-brain atrophy rate and CSF biomarker levels in MCI and AD: a longitudinal study. Neurobiol Aging 31: 758-764.

386. Jack C, Lowe VJ, Weigand SD, Wiste HJ, Senjem ML, Knopman DS, et al (2009) Serial PIB and MRI in normal, mild cognitive impairment and Alzheimer's disease: implications for sequence of pathological events in Alzheimer's disease. Brain 132: 1355-1365.

387. Archer H, Edison P, Brooks DJ, Barnes J, Frost C, Yeatman T, et al (2006) Amyloid load and cerebral atrophy in Alzheimer's disease: An 11C‐PIB positron emission tomography study. Ann Neurol 60: 145-147.

388. Marshall J, Martin T, Downie J, Malisza K (2007) A comprehensive analysis of MRI research risks: in support of full disclosure. Can J Neurol Sci 34: 11-17.

389. Shellock F, Crues JV (2004) MR procedures: biologic effects, safety, and patient care. Radiology 232: 635-652.

390. Harris L, Robinson J, Menzies RG (1999) Evidence for fear of restriction and fear of suffocation as components of claustrophobia. Behav Res Ther 37: 155-159.

391. Katz R, Wilson L, Frazer N (1994) Anxiety and its determinants in patients undergoing magnetic resonance imaging. J Behav Ther Exp Psychiatry 25: 131-134.
