# Supplementary material for: Core outcome measures for interventions to prevent or slow the progress of dementia for people living with mild to moderate dementia: Systematic review and consensus recommendations
Source: PLoS One. 2017 Jun 29;12(6):e0179521. doi: 10.1371/journal.pone.0179521 (PMC5491018; doi:10.1371/journal.pone.0179521)
Supplement: S2 File — (PDF) [file pone.0179521.s003.pdf]

UCL

Development of a consensus standardised 'Core' Outcome Data set for disease modification in clinical trials for mild to moderate dementia (COD Dementia)

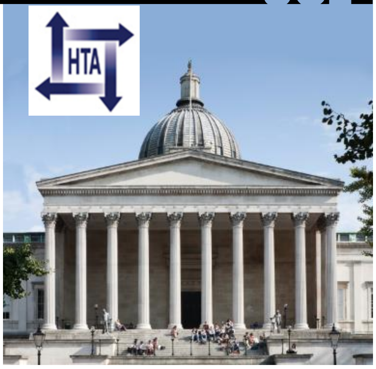

Gill Livingston

UCL

Quick recap

Why
Who
How
Purpose of today
Publications plan

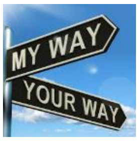

August, 2014

Why?

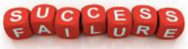

- Research knowledge and funding on dementia lags behind other major diseases such as cancer or heart disease
- No disease modification treatment
- NHS has 100000 people diagnosed/annum
- Huge potential to research effectively
- Trials developed without liaison and with results not comparable or meta-analysable
- This project is to generate evidence based, consensus so that easier to get funding and results.

UCL

Who? The community

We invited **everyone** HTA invited and some they had left out

- Gill Livingston (CI) with Rob Howard leading
- Charlotte Roberts, Jenny McCleery, Louise Lafortune and Gail Mountain as co-applicants contributing relevant work their groups had done
- James Pickett from Alzheimer's Society leading PPI
- The researchers:
- Lucy Webster Research assistant
  - Derek Groskreutz (volunteer UCL/Yale masters student)
  - Anna Grinbergs- Saull (AS)

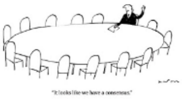

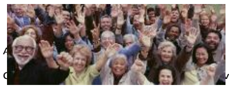

A[illegible] Occupational therapy - Gail Mountain (co-applicant)

C[illegible]vement Group- Jenny McCleery (co-applicant)

Dementia care - Frances Bunn, Claire Goodman

Dementia pharmacist - Ian Maidment

Health service research - Sasha Shepperd

Health Outcome Measurement - Sallie Lamb, Charlotte Roberts (co-applicant),

Neurology - Peter Garrard

Old age medicine - Patrick Kehoe, Roy Jones, Peter Passmore, John Young

Old age psychiatry - Clive Ballard, Sube Banerjee, Alistair Burns, Chris Fox, Clive Holmes, Rob Howard(co-applicant), Gill Livingston (PI), John O'Brien, Robert Perneczky

Palliative medicine - Fliss Murtagh

Primary care dementia research - Louise Robinson

Psychology and dementia - Linda Clare, Georgina Charlesworth, Murna Downs, Esme Moniz- Cook, Bob Woods

Public Health and ageing - Carol Brayne , Louise Lafortune(co-applicant)

Scale measurement –Orlaith Burke

Social care and social policy - David Challis, Katie Featherstone, Justine Schneider, Claire Surr

UCL

–

How: Very rapidly. No mission creep.

Work package 1 Use of current knowledge

Jenny McCleery Cochrane register

Charlotte Roberts - International Consortium for Health Outcomes Measurement (ICHOM) Dementia Working Group what matters for patients

Louise Lafortune – AS systematic review of non-pharmacological outcomes in dementia

Gail Mountain (Esme Moniz- Cooke) JPND psychosocial measures

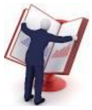

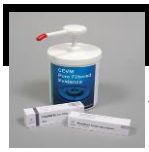

Workpackage 2 and 3

WP 2 – systematic review + literature from WPI

Identify relevant trials on disease modification (search tweaked from application)

Extract , tabulate and count the use of each measure

Find validation data

Workpackage 3

An assessment of the importance of the ‘proposed’ core outcome measures to patients and carers

Three groups: Sheffield, London, Cambridge

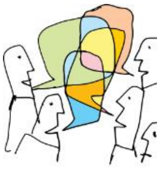

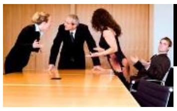

Today- workpackage 4

Consensus conference

August, 2014

Information

- Lucy – systematic review
- Anna –focus groups
- Champions summarising their areas in review.
  - Is it core?
  - Proposing outcome measures and explaining why
  - Discussion in between
- Rob- cognition
- John- neuroimaging
- Robert – CSF and blood tests
- Gail ADL
- Gill-neuropsychiatric
- Sube – QOL
- Bob-global

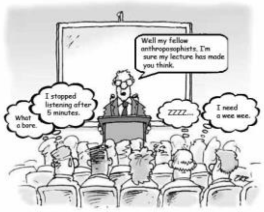

Measures for use in all NIHR applications for trials disease modification in mild to moderate dementia

What is core?

What measures should we use?

… and why?

Transparency

Putting it all together

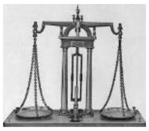

What next

HTA publication- second week of June
Submission to Lancet Neurology
Gill and Lucy will write (plus any volunteers)
Use words from champions
We will circulate

Next step after
?Work with others including ARUK - with regulators and pharmaceutical industry
? Widen remit
Thanks for attention

COD Dementia: Systematic Review

Lucy Webster

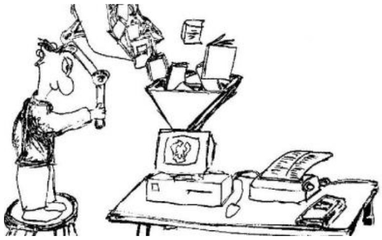

Purpose

- A "brief" systematic review to see what outcome measures are used across previous disease modification trials
- We defined a disease modifying treatment as one that is *"trying to change the underlying pathology of the disease of dementia"*

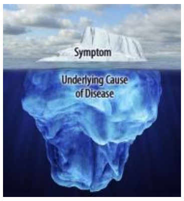

Search terms

- Adapted from search strategy in application
- Also adapted dependent on database e.g. more basic search on trial registers

Dementia related search terms **AND** Outcome related search terms **AND** Intervention OR therap* OR trial* **AND** Control*

- Limited searches to English when possible

Searches

Workstream 1:
- ALOIS
- References from Louise La Fortune's project

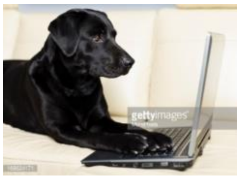

Workstream 2:
- Cochrane central register of controlled trials
- Medline
- PsycInfo
- Embase
- Lilacs
- CINAHL
- ISRCTN
- Clinicaltrials.gov
- Hand searching of relevant systematic reviews in the database

Altogether: 37787 references

Screening titles and abstracts

22,918 abstracts with duplicates removed

- Derek and I screened first 20 to check for consistency
- We were looking for trials that appeared that they could be about disease modifying treatments in mild to moderate dementia from the abstracts
- From screening we wanted to look at **897 full texts**

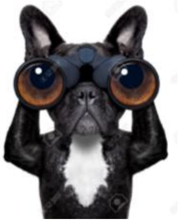

Screening full texts

- A, D and L screened first 10 independently, compared answers and discussed, then same process with next 10.

Inclusion criteria:
- Paper in English
- Peer reviewed journal article or ongoing trial
- Mild or moderate dementia
- Disease modification trials
- Randomised controlled trial or Clinical controlled trial
- At least 1 Quantitative outcome

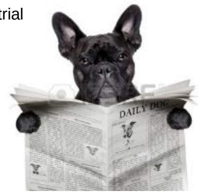

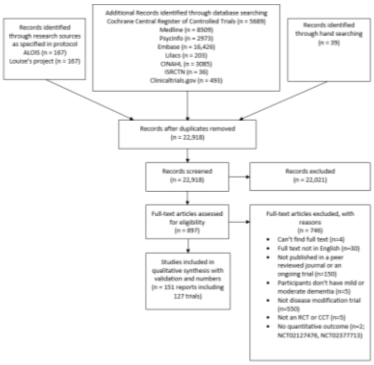

Full texts excluded

746 Full-text articles excluded:

- 4 can't find full text
- Full text not in English
- Not published in a peer reviewed journal or an ongoing trial
- No participants with mild or moderate dementia
- Not a disease modification trial
- Not an RCT or CCT
- No quantitative outcome

Included 127 trials (from 151 reports)

Data extraction

Data extracted from first 5 trials independently by D and L and compared.

Also used to test extraction spreadsheet.

Extracted data about:

- Location, dementia type & severity, how dementia diagnosis made.
- Participants sex & age
- Intervention
- Control group
- Which outcomes primary or secondary and when measured

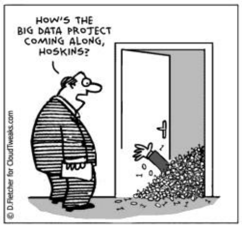

Measures

- Divided measures cross 6 domains: Cognition, Activities of daily living, quality of life, global, behavioural, and biological markers.
- Overall 80 outcome measures:
  - 71 different questionnaire/interview measures
  - 9 biological techniques used e.g. MRI, EEG, blood
- Recorded frequency of use and with how many participants

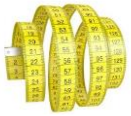

Validation of measures

- Basic validation as very limited time!
- Looking for information that is available

- Who the measure is valid for use with and languages its available in
- Sensitivity to change in treatment studies
- Reliability
- Acceptability
- Floor and ceiling effects
- Minimal clinically important difference

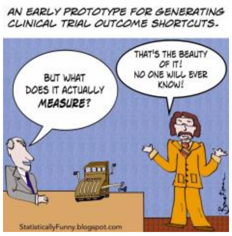

Core Outcomes for Dementia
Patient & Public Involvement

Anna Grinbergs-Saull
Research Engagement Officer
Alzheimer's Society

Patient & Public Involvement (PPI)

**Focus Group 1**	ADL, Behaviour, Cognition, Quality of Life
**Focus Group 2**	global measures, outcome measure packages
**Focus Group 3**	biomarkers, imaging, outcome measure packages

- **12 Volunteers**
  - 3 People with Dementia
  - 2 Carers
  - 6 Former Carers
  - 1 PPIE group member
- **Wide range of backgrounds**
  - Alzheimer's, FTD, Vascular Dementia, PCA
  - Trial participants
  - People without research experience

What should be Core?

- **Broad Support for each domain**
- **Package: Biomarkers, cognition, behavioural, (global?)**
- **Impact of context and environment**

Recommendations: Cognition

Positives
- Measures key signs of progression
- ADAS-Cog has good amount of detail

Negatives
- Strong reaction against MMSE – irrelevant/restricted
- Memory tests seen as demoralising

"watching someone fail a test"

- Memory not always a symptom

Recommendations: Biomarkers

Positives
- Most objective and reliable measure
- Tangible contribution
- CSF strongly supported
- Blood tests common-place and unproblematic
- Imaging generally accepted as feasible

Negatives
- MRI and PET not feasible in vascular dementia
- Travel and location

Recommendations: Behavioural

Positives
- Often significant aspect of dementia
- More sensitive than e.g. ADL

Negatives
- Not sensitive enough in isolation
- Less applicable in mild-moderate dementia
- Lacks detail – reason behind changes
- Missing important behaviours e.g. change in tastes

Recommendations: Activities of Daily Living

Positives
- PwD: gives an accurate, practical account
- IADL more relevant
- Less distressing than cognition

Negatives
- Katz/ yes-no scales lack detail
- Restricted to certain activities and environments
- Assistance not always from carer

> *"I wont remember to take my medication without my alarm going off.. I can remember with the help of other things"*

- Requires recall
- Resembles benefits questions

Recommendations: Quality of Life

Positives
- Could be core – important to assess
- DEM-QOL is comprehensive and easy to use
- EQ-5D thermometer is "all-encompassing"

Negatives
- Assessing someone else's QoL
- Relies on accurate interpretation of responses
- EQ-5D & QoL-AD lack detail
- Does not account for personality

Recommendations: Global

Positives
- Could be core
- Broad measure – gives holistic account

Negatives
- Shouldn't be core – too superficial
- Depends on individual's experience on the day
- Larger package would give detailed holistic view

Recommendations & Priorities

- Involving people without defined carer
- Time: long meeting vs. long day
- Travel is significant barrier
- Relevance of measures
- Sense of purpose and contribution are vital

Summary

- Core Package: Biological, cognition and behaviour
- More weight on Biomarkers
- Cognitive tests have greater impact on individual
- Prioritise usefulness of the measure
- Consider impact beyond physical risk (e.g. travel/ relevance)
- Different dementias, different measures?

Cognition outcome measures for disease modifying trials in AD

Robert Howard, UCL

The Alzheimer's Disease Assessment Scale – Cognitive Subscale (ADAS-Cog) and the Mini-Mental State Examination (MMSE)

ADAS-Cog	MMSE
91 trials 20,005 participants	66 trials 17,237 participants
45 minutes	10 minutes
Special training	Widely clinically used
Scored out of 70	Scored out of 30
Can be augmented with Delayed Word Recall, Maze Task, Digit Cancellation and subjective judgement of concentration and distractability	

The Alzheimer's Disease Assessment Scale – Cognitive Subscale (ADAS-Cog) and the Mini-Mental State Examination (MMSE)

Both poor at distinguishing early AD/MCI from health

Both relatively insensitive to change in very mild AD

But both demonstrated excellence in detection of treatment effects in cholinesterase inhibitor and memantine trials

Both have published minimum clinically important differences (1.4 MMSE points probably means more to clinicians than 3 ADAS-Cog points)

If trials can be designed to anticipate differences of this magnitude, either scale could be used

Neuropsychological Test Battery (NTB)

7 trials 3180 participants (Cog State 161 participants, CERAD 80)

Developed because of floor/ceiling effects with cognitive measures

9 measures of cognitive performance, chosen to assess delayed verbal recall and executive functioning:

Wechsler Memory Scale - visual immediate
Wechsler Memory Scale – verbal immediate
Rey Auditory Verbal Learning Test – immediate
Wechsler Memory Scale – digit span
Controlled Word Association Test
Category Fluency Test
Wechsler Memory Scale – visual delayed
Wechsler Memory Scale – verbal delayed
Rey Auditory Verbal Learning Test – delayed

Probably most useful in short trials where proof of concept/signal needed quickly but clinical significance less important

Conclusions

Both ADAS-Cog and MMSE have utility

Choice will depend on available resources, including who is available to conduct assessments

Little evidence that ADAS-Cog superior in detection of treatment effects when present

For short trials, where detection of some kind of signal is the priority, NTB may have a place

Activities of daily living measures

Gail Mountain
Professor of Health Services Research

What are activities of daily living measures?

- ADL measures: basic every day activities that we all have to do e.g. washing, bathing, dressing
- IADL measures: other activities that are necessary and important for daily living e.g. cooking, shopping, money management

Some newer measures include other activities of daily life such as recreation

Why are they used for studies of disease modifying interventions?

To determine whether a person is able to undertake such activities; whether there has been any change in ability as a result of the intervention

Should this be core?

What measures have we got?

12 were initially identified and 9 then shortlisted for consideration

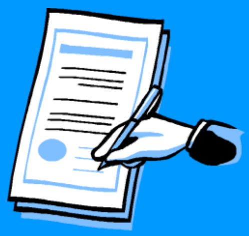

How to identify the best?

- The number of studies where the measure has been used
- The number of participants the measure has been used with
- How good each measure is considered to be based on a number of criteria
- Appropriateness of the items it includes

Quality criteria from the evidence

Developed for use with people with dementia/ has been used with this population

Demonstrated psychometric properties such as;

- Two people can obtain the same result from using it with the same person
- It makes sense to those using it
- It can detect change

What did our PPI group tell us

- Importance of self completion
- Keep it simple
- Difficulty with recall

What does this leave us with?

(1) The Alzheimer's disease cooperative ADL measure; ACDS-ADL
(2) Disability in Dementia measure: DAD
(3) Lawton ADL/ PSMS
(4) Bristol ADL measure: BADLs

The shortlist

Measure	Population intended for	Method of completion
ACDS-ADL	Early/ moderate dementia	Carer interview
DAD	Community living	Carer interview or by task observation
Lawton ADL/PSMS	All stages of dementia when used in combination	By interview with the person with dementia or carer interview
Bristol ADL	Early/ moderate dementia	Carer interview

Recommendations

- The DAD is the most often used for community living people and has an observation option
- The Lawton ADL/ PSMS is the only measure that includes self completion and is appropriate for those with later stage dementia

What challenges remain?

- Carer report versus hearing the voice of the person with dementia
- Methods to capture information; one measure identified used video which is good but impractical

THANK YOU

g.a.mountain@sheffield.ac.uk

Imperial College London

COD dementia: fluid biomarkers

Robert Perneczky
Imperial College London
School of Public Health

Imperial College London

Why development of therapies for dementia fails

- ~2000 registered trials and 900 products in the past 20 years
- Low number of early phase drugs (3.8% vs 31% in cancer)
- 197 products in “active” development
- 216 products suspended or discontinued
- Common reasons: lack of efficacy or safety concerns
- 74% did not report reason for discontinuation
- 3/13 companies were able to provide a reason for discontinuation

Gauthier S et al. (2016) Alzheimers Dement, 12, 60-64

Imperial College London

Definition of a biomarker

- A characteristic that is objectively measured and evaluated as an indicator of normal biologic processes, pathogenic processes or biological responses to a therapeutic intervention
- Any measurable characteristic that is not a clinical assessment
- Clinical measures are those measures that intrinsically are not fully objective

Biomarkers Definitions Working Group (2001) Clinical Pharmacology and Therapeutics, 69, 89-95

Imperial College London

Types of biomarkers

- Prognostic biomarkers
- Predictive biomarkers
- Pharmacodynamic (theragnostic) biomarkers
- Surrogate endpoints

http://www.fda.gov/downloads/Drugs/GuidanceComplianceRegulatoryInformation/Guidances/UCM230597.pdf

Imperial College London

Prognostic biomarkers

- Indicates future clinical course with respect to a defined outcome, in the absence of a specific therapeutic intervention (natural course)
- No relationship to any particular new therapy
- Application of a new therapy may invalidate the pre-therapy inference (marker-clinical association may change with therapy)

http://www.fda.gov/downloads/Drugs/GuidanceComplianceRegulatoryInformation/Guidances/UCM230597.pdf

Imperial College London

Predictive biomarkers

- Measured prior to an intervention
- Identification of individuals susceptible to a drug effect
- Developed for specific therapeutic interventions
- Not necessarily prognostic of post-therapy clinical course

http://www.fda.gov/downloads/Drugs/GuidanceComplianceRegulatoryInformation/Guidances/UCM230597.pdf

Imperial College London

Prognostic vs predictive biomarkers

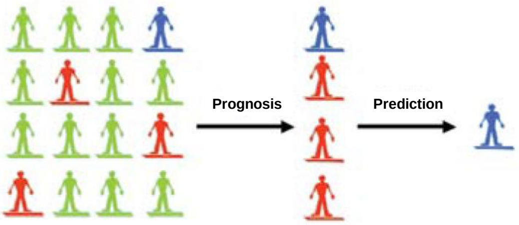

Imperial College London

Pharmacodynamic biomarkers

- Indicate therapy response
- Reveal occurrence or magnitude of biological response
- Developed for specific therapeutic interventions
- May or may not be therapy-specific

http://www.fda.gov/downloads/Drugs/GuidanceComplianceRegulatoryInformation/Guidances/UCM230597.pdf

Imperial College London

Surrogate endpoints

- Subset of pharmacodynamics biomarkers
- Substitute for a clinical endpoint
- Expected to indicate clinical benefit
- Reflects how a study participant feels, functions or survives

http://www.fda.gov/downloads/Drugs/GuidanceComplianceRegulatoryInformation/Guidances/UCM230597.pdf

Imperial College London

Surrogate endpoints

- Subset of pharmacodynamics biomarkers
- Substitute for a clinical endpoint
- Expected to indicate clinical benefit
- Reflects how a study participant feels, functions or survives

http://www.fda.gov/downloads/Drugs/GuidanceComplianceRegulatoryInformation/Guidances/UCM230597.pdf

Imperial College London

CSF biomarkers: overview

- Biomarker signature reflecting neuropathological hallmarks of AD
- Aβ42 ↓ (long before onset of clinical symptoms)
- tTau ↑ (subsequent to Aβ42 decrease)
- pTau181 ↑ (more specific to AD)
- Other amyloid cascade markers for specific purposes (e.g. sAPPβ)
- Used in trials to measure target engagement, sample enrichment, secondary outcomes
- CSF field less advanced for other dementias
- EMA endorses use of CSF markers to enrich MCI populations (high sensitivity and moderate specificity)
- FDA supports use of CSF markers in combination with clinical outcomes in pre-dementia populations

Imperial College London

CSF biomarkers as surrogate endpoints in AD trials

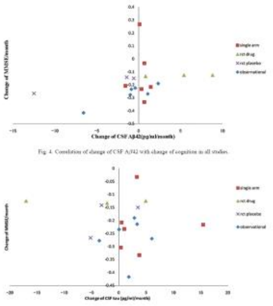

Zhou S et al. (2009) J Alzheimers Dis, 18, 89-102

Imperial College
London

Blood biomarkers

- ????

Domain: Global Functioning

Presenter: Bob Woods

Why global?

- Reflects the nature of the condition
- Combines multiple domains
- (Potentially) combines multiple perspectives (but ? perspective of the person with dementia?)

Measures identified from review

1. Alzheimer's Disease Cooperative Study - Clinical Global Impression of Change
2. Blessed Dementia Rating Scale
3. Clinical Dementia Rating Scale
4. Clinical Global Impressions Scale
5. Clinician's Interview-Based Impression of Change Plus Caregiver Input
6. Dementia Severity Rating Scale
7. Global Deterioration Scale
8. Gottfries-Brane-Steen rating scale for dementia
9. Sandoz Clinical Assessment-Geriatric Scale

Number of trials using each global outcome

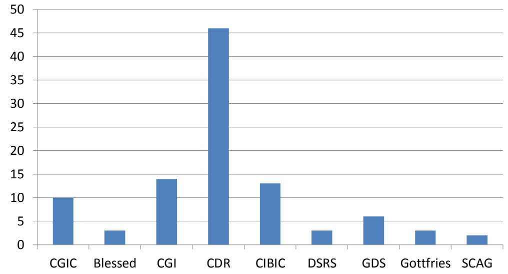

Types of global scales

- Staging of dementia
  - CDR (nb 'sum of boxes' widely used in treatment trials)
  - GDS
- Multiple domain rating
  - DSRS
  - SCAG
  - Blessed Dementia Rating Scale
  - Gottfries-Brane-Steen rating scale for dementia
- 'Impression' – rating change
  - CGI
  - ADCS-CGIC
  - CIBIC Plus

Multiple domain rating scales

- **Dementia Severity Rating Scale**
  - 12 domains, rated by carer
  - Memory; speech and language; ability to make decisions; social & community activity; home activities & responsibilities; eating; control of urination & bowels; personal care & cleanliness; orientation to time and place.
- **Sandoz CAG**
  - 19 domains (5 factors), rated on interview and observation
  - Confusion; mental alertness; self-care; anxiety; hostility; bothersome; irritability; unsociability; fatigue
- **Blessed Dementia Rating Scale**
  - 22 items – often used with Blessed Dementia Information Memory Concentration Test
  - Everyday activities (8 items); ADL – eating, dressing, toilet (3 items); personality changes (11 items)
- **Gottfries-Brane-Steen rating scale for dementia**
  - based on a semi-structured interview and observation of the patient
  - subscales measuring intellectual (12 items), emotional (3 items) activities of daily living (primarily items of self-care) (6 items); behavioural and psychological symptoms of dementia (6 items)

Impression scales - CBIC

- Semi-structured interview, with person with dementia and caregiver
- Four major categories divided into domains
- Probes suggested for each domain, but interviewer may use additional probes
- After completing interview, consult all available information, including MMSE / ADAS-Cog (from that visit)
- Detailed notes on each domain to inform follow-up assessments

General	Mental/Cognitive State	Behaviour	Activities of Daily Living
Relevant History<br>Observation / Evaluation	Arousal / Alertness / Attention / Concentration<br>Orientation<br>Memory<br>Language / Speech<br>Praxis<br>Judgement / Problem Solving / Insight	Thought Content<br>Hallucinations / Delusions / Illusions<br>Behaviour / Mood<br>Sleep / Appetite<br>Neurological / Psychomotor Activity	Basic and Complex (instrumental activities)<br>Social Function

Clinician's Interview Based Impression of Severity (CIBIS) (baseline assessment)

SEVERITY OF ILLNESS

Considering your total clinical experience with this particular population, how mentally ill is this patient now?

0 = Not assessed
1 = Normal, not at all ill
2 = Borderline mentally ill
3 = Mildly ill
4 = Moderately ill
5 = Markedly ill
6 = Severly ill
7 = Among the most extremely ill patients

SCORE ☐

Clinician's Interview Based Impression of Change – CIBIC Plus

THREE – SIX MONTH VISIT

Subject's Interview – Clinical Impression of Change:
- ☐ Very Much Improved
- ☐ Much Improved
- ☐ Minimally Improved
- ☐ No change
- ☐ Minimal worsening
- ☐ Moderate worsening
- ☐ Marked worsening

Informant's Interview – Clinical Impression of Change:
- ☐ Very Much Improved
- ☐ Much Improved
- ☐ Minimally Improved
- ☐ No change
- ☐ Minimal worsening
- ☐ Moderate worsening
- ☐ Marked worsening

Overall Score – Clinical Impression of Change:
- ☐ Very Much Improved
- ☐ Much Improved
- ☐ Minimally Improved
- ☐ No change
- ☐ Minimal worsening
- ☐ Moderate worsening
- ☐ Marked worsening

Inter-rater reliability (selected measures)

- Good to very good across 12 studies for CDR
- Good across 4 studies for GDS
- ? No information for CIBIC or CGIC, but good test re-test reliability claimed for both in one study

Sensitivity to change (selected measures)

- CDR sensitive to treatment effects in 13 studies
- GDS sensitive to change in 2 studies, but not in a third
- CIBIC and CGIC both sensitive to change in 1 study

Recommendation

- IF CIBIC shown to have good inter-rater reliability, the notion of individualising the trajectory of change is worth considering
- Multiple domain rating scales risk not having a rationale for weighting of the various domains – DSRS good content, but note caregiver rating
- Staging scales should be ideal for evaluating disease modifying treatments
  - CDR is widely used and has good reports relating to reliability and sensitivity to change – but if sum of boxes is used, is it any different from a multiple domain scale?
  - Both CDR and CDR-SB do have good discriminant validity (Rikkert et al., 2011)
  - GDS not so widely used, but potentially useful (? With FAST – Functional Assessment Staging)

Biomarkers: Imaging

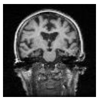
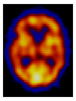
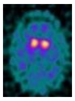
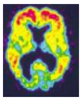

John O'Brien
Professor of Old Age Psychiatry
University of Cambridge

UNIVERSITY OF CAMBRIDGE

Use of imaging in trials of disease modification in AD

- To ensure subject meets diagnostic criteria for AD
- To stratify subject for therapy under study (e.g. amyloid positive, tau positive, WML changes)
- To ensure other inclusion criteria met (e.g. no microbleeds)
- As a safety outcome measure
- To show target engagement (e.g. does amyloid lower)
- As outcome measure in its own right

UNIVERSITY OF CAMBRIDGE

Imaging modalities used in clinical trials

- Computed tomography (CT) - only really used as inclusion/ exclusion criteria for entry
- Structural Magnetic resonance imaging (MRI)
- Perfusion (HMPAO) SPECT/ Metabolic (FDG) PET
- Amyloid (PIB, florbetapir, flutemetamol, flurbetaben) PET
- Tau (AV1451, PBB3, THK) PET
- Other: EEG, Transcranial doppler, MR Spectroscopy

UNIVERSITY OF CAMBRIDGE

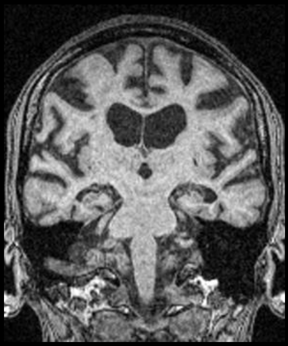

Baseline

Fox and Freeborough, 1996

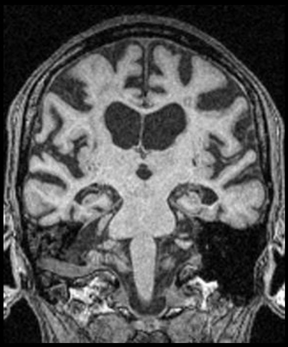

Year 1

Fox and Freeborough, 1996

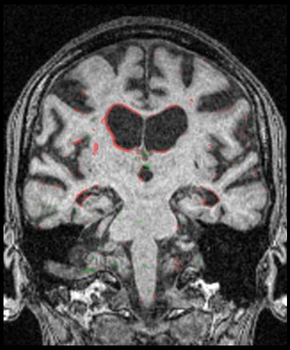

Difference

Fox and Freeborough, 1996

Serial MR Imaging in AD and DLB

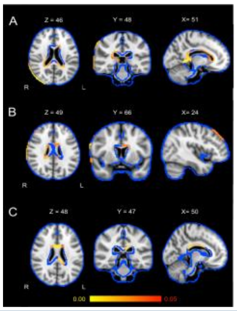

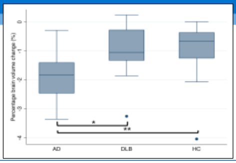

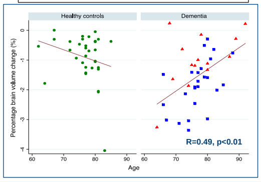

Mak et al, Neuroimage Clinical, 2015

FDG PET significantly superior to Perfusion SPECT. Direct comparison in 100 subjects

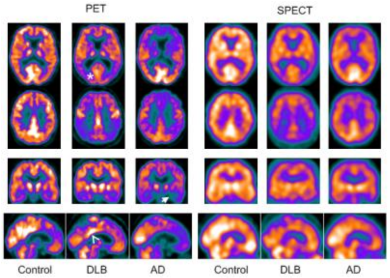

Diagnostic accuracy PET 20% higher than SPECT (93% v 72%)

PET had 5X more significant voxels compared to SPECT (40% v 7%, p<0.0001)

O'Brien et al, Journal of Nuclear Medicine (2014)

Amyloid PET imaging

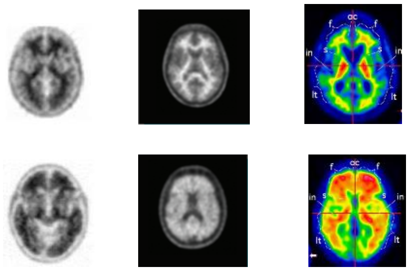

Negative scan: normal

Positive scan: amyloid

Flurbetapir (Amyvid) | **Flurbetaben (NeuraCeq)** | **Flutametamol (Vizamyl)**

Amyloid imaging in Dementia

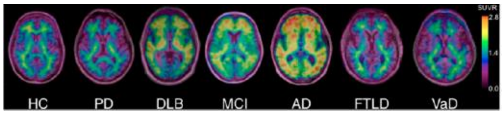

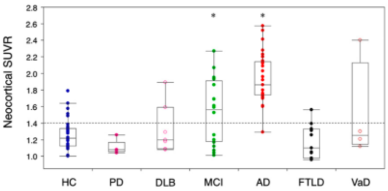

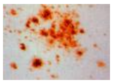

PIB – research tool
Now F-amyloid compounds for clinical use

Villemagne et al, 2011

Tau PET imaging in Dementia (AV1451)

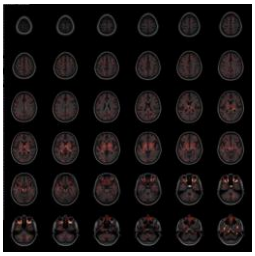

Control

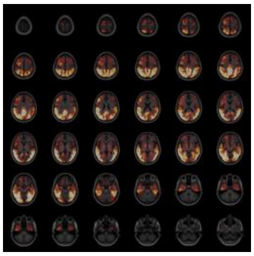

AD

COD: Literature review

- 129 papers identified of which 41 studies (32%) had imaging biomarkers as outcome measures; increase over time (26% of published studies v 54% ongoing protocols, Chi Sq p=0.056)
  - **Serial structural MRI** — **30**
  - **FDG PET** — **13**
  - **Amyloid PET** — **10**
  - **EEG** — **3 (but 1 in VaD and 1 ongoing)**
  - **MR spectroscopy** — **1 (but results not reported)**
  - **Perfusion SPECT** — **1**
  - **Transcranial doppler** — **1**
  - **Tau PET** — **1 (ongoing)**

NB some studies included more than one biomarker

Summary of MR studies

- Whole brain and hippocampal volume change mostly used
- Changes demonstrated over periods 26 to 80 weeks
- Usually similar between groups **but**

- Fox (2005, AN1792): greater atrophy in treated group
- Salloway (2009, ph2 Bapi): greater atrophy in treated group
- Li (2015, CH herb): decrease only in placebo group
- Salloway (2011, ELND005): greater atrophy in treated group
- Turner (2015, resveratrol): greater atrophy in treated group
- Winblad (2012, CAD106): treatment group declined less

Effects of Aβ immunization (AN1792) on MRI measures of cerebral volume in Alzheimer disease

N.C. Fox, MD, FRCP; R.S. Black, MD; S. Gilman, MD, FRCP; M.N. Rossor, MD, FRCP; S.G. Griffith, MD, PhD, MRCP; L. Jenkins, PhD; and M. Koller, MD, MPH, for the AN1792(QS-21)-201 Study Team*

Whole-brain volume boundary shift integral, %				
Placebo	52	2.04 (1.74)		
Antibody responder	38	3.12 (1.98)	−1.01 (−1.74, −0.28)	0.007
Ventricular volume boundary shift integral,† %				
Placebo	56	0.48 (0.40)		
Antibody responder	45	1.10 (0.75)	−0.61 (−0.84, −0.39)	<0.001
Hippocampal volume, %				
Placebo	57	−2.86 (3.19)		
Antibody responder	45	−3.78 (2.63)	0.93 (−0.26, 2.12)	0.124

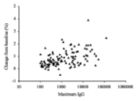

50% greater volume loss in immunised subjects.
Sig correlation with immune response

Fox et al, 2005

A randomized, double-blind, placebo-controlled trial of resveratrol for Alzheimer disease

OPEN

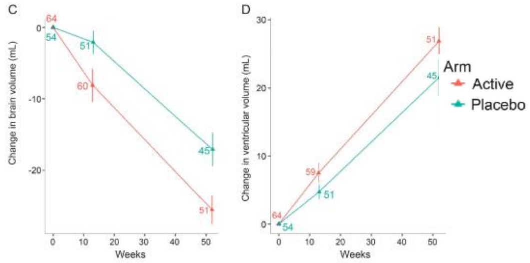

Turner et al, 2015

Summary of FDG studies

- Whole brain and ROI analysis used
- Changes demonstrated over periods 26 to 80 weeks
- Usually similar between groups **but**

- Kadit (2008, phenserine): increase in treatment group
- Dodel (2013, iv IG): greater change in placebo group
- Wang (2013, memantine): treatment group declined less

Effects of Memantine on Clinical Ratings, Fluorodeoxyglucose Positron Emission Tomography Measurements, and Cerebrospinal Fluid Assays in Patients With Moderate to Severe Alzheimer Dementia

A 24-Week, Randomized, Clinical Trial

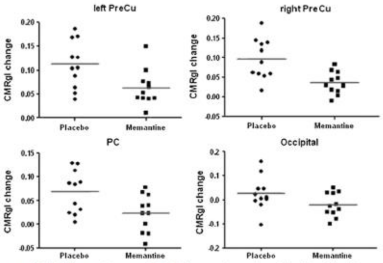

FIGURE 2. Twenty-four-week CMRgl changes in anatomical ROIs known to be preferentially affected by AD.

Wang et al, 2013

Summary of Amyloid studies

- Whole brain SUVR usually used
- PIB and Florbetapir most widely used ligands
- Changes not always demonstrated over time
- Usually similar between groups **but**

- Ostrowitzki (2012, gantenerumab): decrease in higher dose
- Rinne (2010, ph2 Bapi): decrease in treated group
- Salloway (2014, ph3 Bapi): increase only in placebo group

Two Phase 3 Trials of Bapineuzumab in Mild-to-Moderate Alzheimer's Disease

Stephen Salloway, M.D., Reisa Sperling, M.D., Nick C. Fox, M.D., Kaj Blennow, M.D.,

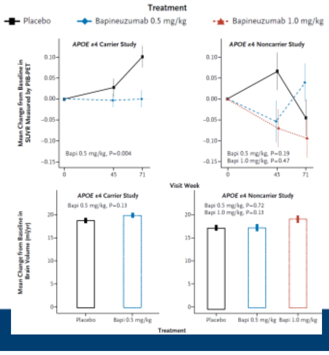

UNIVERSITY OF CAMBRIDGE

Biomarkers: Imaging v CSF

Imaging:

- Better validated for multi-site studies
- Measures pathology directly in target organ (brain)
- Can obtain both whole brain and spatial (regional) information
- Can obtain multi-modal data (structure, tau, amyloid, metabolism) though need > one scan
- People don't mind scans

CSF:

- Cheaper (but how much in reality?)
- Can obtain tau and amyloid info in single step

Imaging the clear winner by 5 points to 2!

UNIVERSITY OF CAMBRIDGE

Imaging biomarkers: Conclusions

	Demonstrable sensitivity to change in untreated patients	Validated against underlying pathology	Available in UK in sufficient sites for use as outcome measure	Potentially useful outcome measure
**Structural MR**	Yes	Yes (mainly tau)	Yes	**Yes**
**MR Spectroscopy**	No	No	Yes	No
**EEG**	No	No	Yes	No
**Doppler**	No	No	Yes	No
**Perfusion SPECT**	Yes	Yes (mainly tau)	Yes	No (FDG PET better)
**FDG PET**	Yes	Yes (mainly tau)	Yes	**Yes**
**Amyloid PET**	Yes (slowly)	Yes (amyloid)	Yes	**Yes**
**Tau PET**	No (but only one study)	Yes (tau)	Not yet (likely to change soon)	Not yet proven but highly likely

UNIVERSITY OF CAMBRIDGE

Thank you!

UNIVERSITY OF CAMBRIDGE

Important factors associated with scan

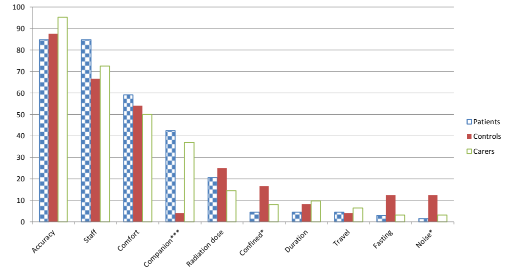

Overall 97% rated PET and 91% SPECT as worthwhile

UNIVERSITY OF CAMBRIDGE

Bamford et al, 2015

Neuropsychiatric symptoms

Gill Livingston

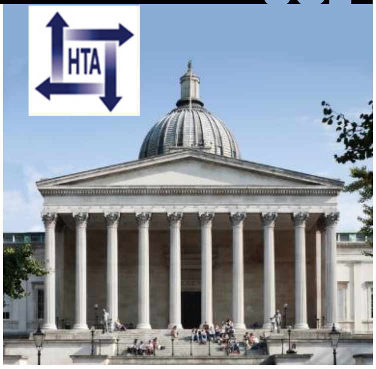

10 minutes

What they are
Should they be core
Desirable characteristics
Potential measures
Frequency of use and time taken
Recommendations and why

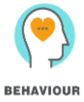

What they are

- abnormal mood,
- disturbed behaviour,
- Disturbed thinking
- Disturbed perception

Also known as BPSD

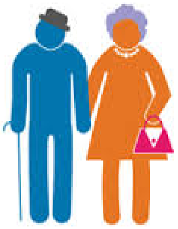

Should they be core

NO

People with mild to moderate dementia may have

- No or no clinically significant neuropsychiatric symptoms
- Therefore no potential for these to improve
  - 78% newly diagnosed patients with AD neuropsychiatric symptoms
  - 59% clinically significant symptoms

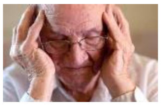

Desirable characteristics

1. Valid and reliable in the population to be tested. This includes content validity -- measure covers neuropsychiatric and only neuropsychiatric items in
2. Frequency of use - valued or practical and how much they are likely to be used in practice.
3. Neuropsychiatric symptoms can be considered in terms of severity or frequency. - the symptoms may improve either by being of reduced frequency or reduced severity or one of these dimensions may improve while the other deteriorates .
4. Time taken should not be too long as the instrument would be part of a package
5. Ideally but not essentially the Minimally Clinically Important difference should have been calculated
6. Ideally translated into different languages

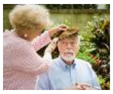

Potential measures

1. The Neuropsychiatric Inventory(NPI),
2. Brief Psychiatric rating Scale (BPRS),
3. Alzheimer's Disease Assessment Scale - Non Cognitive Scale (ADAS-non-cog) ,
4. CERAD Behavioural rating scale for dementia,
5. The Revised Memory and Behavior Problems checklist,
6. Dysfunctional Behavior Rating Instrument
7. BEHAVE-AD
8. Nurses Observation Scale for Geriatric Patients
9. Plutchik Geriatric Rating Scale.

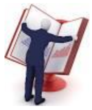

	Number of studies	Number published	Number ongoing	Number of participants	Time taken
NPI	39	31	8	11613	10-20 minutes
ADAS-noncog	7	7	0	778	20-25 minutes
BPRS	3	3	0	190	20 minutes
Nurses Observation Scale for Geriatric Patients	2	2	0	2109	3-5 minutes
CERAD Behavioural rating scale for dementia	1	1	0	486	20-30 minutes
BEHAVE-AD	1	1	0	425	20 minutes
Dysfunctional Behavior Rating Instrument (DBRI)	1	1	0	406	20 minutes
Plutchik Geriatric Rating Scale	1	1	0	178	5-10 minutes
Revised memory and behavior problems checklist.	1	1	0	170	10 minutes

What’s in

NPI- valid, reliable, Frequency and severity

Frequent use. MCID =8

Only severity

BEHAVE AD

ADAS non-cog

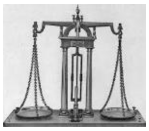

What's out

The rest

August, 2014

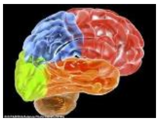

Recommendations

Neuropsychiatric Inventory

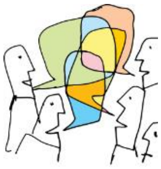

What do you think?

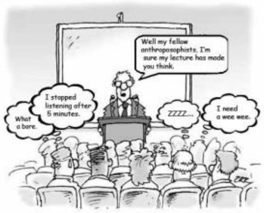

Quality of life as a core outcome in dementia

SUBE BANERJEE

PROFESSOR OF DEMENTIA
CENTRE FOR DEMENTIA STUDIES
BRIGHTON AND SUSSEX MEDICAL SCHOOL

Health related quality of life

...an <u>individual's perception</u> of their position in life...in relation to their goals, expectations, standards and concerns

– WHOQOL

...the <u>impact of a perceived health state</u> on [living] a subjectively fulfilling life

– Bullinger et al 1993

Quality of life -- particularly important in dementia

- Dementias are more complex than simple disorders
  – coronary heart disease
  – diabetes
  – surgical care
- Non-linear and unpredictable process
- Multiple pathologies
- Link between symptoms and qol is not clear, simple or predictable
- Interventions are usually complex
  – need to avoid OSPD syndrome

Case of including measures of quality of life in dementia

Conceptual

- **Does it measure what we are interest in?**
- What really matters to people with dementia and their carers
- What really matters to clinicians
- The true goal of intervention
- Therefore relevant to policy makers and researchers

Scientific

- **Does it add any new and useful intelligence?**
- Can you do it, does it work?
- More than just a combination of health assessments
- cognition and activity limitation are not very good proxies for quality of life

DEMQOL-Proxy: Pearson correlation with clinical measures

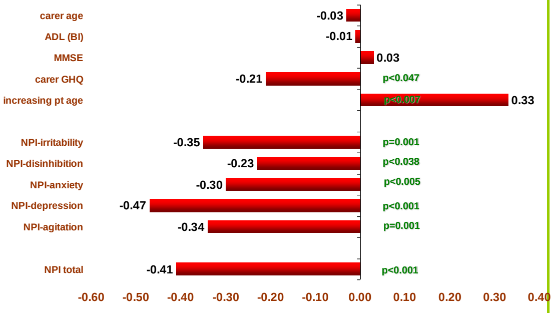

Banerjee et al (2006) JNNP

DEMQOL-Proxy: Linear regression of clinical variables with DEMQOL-Proxy

	Beta coefficient	p-value
NPI total	0.52	<0.001
Patient age	0.32	0.016
MMSE	-0.16	0.281
Carer GHQ	0.07	0.623
ADL (BI)	0.02	0.924

Banerjee et al (2006) JNNP

Two approaches to HRQL measurement

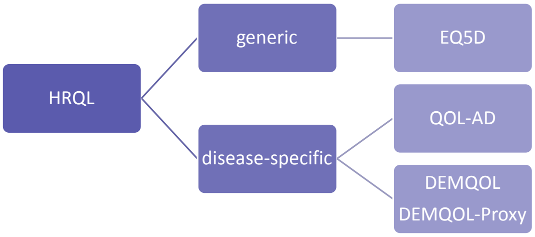

EQ-5D

- Generic measure of HRQL
- Designed for use in all populations
- Questions about its validity in dementia (common with all generic HRQL instruments)
- Provides a simple descriptive profile and a single index value for health status
- In a health profile, respondents describe their current health state in 5 dimensions (EQ-5D descriptive system):
  - mobility,
  - self-care,
  - usual-activities (UA),
  - pain/discomfort (P/D), and
  - anxiety/depression (A/D)

Scoring: the EQ-5D index

- Each of the 5 dimensions are classified as: none (1), moderate (2), or extreme (3)
- The patient's responses result in a 5-digit number
  - So ''11123'' indicates no problems with mobility, self-care, or usual activities but moderate problems with pain/discomfort and extreme problems with anxiety/depression)
- Overall, 243 health states are possible
- A preference-based index score can be calculated based on the EQ-5D health state combined with weightings derived from a sample of the general population. The EQ-5D index reflects the general population's valuation of the health state

Disease-specific measures of HRQL

- Much excellent work in instrument development eg
  - **PDS** Dejong et al (1989)
  - **DQOL** Brod et al (1999)
  - **QOL-AD** Logsdon et al (1999)
  - **ADRQL** Black et al (2000)
  - **QOLAS** Selai et al (2001)
  - **DEMQOL** Smith et al (2005)
- Evolving field with progressive refinement of methodology
- **Development from measures of cognition or activity limitation to measures of HRQL**

QOL-AD

- 13-item measure patient's quality of life from both the patient and the caregiver
- Developed for individuals with dementia, based on patient, caregiver, and expert input, to maximize construct validity
- It uses simple and straightforward language and responses
- Rated on a four point scale, with 1 being poor and 4 being excellent. Total scores range from 13 to 52.
- Caregivers about 5 minutes, people with dementia 10-15 minutes to administer (same form)

QOL-AD

- 1. First of all, how do you feel about your physical health? Would you say it's poor, fair, good, or excellent? Circle whichever word you think best describes your physical health right now.
- 2. How do you feel about your energy level? Do you think it is poor, fair, good, or excellent?
- 3. How has your mood been lately? Have your spirits been good, or have you been feeling down? Would you rate your mood as poor, fair, good, or excellent?
- 4. How about your living situation? How do you feel about the place you live now? Would you say it's poor, fair, good, or excellent?

Quality of Life: AD
(Interview Version for the person with dementia)

Interviewer administer according to standard instructions. Circle responses.				
1. Physical health.	Poor	Fair	Good	Excellent
2. Energy.	Poor	Fair	Good	Excellent
3. Mood.	Poor	Fair	Good	Excellent
4. Living situation.	Poor	Fair	Good	Excellent
5. Memory.	Poor	Fair	Good	Excellent
6. Family.	Poor	Fair	Good	Excellent
7. Marriage.	Poor	Fair	Good	Excellent
8. Friends.	Poor	Fair	Good	Excellent
9. Self as a whole.	Poor	Fair	Good	Excellent
10. Ability to do chores around the house.	Poor	Fair	Good	Excellent
11. Ability to do things for fun.	Poor	Fair	Good	Excellent
12. Money.	Poor	Fair	Good	Excellent
13. Life as a whole.	Poor	Fair	Good	Excellent

DEMQOL

- Two interviewer administered self-report instruments
  - different items work in the two groups
  - measuring the same thing
- DEMQOL
  - 28 item self report for people with dementia
  - 5 to 30 minutes
  - Score 28 to 112
- DEMQOL-Proxy
  - 31 item carer report on qol of person with dementia
  - 5 to 10 minutes
  - Score 31 to 124
- Administration manuals for each

Study ID

DEMQOL

Instructions: *Read each of the following questions (in bold) verbatim and show the respondent the response card.*

I would like to ask you about your life. There are no right or wrong answers. Just give the answer that best describes how you have felt in the last week. Don't worry if some questions appear not to apply to you. We have to ask the same questions of everybody.

Before we start we'll do a practise question; that's one that doesn't count. *(Show the response card and ask respondent to say or point to the answer.)*. **In the last week, how much have you enjoyed watching television?**

a lot **quite a bit** **a little** **not at all**

Follow up with a prompt question: **Why is that?** *or* **Tell me a bit more about that.**

First I'm going to ask about <u>your feelings</u>. In the last week, have you felt…….

1. **cheerful? ****	☐ a lot	☐ quite a bit	☐ a little	☐ not at all
2. **worried or anxious?**	☐ a lot	☐ quite a bit	☐ a little	☐ not at all
3. **that you are enjoying life? ****	☐ a lot	☐ quite a bit	☐ a little	☐ not at all
4. **frustrated?**	☐ a lot	☐ quite a bit	☐ a little	☐ not at all
5. **confident? ****	☐ a lot	☐ quite a bit	☐ a little	☐ not at all
6. **full of energy? ****	☐ a lot	☐ quite a bit	☐ a little	☐ not at all
7. **sad?**	☐ a lot	☐ quite a bit	☐ a little	☐ not at all
8. **lonely?**	☐ a lot	☐ quite a bit	☐ a little	☐ not at all
9. **distressed?**	☐ a lot	☐ quite a bit	☐ a little	☐ not at all
10. **lively? ****	☐ a lot	☐ quite a bit	☐ a little	☐ not at all
11. **irritable?**	☐ a lot	☐ quite a bit	☐ a little	☐ not at all
12. **fed-up?**	☐ a lot	☐ quite a bit	☐ a little	☐ not at all
13. **that there are things that you wanted to do but couldn't?**	☐ a lot	☐ quite a bit	☐ a little	☐ not at all

Next, I'm going to ask you about <u>your memory</u>. In the last week, how worried have you been about……….

14. **forgetting things that happened recently?**	☐ a lot	☐ quite a bit	☐ a little	☐ not at all
15. **forgetting who people are?**	☐ a lot	☐ quite a bit	☐ a little	☐ not at all
16. **forgetting what day it is?**	☐ a lot	☐ quite a bit	☐ a little	☐ not at all

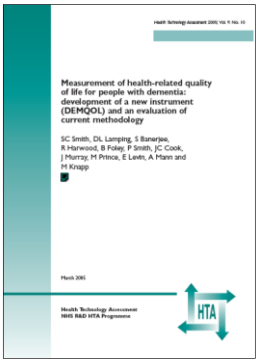

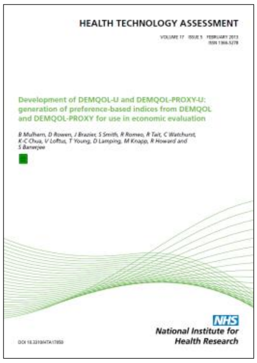

Smith et al (2005); Mulhern et al (2013). Health Tech Ass

Summary of psychometric properties of instruments by gold standard criteria

	PDS	Pleasant Events Schedule – AD	QOL-AD		DEMQOL	
			Patient	Proxy	Patient	Proxy
**Conceptual model**	0	+	++	0	+++	+++
**Acceptability**	0	0	++	++	++	++
**Reliability**						
**Internal consistency**	0	0	+++	+++	+++	+++
**Test–retest**	+++	0	+++	++	+++	+++
**Inter-rater reliability**	0	0	NA	0	+	+
**Validity**						
**Content**	+	0	+++	+++	+++	+++
**Criterion-related**	0	0	0	0	++	++
**Construct**						
**Convergent validity**	0	+	+++	+++	+++	+++
**Discriminant validity**	0	0	0	0	++	++
**Known groups differences**	+	0	+++	0	+++	+++
**Experimental intervention**	0	0	++	++	++	++
**Factor analysis**	0	0	+	+	++	++
**Responsiveness**	0	0	+	+	+	+
**Respondent burden**	0	++	+++	+++	+++	+++
**Cultural/language adaptations**	0	0	++	++	++	++
**Economic evaluation**	0	0	0	0	+++	+++
**Health state classification**	0	0	0	0	+++	+++
**Preference-based measures**	0	0	0	0	+++	+++
**Population values**	0	0	0	0	+++	+++

0, no evidence or not tested; +, some limited evidence; ++, some good evidence, but some aspects do not meet criteria or some aspects not tested/reported; +++, good evidence; NA, not applicable.

Data from systematic review on use

	EQ5D	QOL-AD	DEMQOL
Number studies	5	9	4
Studies published	4	7	1
Studies ongoing	1	2	3
Number participants	4084	4893	399

Choices

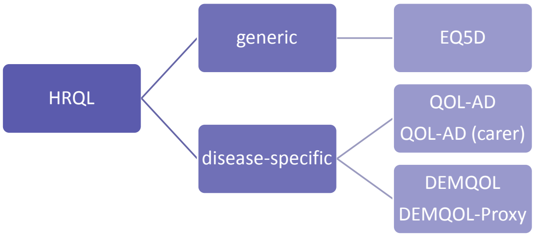

HRQL
generic
disease-specific
EQ5D
QOL-AD
QOL-AD (carer)
DEMQOL
DEMQOL-Proxy
